# Dual Role for Pilus in Adherence to Epithelial Cells and Biofilm Formation in *Streptococcus agalactiae*

**DOI:** 10.1371/journal.ppat.1000422

**Published:** 2009-05-08

**Authors:** Yoan Konto-Ghiorghi, Emilie Mairey, Adeline Mallet, Guillaume Duménil, Elise Caliot, Patrick Trieu-Cuot, Shaynoor Dramsi

**Affiliations:** 1 Institut Pasteur, Unité de Biologie des Bactéries Pathogènes à Gram-Positif and URA CNRS 2172, Paris, France; 2 Institut National de la Santé et de la Recherche Médicale (INSERM) U570, Université Paris, Faculté de Médecine René Descartes, UMR- S570, Paris, France; 3 Imagopole, Plate-forme de Microscopie Ultrastructurale, Institut Pasteur, Paris, France; Schepens Eye Research Institute, United States of America

## Abstract

*Streptococcus agalactiae* is a common human commensal and a major life-threatening pathogen in neonates. Adherence to host epithelial cells is the first critical step of the infectious process. Pili have been observed on the surface of several gram-positive bacteria including *S. agalactiae*. We previously characterized the pilus-encoding operon *gbs1479-1474* in strain NEM316. This pilus is composed of three structural subunit proteins: Gbs1478 (PilA), Gbs1477 (PilB), and Gbs1474 (PilC), and its assembly involves two class C sortases (SrtC3 and SrtC4). PilB, the bona fide pilin, is the major component; PilA, the pilus associated adhesin, and PilC, are both accessory proteins incorporated into the pilus backbone. We first addressed the role of the housekeeping sortase A in pilus biogenesis and showed that it is essential for the covalent anchoring of the pilus fiber to the peptidoglycan. We next aimed at understanding the role of the pilus fiber in bacterial adherence and at resolving the paradox of an adhesive but dispensable pilus. Combining immunoblotting and electron microscopy analyses, we showed that the PilB fiber is essential for efficient PilA display on the surface of the capsulated strain NEM316. We then demonstrated that pilus integrity becomes critical for adherence to respiratory epithelial cells under flow-conditions mimicking an *in vivo* situation and revealing the limitations of the commonly used static adherence model. Interestingly, PilA exhibits a von Willebrand adhesion domain (VWA) found in many extracellular eucaryotic proteins. We show here that the VWA domain of PilA is essential for its adhesive function, demonstrating for the first time the functionality of a prokaryotic VWA homolog. Furthermore, the auto aggregative phenotype of NEM316 observed in standing liquid culture was strongly reduced in all three individual pilus mutants. *S. agalactiae* strain NEM316 was able to form biofilm in microtiter plate and, strikingly, the PilA and PilB mutants were strongly impaired in biofilm formation. Surprisingly, the VWA domain involved in adherence to epithelial cells was not required for biofilm formation.

## Author Summary

*Streptococcus agalactiae* (Group B Streptococcus) is a leading cause of sepsis (blood infection) and meningitis (brain infection) in newborns. Most bacterial pathogens have long filamentous structures known as pili or fimbriae, which are often involved in the initial adhesion of bacteria to host tissues but also in bacteria–bacteria interactions, resulting in biofilm formation. Our previous functional characterization of the pilus locus in *S. agalactiae* showed that it encodes a major pilin and two minor pilin subunits that are covalently polymerized by the action of two enzymes belonging to the sortase C family. One of the accessory pilins is responsible for the adhesive property of the pilus. However, this initial study raised two major questions that were addressed in the present work: i) what anchors the pilus to the cell wall and ii) what is the function of the pilus fiber itself. We showed that the pilus is essential for optimal display of the pilus-associated adhesin and overcomes the masking effect of the capsule. Pilus integrity was shown to be critical in adherence assays under flow conditions. We also report that GBS can form biofilms and that pili play an important role in this process.

## Introduction

Group B Streptococcus (GBS, *Streptococcus agalactiae*) is a common colonizer of the gastro-intestinal and urogenital tracts of up to 40% of healthy individuals [1]. However, in certain circumstances, GBS can become a life-threatening pathogen causing invasive infections in human neonates [2],[3]. Epidemiological studies have documented how commonly GBS are transmitted from “carrier” mothers to newborn infants [4]. The clinical symptoms of acute GBS disease are pneumonia, septicemia, and meningitis. The lung is the portal of entry in neonatal GBS infections although it possesses a sophisticated array of innate immune mechanisms for defense against infection: mechanical barriers and mucociliary clearance, antimicrobial factors in the airway lining fluid, and resident alveolar macrophages. Thus, adherence to the host pulmonary epithelium is the first step in GBS pathogenesis and experimental studies involving static binding assays indicate that the molecular interactions of *S. agalactiae* with host cells are complex, involving a variety of surface adhesion molecules [5],[6],[7].

Bacterial pili have recently been recognized in several gram-positive bacteria (for reviews see [8]–[12]). In contrast to gram-negative bacteria, gram-positive bacteria assemble pili by a distinct mechanism involving a transpeptidase called sortase. Sortase was first discovered in *Staphylococcus aureus* and is mostly known for catalyzing the covalent anchoring of LPXTG-containing proteins to the peptidoglycan [13]. Analysis of bacterial genomes revealed a plethora of sortases in almost all gram-positive species with frequently more than one sortase gene per genome [14]. Our previous bioinformatic analysis of sixty-one sortases from complete gram-positive genomes suggested the existence of 4 distinct classes of sortases named A, B, C, and D involved in different functions [15]. The class A sortase is the ubiquitous housekeeping enzyme that anchors LPXTG proteins to the cell wall. The class B, C, and D sortases are specifically involved in iron acquisition, pilus assembly and developmental processes including sporulation [15]–[17].

Sortase-mediated pilus assembly was first demonstrated in *Corynebacterium diphtheriae* [18],[19] and these pioneer studies revealed the existence of 3 conserved genetic elements found within the major pilin subunit and necessary for pilus formation: i) the pilin motif (WxxxVxVYPK); ii) the E-box domain (YxLxETxAPxGY); and iii) the cell wall sorting signal (LPxTG followed by a hydrophobic domain and a positively charged tail). The current model for pilus assembly is as follows: the major subunit is assembled into a pilus by a *cis*-encoded sortase that catalyzes the covalent attachment between the conserved pilin motif lysine residue of one subunit with the conserved threonyl residue LPxTG motif of another subunit. In addition, one or more accessory subunits are incorporated into the pilus by an unknown mechanism, but requiring pilus-specific sortase as well as the E-box domain within the major pilin subunit. Then, during a final step, the pilus fiber is covalently linked to the peptidoglycan by either the pilus-specific sortase or the housekeeping sortase. This mechanism of pilus assembly catalyzed by class C sortases has now been demonstrated in several gram-positive pathogens using similar genetic and biochemical analyses [20]–[27].

We previously carried out a detailed structural and functional analysis of the pilus locus *gbs1479-1474* (also referred to as PI-2A [27] in GBS strain NEM316 [22]). This locus encodes a pilus composed of three structural subunit proteins Gbs1478 (PilA), Gbs1477 (PilB), and Gbs1474 (PilC) whose assembly involves two class C sortases (SrtC3 and SrtC4). PilB, the *bona fide* pilin, is the major component; PilC is a minor associated component mainly localized at the base of the pilus; and PilA is the pilus associated adhesin located at intervals along the pilus backbone. We previously showed that PilA mediates adherence of GBS NEM316 to the pulmonary epithelial cell line A549 independently of pilus formation [22]. The apparently paradoxical situation of a pilus that carries the adhesive property and yet is dispensable for binding was reported previously in the *Escherichia coli* Pap pilus model system [28] and more recently in the pneumococcal pilus [29]. We postulated that, in the absence of pilus, PilA behaves as a classical LPXTG-containing adhesin anchored to the cell wall by the housekeeping class A sortase SrtA to mediate adherence to cultured epithelial cells.

Bacteria often exist within natural systems in an entirely different form (sessile) from those grown in laboratory conditions (planktonic). Sessile bacteria appear to be protected in hostile environments by growing as colonies embedded in an extracellular matrix of carbohydrate or exopolysaccharide called biofilm. The pattern of biofilm development involves bacterial attachment to a solid surface, the formation of microcolonies, and their differentiation into exopolysaccharide-encased communities to form a mature biofilm. Many gram-negative pathogens use their pili to promote attachment and aggregation to host cells, that eventually develop into mature biofilm resulting in host tissues colonization [30]. Pilus contribution in biofilm formation was recently shown in gram-positive bacteria such as *E. faecalis*, *S. pyogenes* (GAS), and *S. pneumoniae* [11],[26],[31],[32].

In this work, we investigated the roles of the housekeeping sortase A in pilus assembly in GBS and that of the pilus structure to resolve the paradox of a pilus dispensable in adherence assays although containing an adhesin subunit. We characterized the functional role of the von Willebrand adhesion domain found in the PilA adhesin. We adapted a biofilm formation assay for GBS and thus uncovered an essential role of GBS pilus in this process.

## Results

### The pilus is anchored to the cell-wall by the housekeeping class A sortase

Our previous functional characterization of the pilus locus in *S. agalactiae* [22] raised the question of the role of the housekeeping class A sortase (SrtA) in pilus biosynthesis but did not answer it since transcription of the PI-2A pilus locus was dramatically reduced in the *srtA* mutant of strain NEM316 [22]. This mutant was made by insertion of a promoterless *aphA-3* kanamycin cassette within *srtA* by allelic replacement to generate a strain that synthesizes a truncated SrtA protein deleted of its carboxylic half (i.e., 127 out of 248 amino acids) including the catalytic TLXTC sequence [33]. Complementation of the *srtA* mutant with the wild-type gene inserted ectopically on NEM316 chromosome did not result in wild-type levels of pilus expression (data not shown), although restoring the correct localization of two model LPXTG proteins, Alp2 and ScpB [33].

**Figure 1:**
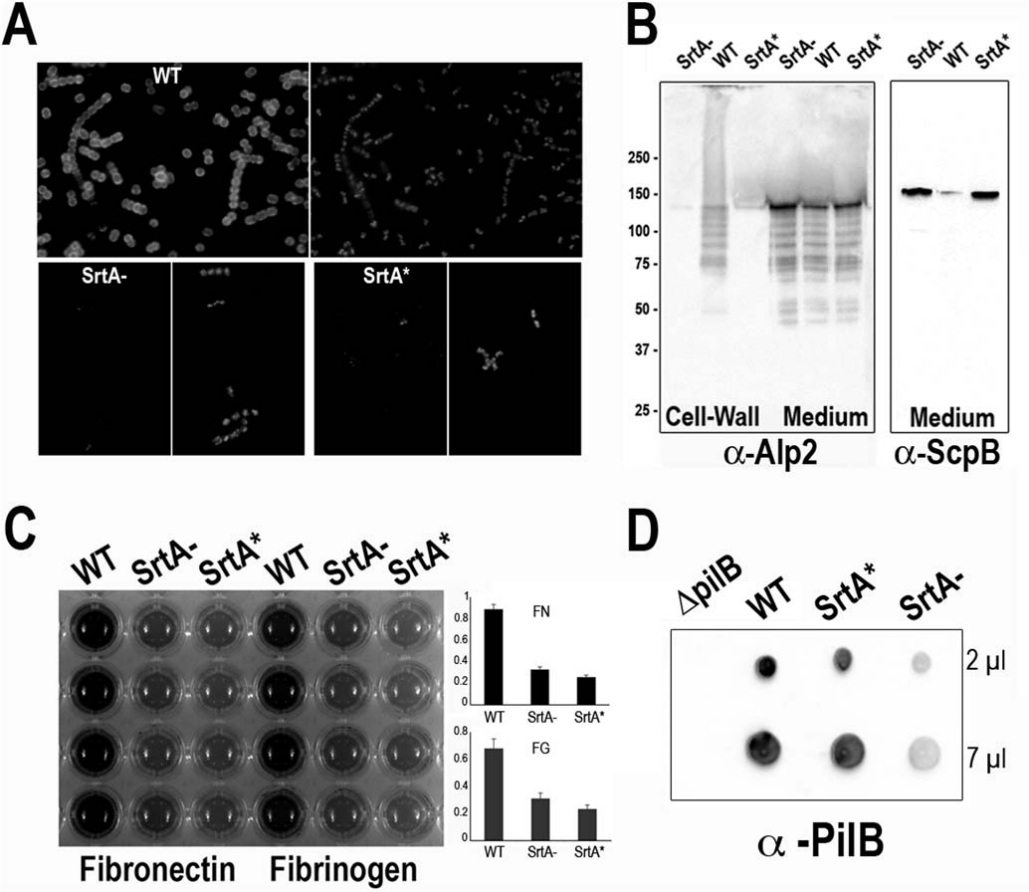
Phenotypic characterization of the catalytic mutant of Sortase A (SrtA*). (A) Display of the LPXTG-containing protein Alp2 on the cell surface of *S. agalactiae* NEM316 (wild-type strain), NEM2135 (SrtA^−^ mutant), and NEM2511 (SrtA^*^). Bacteria were grown in TH broth at 37°C to an OD_600_≈1.5 washed twice in PBS, and incubated for 5 min at 37°C in PBS containing 1% SDS. They were analyzed by immunofluorescence with affinity-purified polyclonal anti-R28/Alp2 antibodies revealed with an anti-IgG coupled to Alexa 488 (left panel) and the same sample was observed by staining the DNA with DAPI (right panel). (B) Western blot analysis of GBS cell wall and supernatant proteins with anti-R28/Alp2 and anti-ScpB sera from the wild-type strain NEM316, the SrtA^−^ mutant, and the catalytic mutant SrtA^*^. (C) Adherence of *S. agalactiae* strains to immobilized human fibronectin and fibrinogen. Microtiter wells were coated with 5 µg of fibronectin or fibrinogen and 10^7^ bacterial CFU (NEM316, SrtA^−^ and SrtA* strains) were added. The wells were washed and bound bacteria were assayed with crystal violet staining. OD_595_ values are presented as mean values (±SD) of two experiments performed in quadruplet. (D) Dot-blot analysis of PilB expression on whole bacteria in wild-type NEM316 and the *srtA* mutants. The isogenic *ΔpilB* mutant was used to verify the antibody specificity.

To characterize the role of SrtA in pilus synthesis, we therefore constructed a catalytic mutant of SrtA by in frame-modification of the TLXTC signature sequence encompassing the critical cysteyl residue (TLVTCTDPE to TAAAPGRAE replacement in the catalytic site). This new mutant named SrtA* exhibited phenotypes similar to those of the previously characterized SrtA^−^ mutant (Figure 1A–1C). It is unable to anchor the classical LPXTG protein Alp2 on the bacterial surface as shown by immunofluorescence (Figure 1A) or by Western blotting (Figure 1B, left panel). The ScpB protein was found in larger amounts in the supernatants of the SrtA^−^ and SrtA* mutants compared to the wild-type strain NEM316 (Figure 1B, right panel). As expected, the binding to human fibronectin- and fibrinogen-coated plates was similarly affected in both mutant strains (Figure 1C). Of note, the surface properties of SrtA^−^ and SrtA* were macroscopically different from that of the parental strain: they bound less to polypropylene-, MaxiSorp-, or glass-matrices and their pellets obtained after centrifugation were smooth (data not shown).

**Figure 2:**
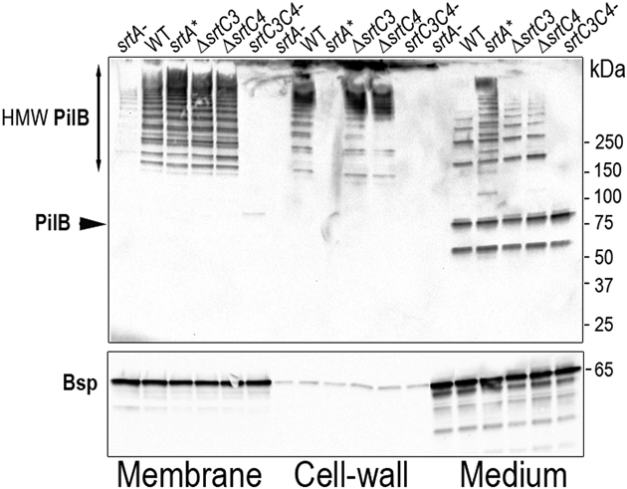
Pilus synthesis and localization in *S. agalactiae* sortases mutants. Proteins anchored to the cell-wall or associated to the membrane or secreted in the supernatant were isolated from *S. agalactiae* strain NEM316 and its isogenic sortases mutants [22], separated on 4%–12% Criterion XT SDS-PAGE gel, and detected by immunoblotting with specific anti PilB antiserum (upper part). Equivalent amount corresponding to 500 µl of the initial culture was loaded in each well. The PilB monomer is indicated by a black arrow. The high-molecular-weight species correspond to PilB polymers while the lower band at 50 kDa is most likely a degradation product. The equal quantity loaded in each well is verified by immunoblotting the same gel with a control antiserum that recognizes a secreted protein Bsp of 65 kDa (lower part).

We then tested expression levels of the major pilin subunit PilB in the SrtA* mutant by immunoblotting on whole bacteria. As shown in Figure 1D, the level of PilB in the SrtA* mutant was similar to that found in the wild-type strain. To unravel the role of the various sortases in the pilus assembly process, we monitored pilus polymerization in the various GBS sortases mutants by immunoblotting using specific anti-PilB polyclonal antibody (Figure 2). *S. agalactiae* isogenic strains were grown to the same optical density (OD_600_≈2) and the cultures were separated into three fractions (medium, cell-wall, and membrane) that were electrophoresed on 4–12% gradient SDS-PAGE and probed with the PilB antiserum (upper panel). The same fractions were also probed with an antiserum raised against the secreted protein Bsp [34] used as an internal loading control (lower panel). Pilus polymers are readily detected in the various fractions of the wild-type strain but, as previously reported [22], their polymerization requires either SrtC3 or SrtC4 (Figure 2). PilB monomer could be detected in the culture medium fractions as a band of about 80 kDa, the lower band at 60 kDa being a degradation product. As previously shown, pili are not expressed in the SrtA^−^ mutant (Figure 2). In the SrtA* mutant, the pilus polymers are only found in the membrane and medium fractions, but not in the cell wall fraction. This result demonstrates that the housekeeping class A sortase is not necessary for pilus polymerization but is absolutely required for anchoring the pilus to the cell wall.

### Pilus integrity is essential for PilA display at the cell surface

**Figure 3:**
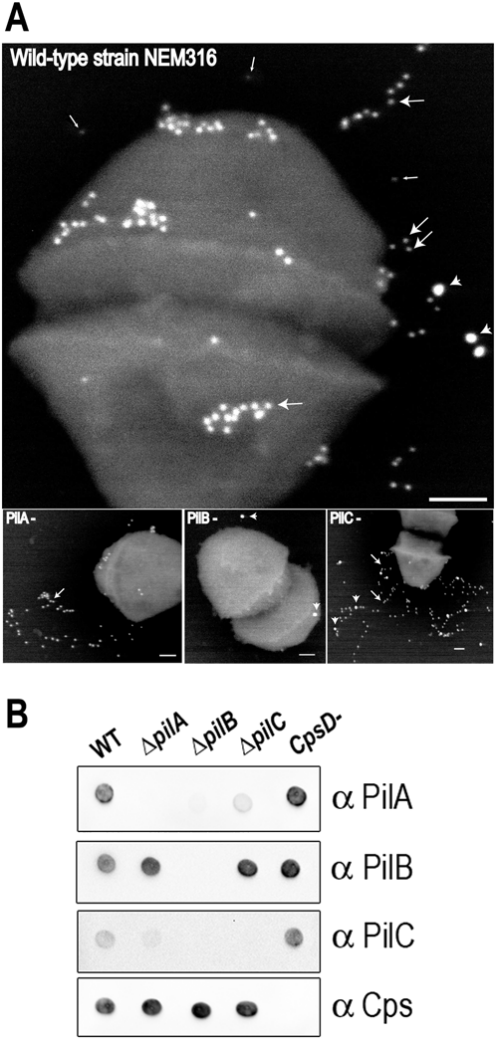
The PilB pilus backbone as a carrier for surface display of the pilus-associated adhesin PilA. (A) Immuno-electron-microscopy (IEM) analyses of the pilus subunits PilA, PilB and the capsular type III polysaccharide. *S. agalactiae* wild-type strain NEM316 and its isogenic pilus mutants (*ΔpilA*, *ΔpilB*, *ΔpilC*) were incubated with rabbit polyclonal antibody raised against PilA and PilB, and with a mouse monoclonal antibody raised against the type III capsular polysaccharide (mAb S9). Antibodies were conjugated to 20 nm, 10 nm, and 5 nm gold particles, respectively. The outer layer of the capsule is marked by thin arrows. The major pilin PilB is marked by thick arrows. The PilA adhesin is present along the entire pilus and marked by arrowheads (cf. the *ΔpilC* panel). (B) Dot-blot analysis of PilA, PilB, PilC, and capsule expression on whole bacteria in wild-type NEM316 and its isogenic derivatives mutants.

The PI-2A pilus of *S. agalactiae* is composed of three structural subunits PilA (Gbs1478), PilB (Gbs1477), and PilC (Gbs1474). PilB is the major constituent of the pilus fiber; PilC is a minor associated component mainly localized at the base of the pilus; and PilA is the pilus-associated adhesin located at intervals along the pilus backbone [22],[27]. Immunogold electron microscopy revealed abundant surface staining and pilus structures extending largely beyond the capsule in strain NEM316 (Figure 3A). Using the previously characterized mouse monoclonal antibody S9 directed against the type III capsular polysaccharide [35], we carried out a triple-labeling experiment to detect simultaneously the PilB pilin, the PilA-associated adhesin, and the capsule. Wild-type (WT) and isogenic mutant bacteria were stained with: i) mouse mAb S9 followed by 5 nm gold-labeled IgG; ii) with rabbit pAb anti-PilB followed by 10 nm gold-labeled IgG, and iii) with rabbit pAb anti PilA followed by 20 nm gold-labeled IgG. The mAb S9 decorates the external layer of the capsule [36] and its thickness in strain NEM316 was estimated to be ≈50 nm on ultra-thin sections by transmission electron microscopy (Figure S1). In the absence of the PilB backbone pilin, the PilA adhesin is found at the cell surface without detectable pili (Figure 3A, bottom panel). As expected, the absence of the PilA accessory protein did not prevent pilus formation and in this mutant pili are even longer than in wild-type strain (Figure 3A, bottom panel, and Figure S2). Strikingly, in the absence of the PilC ancillary protein, pili are longer but also more extended (Figure 3A, bottom panel). Of note, a significant amount of pili produced by *ΔpilA* and *ΔpilC* mutants were released in the culture medium compared to the parental strain (Figure S2). These immuno electron micrographs were subjected to quantitative analysis and the results are shown in Table 1. Pili were shown to be longer in both *ΔpilA* and *ΔpilC* compared to the wild-type strain. In addition, immunofluorescence analyses clearly shows that pili are not only longer but also thicker in the *ΔpilA* and *ΔpilC* (Figure S2). Immunoblotting analysis on whole bacteria confirmed the specificity of all four antisera (PilA, PilB, PilC, and S9) and showed that in the absence of the pilus backbone (*ΔpilB*), PilA cannot be detected at the bacterial surface (Figure 3B). PilA accessibility at the bacterial surface is also reduced in the *ΔpilC* mutant. Previous transcriptional and western blot analyses showed that deletion of *pilB* or *pilC* does not affect expression of *pilA* [22]. Altogether, these results reinforce the idea that pilus integrity is essential for efficient PilA display at the bacterial surface.

**Table 1:** Quantification of pilus length in *S. agalactiae* NEM316 and mutant derivatives.

	Strain		
	WT	Δ*pilA*	Δ*pilC*
Experiment 1			
Sample number	48	39	50
Mean length in µM	0.29	0.42*	0.65*
Standard Deviation	0.134	0.155	0.299
Experiment 2			
Sample number	25	38	28
Mean length in µM	0.296	0.503*	0.582*
Standard Deviation	0.107	0.185	0.243

*: Using the Mann-Whitney Test, the two-tailed P value is <0.0001 between WT and mutant strains considered extremely significant.

### PilB-dependent display of PilA is essential for adherence to epithelial cells under flow-conditions

**Figure 4:**
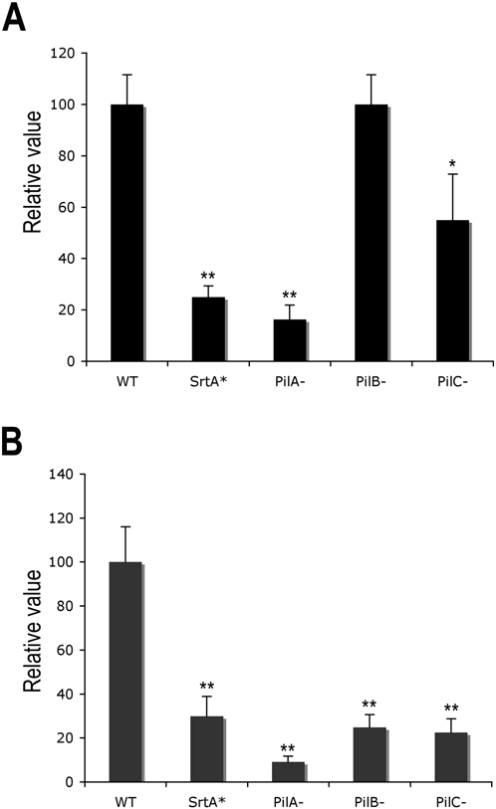
Adherence of *S. agalactiae* pilus mutants to human pulmonary epithelial cells A549 under static (A) or low flow conditions (B). (A) Cells were infected at a M.O.I of 20 bacteria per cell for 1 h at 37°C and adherence frequencies were calculated from the numbers of bacteria remaining attached to the cells after the incubation period with respect to the number if inoculated bacteria. The level of adherence of the WT strain is arbitrarily reported as 100, and the level of adherence of the various mutants are relative values. The results are presented as mean value (±SD) from one representative experiment of at least 3 independent experiments. (B) Adherence of the same strains labeled with fluorescent CMFDA under liquid flow (0.04 dynes/cm^2^). The number of fluorescent bacteria per mm^2^ was determined (see Experimental Procedures). The level of adherence of the WT strain is arbitrarily reported as 100 and the level of adherence of the various mutants are relative values. The results presented as mean value (±SD) is representative of 3 independent experiments.

We previously showed that PilA mediates adherence of *S. agalactiae* strain NEM316 to the human alveolar epithelial cell line A549 independently of pilus formation [22]. Indeed, the apiliated *pilB* mutant is as adherent as the wild-type strain to A549 cells (Figure 4A) and the role of the pilus fiber in bacterial adhesion therefore remains to be characterized. A major defence in the lung is constituted by the mucociliary clearance apparatus. Goblet and glandular cells beneath the epithelium produce mucus that lines the epithelial layer of the air conducting pathways. Mucus is moved through the conducting pathways as fast as 1 cm/min by bronchial epithelial cell cilia to the trachea and later towards the mouth. We reasoned that surface display of PilA adhesin could be important in more stringent adherent conditions, e.g., in the presence of liquid fluid mimicking the mucociliary movement in the lung. A major limitation of the standard adhesion model is that it neglects the local fluid mechanic environment encountered in the organism. We therefore examined the role of the various pilus components under defined shear stress condition by analyzing *pilA*, *pilB*, and *pilC* mutants. Adherent human alveolar epithelial cells (A549) were grown on glass slides and placed in a laminar flow chamber observed under an inverted microscope (for experimental details see [37]). *S. agalactiae* labeled with fluorescent 5-chloromethylfluorescein diacetate (CMFDA) was introduced in the chamber under a controlled flow. Before introduction of bacteria little or no fluorescence was detected. A low shear stress value (0.04 dynes/cm^2^) mimicking the mucus flow in the lung was selected. We showed that all three pilus mutants were significantly decreased for adherence as much as the srtA* mutant under low shear stress (Figure 4B and Figure S3). The structural component of the pilus is therefore necessary for efficient adhesion in the presence of a shear stress reproducing the conditions encountered by the bacteria in the lung.

### The von Willebrand Adhesion type A (VWA) domain of PilA is required for adherence to epithelial cells

**Figure 5:**
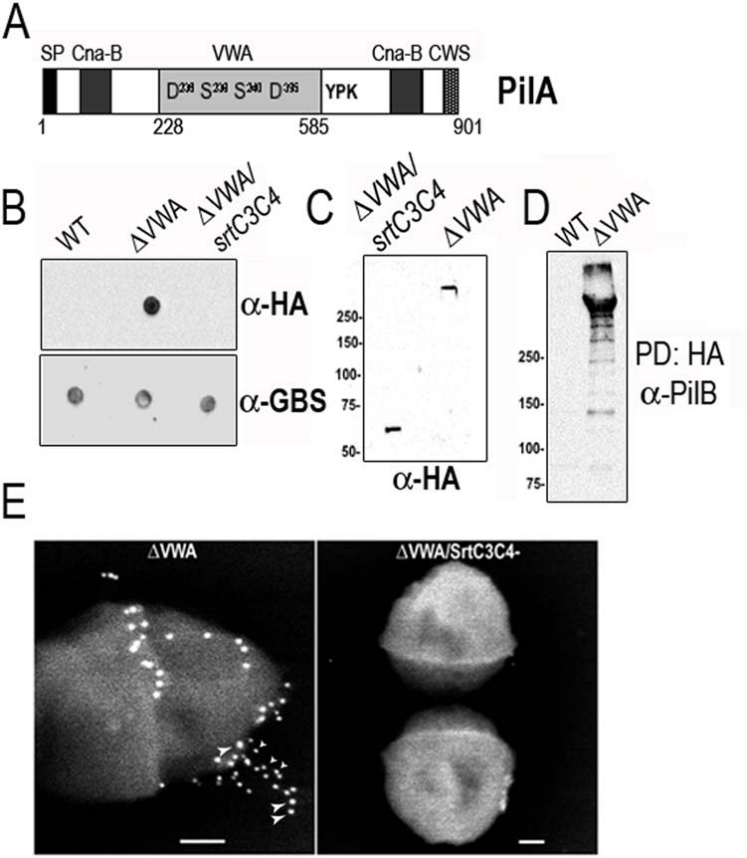
Characterization of the von Willebrand Adhesion Domain of PilA. (A) PilA contains a N-terminal signal peptide (SP), a von Willebrand factor type A domain (VWA) flanked by two Cna-B type domain, a putative pilin motif (YPK) and a C-terminal cell wall sorting signal (CWS). The critical seryl and aspartatyl residues known to interact with divalent cations in extracellular eukaryotic proteins containing a von Willebrand factor type A domain are conserved (D^236^, S^238^, S^240^, D^395^). (B) Analysis of HA display at the bacterial surface using dot-blot on whole bacteria. In the ΔVWA deletion mutant, the VWA domain has been replaced by the HA (hemagglutinin) epitope. (C) Western blot analysis of cell wall extracts of *S. agalactiae* strains. Proteins were separated on 4–12% gradient Criterion XT SDS-PAGE gel and detected by immunoblotting with specific anti-HA antiserum (Roche). (D) Pull-down experiment with HA agarose beads and immunoblotting with anti-PilB antibody. (E) Immunolocalization of the HA epitope in the pilus of *S. agalactiae* ΔVWA strain. Double-labeling experiment were performed on the parental ΔVWA strain and its isogenic ΔVWA/SrtC3C4^−^ mutant strain with rabbit anti PilB /IgG-10 nm gold (thin arrowheads) followed by mouse anti HA/IgG-20 nm gold beads (thick arrowheads) viewed by SEM.

*In silico* analysis of GBS PilA adhesin revealed the presence of a von Willebrand factor type A domain (VWA found at amino acids 228 to 585) located upstream from the putative pilin motif (YPK). This VWA domain is flanked by two Cna-B type domain found in a *S. aureus* collagen-binding surface protein (Figure 5A). However, the Cna-B regions do not mediate collagen binding but forms a stalk that presents the ligand binding domain away from the bacterial surface [38]. VWA domains in extracellular eukaryotic proteins mediate adhesion via metal ion-dependent adhesion sites (MIDAS). Binding of Mn^2+^ and Mg^2+^ to the MIDAS region in eukaryotic proteins have been demonstrated by crystallographic structures. Divalent cations were shown to stabilize the α1β1 integrin I domain [39]. Of note, the critical serine and aspartate residues known to interact with divalent cations are conserved in the VWA domain of PilA (Figure 5A). Many homologues have been identified in bacterial genomes but their role have not been characterized [40]. Multiple sequence alignments of prokaryotic and eukaryotic VWA-domains is shown in Figure S4.

We sought to determine whether the VWA domain of PilA was involved in PilA-mediated adherence. To test this hypothesis, we constructed a PilA mutant named ΔVWA in which the first 180 amino acids of the 358 amino acids VWA domain was replaced by a 9-aa residue-long hemagglutinin epitope tag (HA tag) allowing the detection of the mutant protein with specific anti-HA monoclonal antibody (Figure S4). Of note, the putative pilin motif YPK of PilA allowing its incorporation into the pilus fiber is located behind the VWA domain and left intact in the mutant (Figure 5A). This new isogenic *pilAΔVWA* mutant displayed similar growth caracteristics in Todd-Hewitt broth compared to the parental strain at 37°C (data not shown). Dot-blot analysis on whole bacteria using a commercial anti-HA antibody showed that the HA epitope is located at the bacterial surface of the ΔVWA HA-expressing bacteria. No signal could be detected in the parental strain confirming the specificity of the HA antibody (Figure 5B). Interestingly, introduction of the *srtC3C4* mutation in the ΔVWA strain to abrogate pilus polymerization caused the disappearance of the HA signal (Figure 5B). This result strongly suggests that HA detection on the bacterial surface of ΔVWA mutant depends on its incorporation into the pilus fiber. Western-blot analysis of cell wall extracts from isogenic mutants showed the presence of the HA-tagged PilAΔVWA protein in the pilus polymers of the ΔVWA strain but this incorporation is abolished in the ΔVWA/SrtC3C4^−^ mutant where a single 60-kDa protein, i.e. the predicted size of monomeric PilAΔVWA protein, is present (Figure 5C). We also analyzed the interaction between HA-tagged PilA mutant and the major pilin subunit PilB using pull-down experiment with HA agarose beads and immunoblotting with anti-PilB antibody. As shown in Figure 5D, the HA-tagged PilAΔVWA physically interact with PilB polymer in cell wall extracts. No signal was detected with wild-type extracts as control for HA specificity (data not shown). Immunolocalization of the PilAΔVWA-HA protein in the pilus fiber was demonstrated by scanning electron microscopy (Figure 5E). Double-labeling experiments were performed on the parental ΔVWA and its isogenic SrtC3C4^−^ mutant using rabbit anti-PilB polyclonal antibody followed by 10 nm gold-labeled IgG (thin arrows) and then with rat anti-HA monoclonal antibody followed by 20 nm gold-labeled IgG (arrow heads). HA staining was detected at various locations including the base and the tip (Figure 5E) and was similar to that of PilA staining (Figure 3A and [22],[27]). No staining was detected in the absence of pilus polymerization in the ΔVWA/SrtC3C4^−^ double mutant (Figure 5E). Altogether these results indicate that the PilAΔVWA protein is produced, folded, and incorporated into the pilus fiber like the intact PilA protein.

**Figure 6:**
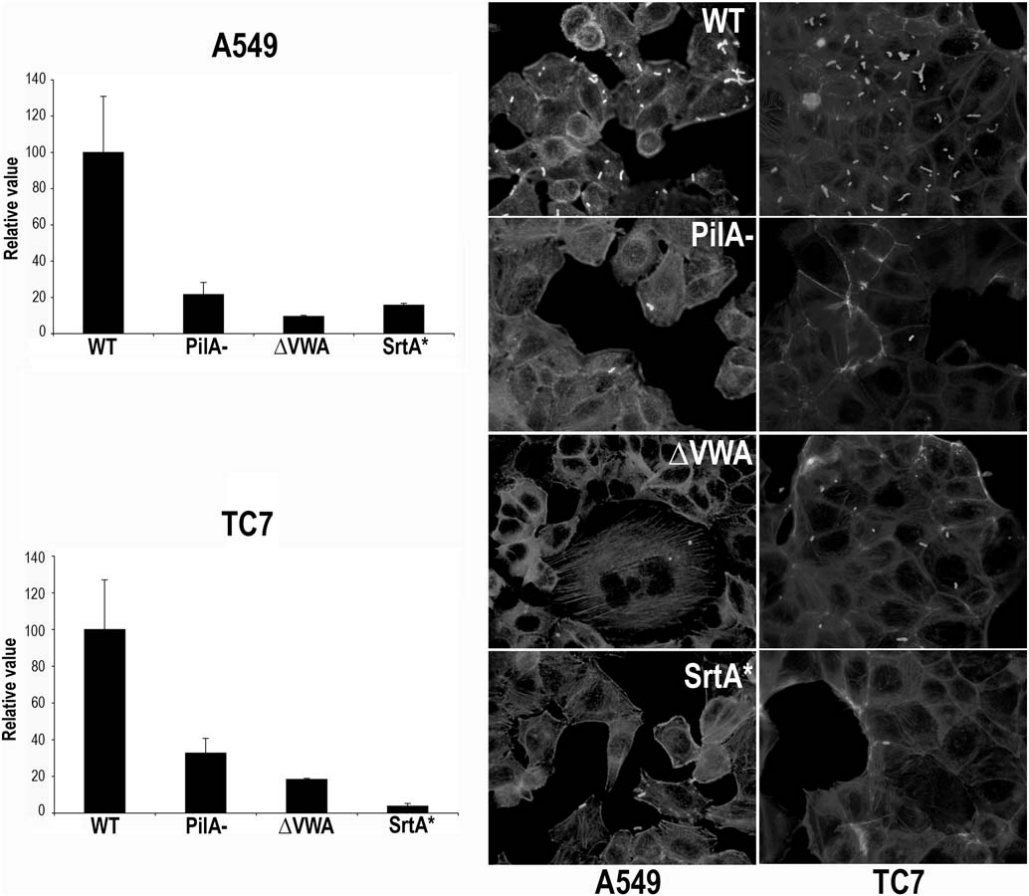
Adherence of *S. agalactiae* pilus mutants to human pulmonary epithelial cells A549 and to human intestinal epithelial cells TC7. Cells were infected at a M.O.I of 20 bacteria per cell for 1 h at 37°C and adherence frequencies were calculated from the numbers of bacteria remaining attached to the cells after the incubation period with respect to the number of inoculated bacteria. The level of adherence of the WT strain is arbitrarily reported as 100 and the level of adherence of the various mutants are relative values. The results are presented as mean value (±SD) from one representative experiment of at least 3 independent experiments (left panel). Immunofluorescence analysis of GBS adherence to A549 and TC7. Bacteria were revealed with specific rabbit anti-GBS polyclonal antibodies and anti-rabbit IgG coupled to Alexa 488 respectively. Cellular F-actin was visualized with phalloidin coupled to Alexa 594 and nuclei were stained with DAPI (right panel).

Finally, we examined the ability of the PilAΔVWA mutant to bind to human epithelial cells from alveolar (A549) and intestinal (TC7) origins. Standard adhesion assays showed that the ΔVWA mutant is strongly reduced for adherence to both A549 and TC7 cell lines compared with the parental strain NEM316, to a level similar to that obtained with the *pilA* and the SrtA* mutants (Figure 6). Collectively, these results show that the VWA domain of PilA is essential for PilA adhesive property.

### The PI-2A pilus is involved in biofilm formation

**Figure 7:**
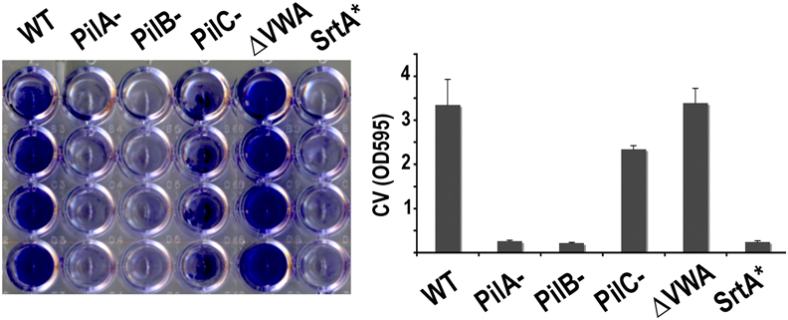
Role of the PI-2A pilus in biofilm formation. *S. agalactiae* strains were grown in 96 wells polystyrene plates in LB supplemented with 1% glucose at 37°C for 24 h. Adherent bacteria were stained with crystal violet (CV) and quantification was performed by measuring the absorbance at 595 nm. Results are representative of three experiments. Error bars show standard deviations.

We initially observed that all pilus mutant strains remained in suspension after an overnight culture in Todd-Hewitt broth whereas strain NEM316 sedimented at the bottom of the tube (data not shown). This result suggested a role of the PI-2A pilus locus in bacterial aggregation and possibly in biofilm formation. We thus began to assay the ability of *S. agalactiae* to form biofilms on microtiter polystyrene plate as previously described [41]. In this assay, staining with 0.1% crystal violet (CV) for 15 min enables the visualization of attached, sessile cells after bacterial biofilms have formed in microtiter plate wells. Biofilm assays were carried out under various conditions to determine the optimum experimental conditions. Various media (TH, THY, BHI, LB, RPMI 1640), temperature (30°C and 37°C), and time points (24 to 48 h) were used in preliminary experiments but only LB and RPMI 1640 media supplemented with 1% glucose at 37°C for 24 h produced uniform biofilms (data not shown). In enriched media such as TH, THY, BHI bacteria grew better than in LB or RPMI media but failed to evenly adhere over the surface, instead forming pellets at the bottom of the well. It appears that a nutritionally rich environment does not favor *S. agalactiae* biofilm formation on polystyrene but that nutritionally limited environment increases sessile growth. We also compared the ability of *S. agalactiae* to form biofilms on different surfaces. Polystyrene surface was more suited than polyvinylchloride or glass surfaces on which *S. agalactiae* adhered poorly. Thus, the optimal conditions to see biofilm formation with strain NEM316 were as follows: overnight culture in Todd-Hewitt medium, dilution in LB medium supplemented with 1% glucose to obtain an initial OD 600 nm of 0.05, inoculation of sterile polystyrene 96-well plate and growth at 37°C for 24 h. Biofilm formation of *S. agalactiae* strain NEM316 and its isogenic pilus mutants were assayed accordingly (Figure 7). We hypothesized that the sortase A mutants, unable to attach to the polystyrene surface, would be defective for biofilm formation as recently reported for *S. gordonii srtA* mutant [42]. Indeed, both *S. agalactiae srtA^−^* and srtA* mutants were unable to form biofilm (Figure 7). We showed that *pilA* and *pilB* mutants were as strongly impaired as the *srtA* mutants for biofilm formation. The *pilC* mutant that still forms pili was only slighly reduced for biofilm formation. Surprisingly, the *pilAΔVWA* mutant readily forms thicker biofilm, as compared to the parental strain NEM316, although it is unable to adhere to epithelial cells (Figure 6 and Figure 7).

## Discussion

Our previous functional characterization of the pilus locus in *S. agalactiae* [22] raised two major questions that were addressed in the present work: i) what is the role of the housekeeping class A sortase (SrtA) in pilus biosynthesis and ii) what is the function of the pilus fiber itself. Indeed, understanding the apparent paradox of a pilus carrying the adhesive property but yet dispensable for adherence remains a major challenge of the field.

We previously observed a down-regulation in transcription of the pilus genes in the *srtA* ^−^ mutant and therefore could not test the role of SrtA in pilus biogenesis [22]. In this report, a new *srtA* mutant (srtA*) displaying all characteristics of the *srtA* ^−^ mutant but expressing wild-type levels of PI-2A pilus was constructed. Since high molecular weight polymers of pili were seen in the srtA* mutant, it is clear that the housekeeping sortase A is not involved in the polymerization process. This is in direct contrast to the effects of deleting both pilus-associated sortases SrtC that abrogates the formation of pilus polymers (Figure 2). Shedding of pilus polymers in the culture medium of the srtA* mutant demonstrate a role of the housekeeping sortase A in the anchoring phase. A similar result was very recently reported in *S. agalactiae* strain 515 [43]. As previously shown in *C. diphtheriae* [44] and *B. cereus* [21], these results support a two-stage model of pilus assembly where pilins are first polymerized by a pilus-specific sortase and the resulting fiber is then attached to the cell wall by the housekeeping sortase. In contrast, SrtA is dispensable for pilus assembly and localization to the cell wall in *S. pneumoniae* [45]. Interestingly, the three pneumococcal RrgA, RrgB, and RrgC proteins that assemble into the pilus each have a motif (YPRTG, IPQTG, and VPDTG respectively) that is divergent in the first amino acid position of the canonical LPxTG cell wall signature sequence (CWSS) recognized by the house-keeping sortase A which could account for differences in sortase specificity.

*S. agalactiae* is a capsulated bacteria and the size of the capsule is subject to phase variation [46]. By immunogold labeling, we visualized the capsule by electron microscopy and showed that the pilus extends beyond the capsule and thus serve as carrier for surface located adhesive clusters of PilA. Thus, pili-associated adhesins, as opposed to those directly linked to the peptidoglycan, can overcome masking by the capsule as demonstrated by immunodetection of PilA on capsulated bacterial surface (Figure 3B). In a capsulated strain, PilA is not detected in the absence of PilB or in the *srtC3C4* ^−^ mutant in which pilus polymerization is abrogated (Figure 3B, data not shown). These results indicate that the pilus structure is necessary for optimal display at the bacterial surface of the PilA subunit that is necessary for adherence to epithelial cells. However, the fact that PilA remains a functional adhesin in the absence of a pilus fiber raises question on the role of this appendage. A similar situation was recently reported in *Streptococcus pneumoniae* in which RrgA, a minor pilus component, is central in pilus-mediated adherence and disease, even in the absence of polymeric pilus production [29]. As mentioned in this work, it is conceivable that the conventional *in vitro* adherence assays carried out with immortalized cells culture are not adapted to test the functional benefit provided by a pilus fiber. This was recently demonstrated for the pili of *S. pyogenes* that mediate specific adhesion to human tonsil and skin epithelial cells [47]. The authors showed that pili were not required for *S. pyogenes* adhesion to immortalized HEp-2 and A549 cell lines but were indispensable for adhesion to *ex vivo* tissues and primary human keratinocytes highlighting an important limitation of the currently used adhesion models. We reasoned that surface display of PilA adhesin could be important in more stringent conditions, as for example in the presence of liquid flow mimicking the mucociliary movement in the lung. A major limitation of the standard adhesion model is that it neglects the local fluid mechanic environment encountered in the organism. Using a laminar flow chamber system optimized to study the adhesion of *Neisseria meningitidis* under low shear stress conditions [37], we were able to prove the benefit of the *S. agalactiae* pilus fiber for adherence to human pulmonary epithelial cells A549 (Figure 4B), thus emphasizing the need of employing models that are more relevant to the infectious process when studying bacterial-host interactions.

Closer examination of EM micrographs shows a large heterogeneity in pilus structures in the wild-type strain. The composition, the size, but also the diameter of the individual pili appears highly variable and, as described in *S. pneumoniae*, bundles of individual pili could be also observed (data not shown). In agreement with our previous results [22], pili are still formed in the *pilA* ^−^ mutant but were longer than those produced by the wild-type strain. This is most probably due to the increased transcription of *pilB* in the *pilA* ^−^ mutant where a 3-fold increase in *pilB* expression was measured by qRT-PCR [22]. Synthesis of longer pili by mutants overexpressing the major pilin subunit has been demonstrated in *C. diphtheriae* [48]. Strikingly, we also observed longer and largely extended pili in the *pilC* ^−^ mutant and a higher amount of PilB polymers were found shed in the culture medium of this mutant in agreement with a role of PilC as the pilus anchor [43]. Again, similar results were obtained recently in *C. diphtheriae* [49], a bacterium where the prototype pilus contains a major pilin (SpaA), a tip pilin (SpaC), and a minor pilin (SpaB). Immunoelectron microscopy revealed that when SpaB was absent, the SpaA fibers found in the culture medium and on the bacterial envelope are considerably longer than in the wild-type strain. Incorporation of the SpaB minor pilin in the shaft base serves as the terminal step in pilus polymerization and triggers the concomitant cell wall linkage by sortase A [49].

The von Willebrand Adhesion Domain (VWA) has been identified in several prokaryotic proteins but their function remain unknown [40]. We showed here that the VWA domain of PilA is essential for its adhesive function. *S. agalactiae* strain NEM316 possesses another pilus locus (PI-1) that is not expressed [22] but displayed a genetic organization similar to that of the PI-2A locus. In particular, the putative pilus associated adhesin (Gbs0632) also contains a central VWA domain surrounded by two Cna-B domains and, interestingly, this central domain structure was also found in the pneumococcal pilus-associated adhesin RrgA [29] and in the minor pilin SpaC of *C. diphtheriae* [50]. Extracellular matrix proteins constitute good ligand candidates for these adhesins and it was recently shown that RrgA interacts with human fibronectin, collagen I, and laminin [51]. However, sequence comparisons revealed that the VWA domain of RrgA shares 59% identity with that of Gbs0632 but only 37% with that of PilA, suggesting that RrgA and PilA have different VWA-binding ligands. In agreement with this idea of different receptor recognized by different VWA domain, SpaC was shown to promote specific adhesion to human pharyngeal cell line D562 [50].

Finally, we investigated the possibility that GBS pili could also play a role in bacterial-bacterial interactions, as shown for *E. faecalis*, *S. pyogenes*, and *S. pneumoniae* [26],[31]. We demonstrated that all three individual pilus NEM316 mutants were impaired for bacterial aggregation in liquid culture. Importantly, *S. agalactiae* strain NEM316 was able to form biofilm in microtiter plates under certain culture conditions. We demonstrated that pili are key surface structures involved in biofilm formation and showed that both PilB and PilA, but not PilC, are essential in this process. Surprisingly, the VWA domain required for adherence to epithelial cells was found to be dispensable for biofilm formation on polystyrene plates. This result indicates that the VWA domain is not required for adherence to abiotic surfaces and suggests that it recognizes specific ligand on epithelial cells. These results revealed GBS pili possess dual and non-overlapping functions in participating in biofilm formation and adherence to host cells. Current work aiming at identifying the epithelial receptor of PilA is in progress. Establishing a link between biofilm formation and colonization is the next challenging question requiring the development of an appropriate animal model.

## Materials and Methods

### Bacterial strains, plasmids, and growth conditions

*S. agalactiae* NEM316 was responsible for a fatal septicemia and belongs to the capsular serotype III. The complete genome sequence of this strain has been determined [52]. *Escherichia coli* DH5α (Gibco-BRL) was used for cloning experiments. *S. agalactiae* was cultured in Todd-Hewitt (TH) broth or agar (Difco Laboratories, Detroit, MI) and *E. coli* in Luria-Bertani (LB) medium. Unless otherwise specified, antibiotics were used at the following concentrations: for *E. coli* - ampicillin, 100 µg/ml; erythromycin, 150 µg/ml; for *S. agalactiae* - erythromycin, 10 µg/ml; kanamycin, 1,000 µg/ml. *S. agalactiae* liquid cultures were grown at 37°C in standing filled flasks.

### General DNA techniques

Standard recombinant techniques were used for nucleic acid cloning and restriction analysis [53]. Plasmid DNA from *E. coli* was prepared by rapid alkaline lysis using the Qiaprep Spin Miniprep kit (Qiagen). Genomic DNA from *S. agalactiae* was prepared using the DNeasy Blood and Tissue kit (Qiagen). PCR was carried out with Ampli Taq Gold polymerase as described by the manufacturer (Applied Biosystem). Amplification products were purified on Sephadex S-400 columns (Pharmacia) and sequenced with an ABI 310 automated DNA sequencer, using the ABI PRISM dye terminator cycle sequencing kit (Applied Biosystems).

### Construction of *S. agalactiae* mutants

In-frame replacement of the VWA by an HA tag domain in *pilA* (*gbs1478*) (O1–O2; O3–O4) and modification of the catalytic sequence signature of sortase A *gbs0949* (O5–O6; O7–O8) were constructed by using splicing-by-overlap-extension PCR as previously described [22]. Mutants were confirmed by PCR and sequence analysis.

The sequences (5′ to 3′) of the primers were: O1, ACCAATGAATTCGGGGAAAGTACCGTACCG; O2,GGCGTAGTCGGGGACGTCGTAGGGGTACGGCTTTTGTTTGTCCACTGGTTTTAC; O3, TACCCCTACGACGTCCCCGACTACGCCTTGGGTGCATCATATGAAAGCCAATTTGAA; O4, GGATGAGGATCCTATCGGGGTATAATACTCAGG; O5, TAAACGAATTCGCAATGCTTTCATAGC; O6, GGCACGCCCGGGTGCTGCCGCAGTGAGTTGGCTCTTGCCAGGTGT; O7, GCGGCAGCACCCGGGCGTGCCGAAGCCACAGAACGTATTATTGTG; and O8, TCTTGGATCCAGTATAGTCATCGTAACGAATAGGC.

### Cell culture and adherence assays

The human cell lines A549 (ATCC CCL-185) from an alveolar epithelial carcinoma and TC7 clone [54] established from the parental colon adenocarcinoma Caco-2, were cultured in Quantum 286 Medium (PAA). Cells were incubated in 10% CO_2_ at 37°C and were seeded at a density of 2 to 5×10^5^ cells per well in 24-well tissue culture plates. Monolayers were used after 24–48 h of incubation.

Bacterial cultures from overnight cultures OD_600_ of 2 (approximately 6 10^8^ CFU/ml) were washed once in PBS and resuspended in DMEM. Cells were infected at a multiplicity of infection (M.O.I) of 10 bacteria per cell for 1 h at 37°C in 10% CO_2_. The monolayers were then washed four to five times with PBS, and the cells were disrupted by the addition of 1 ml sterile deionized ice-cold water and repeated pipeting. Serial dilutions of the lysate were plated onto TH agar for count of viable bacteria. The percent of adherence was calculated as follows: (CFU on plate count/CFU in original inoculum)×100. Assays were performed in triplicate and were repeated at least three times.

### Laminar flow chamber experiments

Adhesion under flow was performed as previously described [37]. Before the assay, bacteria were grown overnight in TH broth at 37°C, resuspended at OD_600_ = 0.3, and labeled for 30 min with the fluorescent marker CMFDA (Molecular Probes) at 20 µM on ice. After several washes in PBS, fluorescent bacteria were resuspended in DMEM supplemented with 10% FBS. A549 cells grown on glass slides were placed in the parallel plate flow chamber (3.3 cm×0.6 cm×250 µm, Immunetics, MA, USA) and sealed with vacuum. About 3×10^7^ fluorescent bacteria were introduced in the laminar flow chamber containing the cells at 0.04 dynes/cm2. Experiments were performed in DMEM supplemented with 2% serum and maintained at 37°C with a heated platform (Minitub, Germany). Medium was introduced into the chamber using a syringe pump (Vial Medical, Becton-Dickinson or Harvard Apparatus). Adhesion of bacteria was recorded using an Olympus CKX41 inverted microscope with a 20× objective, a Hamamatsu ORCA285 CCD camera and the Openlab darkroom software (Improvision, UK).

### Protein solubility in hot SDS

Cell-wall anchored proteins are insoluble in hot SDS unless the peptidoglycan had been first digested enzymatically with mutanolysin. In contrast, membrane anchored proteins are generally extractable in hot SDS without any prior treatment. The assay described by Garandeau *et al.* [55] was used to study the solubility of PilB polymers in NEM316 and sortases derivatives. The bacteria in 10 ml overnight culture were collected by centrifugation (6,000 rpm, 4°C, 10 min). Medium corresponds to the supernatant that was filter-sterilized and concentrated 10× by ultra filtration on Sartorius vivaspin 20 devices (cut-off 10 kDa). The bacterial pellet was washed in phosphate-buffered saline (PBS), centrifuged, and resuspended in 500 µl of 4% SDS - 0.5 M Tris-HCl pH 8. The bacterial suspension was boiled for 10 min and then centrifuged at 10,000 rpm for 5 min. Membrane correspond to the SDS-extracted supernatant and cell-wall to the pellet. These different protein fractions were further analyzed by immunoblotting.

### Immunoblotting and immunofluorescence analyses

For dot-blot analysis on whole bacteria, late-exponentially growing bacteria were washed in PBS and resuspended in adjusted volumes of PBS to get similar OD_600_ values. The bacteria were loaded on nitrocellulose membrane, dried up for 20 min at room temperature, and then blocked in PBS-milk 5% for 30 min. PilB was detected using a specific rabbit polyclonal antibody obtained previously [22] at 1∶2000 dilution and the HA epitope was detected using the rat monoclonal antibody (3F10) from Roche at 1∶1000 dilution. The secondary horseradish peroxidase (HRP)-coupled anti-rabbit secondary antibody (Zymed) was used at 1∶20000 dilution whereas the goat anti-mouse antibody was used at 1∶10000 dilution. Detection was performed using the Western pico chemiluminescence kit (Pierce). Image capture and analysis were done on GeneGnome imaging system (Syngene). For Western blotting analysis, proteins were boiled in Laemmli sample buffer, resolved on Tris-Glycine Criterion XT gradient gels 4–12% SDS-PAGE gels and transferred to nitrocellulose membrane (Hybond-C, Amersham). Protein detection was performed as described above.

Immunofluorescence staining of R28/Alp2 and PilB was performed as described [56] using specific rabbit polyclonal antibodies revealed with an anti-IgG coupled to Alexa 488 (Molecular Probes, OR). Microscopic observations were done on a Nikon Eclipse E600 and images acquired with a Nikon Digital Camera DXM1200F.

### Pull-down experiment

Bacteria (50 ml) were grown in TH medium at 37°C for 18 hours and harvested for preparation of cell wall extracts. Bacteria were washed once in PBS and resuspended in the mutanolysin digestion buffer to get an OD600 of 100 ml^−1^ (50 mM Tris-HCl pH 7.3, 20% sucrose and protease inhibitor cocktail (Roche)). Mutanolysin (Sigma) dissolved to 5000 U ml^−1^ in potassium buffer (10 mM pH 6.2) was then added to the bacterial suspension to give a final concentration of 200 U ml^−1^. The digestion was performed for 2 h at 37°C under gentle rotation. After centrifuging at 12 000 g for 15 min at 4°C, supernatants corresponding to the cell wall fractions were transferred to clean tubes. 25 µl of EZview Red anti-HA affinity gel (EZview, Sigma) was added and the samples were rotated overnight at 4°C. Beads were washed five times in solubilization buffer (20 mM Tris-HCl, 137 mM NaCl, 0,25% NonidetP40, 1.5 mM MagCl2, 1 mM EDTA, 10 mM NaF) and resuspended in 20 µl of 2× reducing sample buffer followed by boiling for 5 minutes. Samples were then analyzed by Western blot analysis.

### Immunogold electron microscopy

For scanning electron microscopy analysis, bacteria were applied to polylysine coated glass coverslips, and fixed with 0.1% glutaraldehyde/4% paraformaldehyde in 0.1 M Sorensen buffer (pH 7.2) for 30 min. Fixed bacteria were incubated in PBS supplemented with 0.25% NH_4_Cl for 20 min then washed extensively with PBS. Samples were incubated in incubated in PBS/BSA 1% for 10 min. Following incubation for 30 min with the primary antibody, samples were washed and incubated for 10 min with the secondary antibody conjugated to colloidal gold. Preparations were washed with PBS and fixed in 2.5% glutaraldehyde in 0.1 M cacodylate buffer (pH 7.2) overnight at 4°C, then washed three times for 5 min (each time) in 0.2 M cacodylate buffer, post-fixed for 1 h in 1% osmium in 0.2 M cacodylate buffer and rinsed with distilled water. Bacteria were dehydrated through a graded series of ethanol (25, 50, 75, 95 and 100%) followed by critical point drying with CO_2_. Dried specimens were sputter coated twice with carbon, with a with a GUN ionic evaporator PEC 682 and were examined and photographed with a JEOL JSM 6700F field emission scanning electron microscope operating at 5 kV. Images were acquired from the YAG BSE detector. For transmission electron microscopy, samples were processed as above. After dehydratation in ethanol the samples were embedded in epoxy resina and 70 nm thin sections were prepared and examined using a JEOL JSM1010 microscope operating at 80 kV.

For double and triple labeling experiments, the same procedure was applied using the following antibodies: the mouse monoclonal S9 anti-type III capsule (1/5), the rat monoclonal anti HA antibody (clone 3F9 from Roche at 1/100), the rabbit polyclonal α−PilB (1/100) and the rabbit polyclonal α−PilA (1/10). The secondary antibodies were goat anti mouse or goat anti rabbit conjugated to 20 nm-, 10 nm- or 5 nm gold beads.

### Biofilm formation assays

Bacterial attachment and surface growth on polystyrene microtiter plates were studied during growth of *S. agalactiae* in LB medium supplemented with 1% glucose. Overnight cultures grown in TH were used to inoculate LB glucose medium at OD600 0.1, were vortexed briefly and 180 µl volumes were dispensed into 96-wells plate (Costar 3799; Corning, Inc., NY) followed by incubation at 37°C for 24 h. The OD_600_ of each culture was measured to ensure that all cells had reached stationary phase with a similar OD_600_, and the wells were washed twice in PBS and air-dried for 15 min. Biofilms were stained with 0.1% crystal violet for 30 min (100 µl per well) and the wells were washed twice with PBS and air-dried. The stained biomass was resuspended for quantification in ethanol/acetone (80∶20) and A_595_ was measured. The assay was performed in quadruplet.

## Supporting Information

Figure S1 IEM analysis of the capsular type III polysaccharide. *S. agalactiae* wild-type strain NEM316 was incubated with a mouse monoclonal antibody raised against the type III capsular polysaccharide (mAb S9) and rabbit polyclonal antibody raised against PilA and PilB, and. Antibodies were conjugated to 5 nm gold particles for capsule, 10 nm for pilB and 20 nm for PilA. The outer layer of the capsule is marked by black arrows. Scale bar is shown for each panel.
(9.12 MB TIF)

Figure S2 Visualization of pili by immunofluorescence. Visualization of pili by immunofluorescence using polyclonal anti-PilB antibody. (A) on whole bacteria- (B) detached pili found in the extracellular medium.
(3.00 MB TIF)

Figure S3 Adhesion under flow conditions. A monolayer of A549 cells was cultivated and placed in a flow chamber. The same amount of fluorescently labeled strains were introduced under flow and adherent bacteria were detected by fluorescent microscopy. Representative fields are presented: A549 cells as seen by phase contrast (A) and adherence of the wild type strain NEM316 and isogenic mutant derivatives (B-F).
(8.32 MB TIF)

Figure S4 Multiple sequence aligments of procaryotic and eucaryotic VWA-domains. The alignment was generated by comparing PilA (GBS1478) to the SMART database using Profile hidden Markov models (HMMER). Computations were made online at the following URL (http://smart.embl-heidelberg.de/). Note the presence of a VWA domain in the pili adhesin subunit Gbs0632 from *Streptococcus agalactiae* (PI-1 pili operon), RggA from *Streptococcus pneumoniae*, and SpaC from *Corynebacterium diphteriae*. Explanation of codes used in CHROMA coloured alignments is as described in http://smart.embl-heidelberg.de/help/chroma.shtml.
(0.65 MB DOC)
