# Supplementary figures and images for: Dual Role for Pilus in Adherence to Epithelial Cells and Biofilm Formation in Streptococcus agalactiae
Source: PLoS Pathog. 2009 May 8;5(5):e1000422. doi: 10.1371/journal.ppat.1000422 (PMC2674936; doi:10.1371/journal.ppat.1000422)

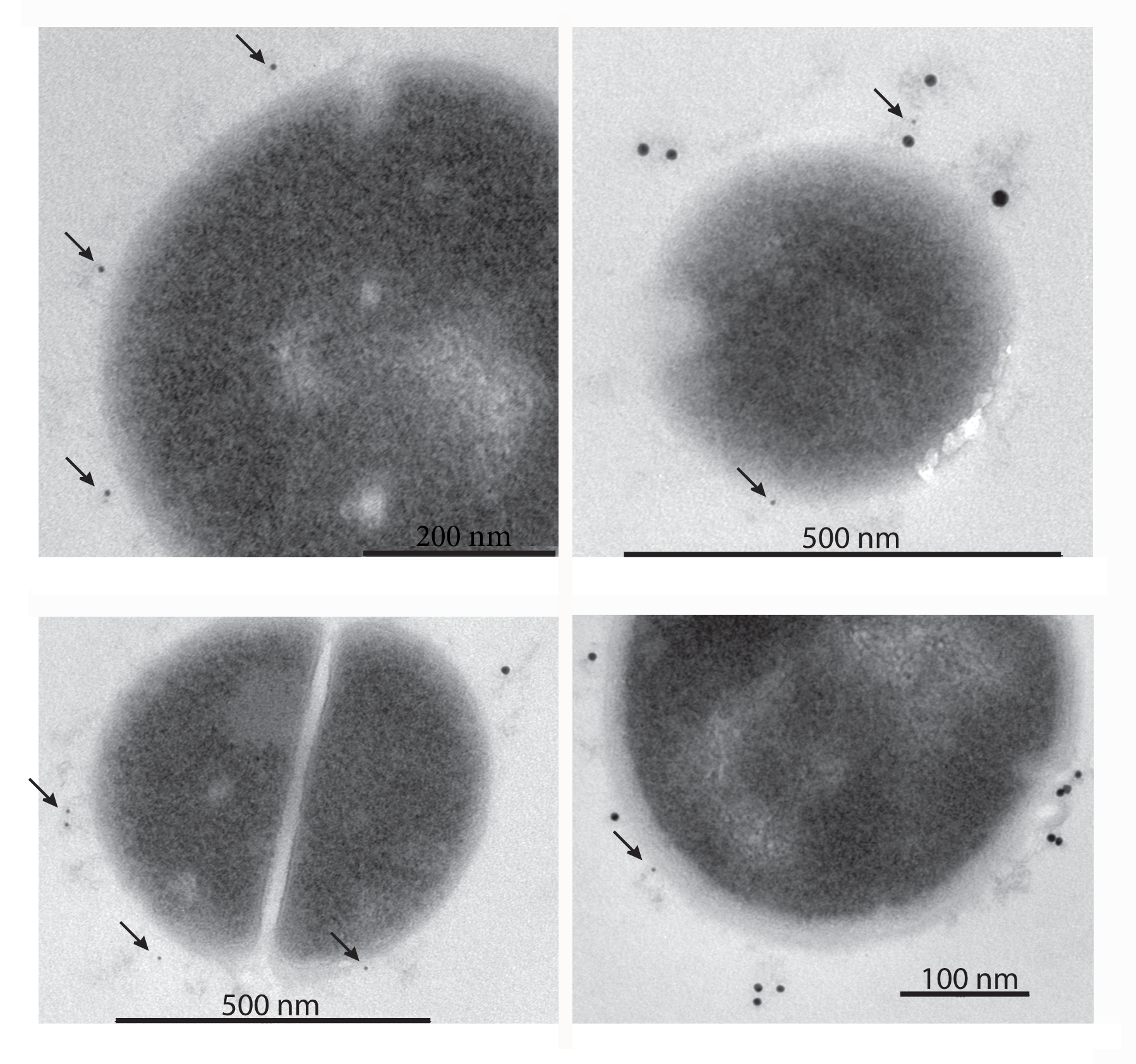

Supplement: Figure S1 — IEM analysis of the capsular type III polysaccharide. S. agalactiae wild-type strain NEM316 was incubated with a mouse monoclonal antibody raised against the type III capsular polysaccharide (mAb S9) and rabbit polyclonal antibody raised against PilA and PilB, and. Antibodies were conjugated to 5 nm gold particles for capsule, 10 nm for pilB and 20 nm for PilA. The outer layer of the capsule is marked by black arrows. Scale bar is shown for each panel. (9.12 MB TIF) [file ppat.1000422.s001.tif]

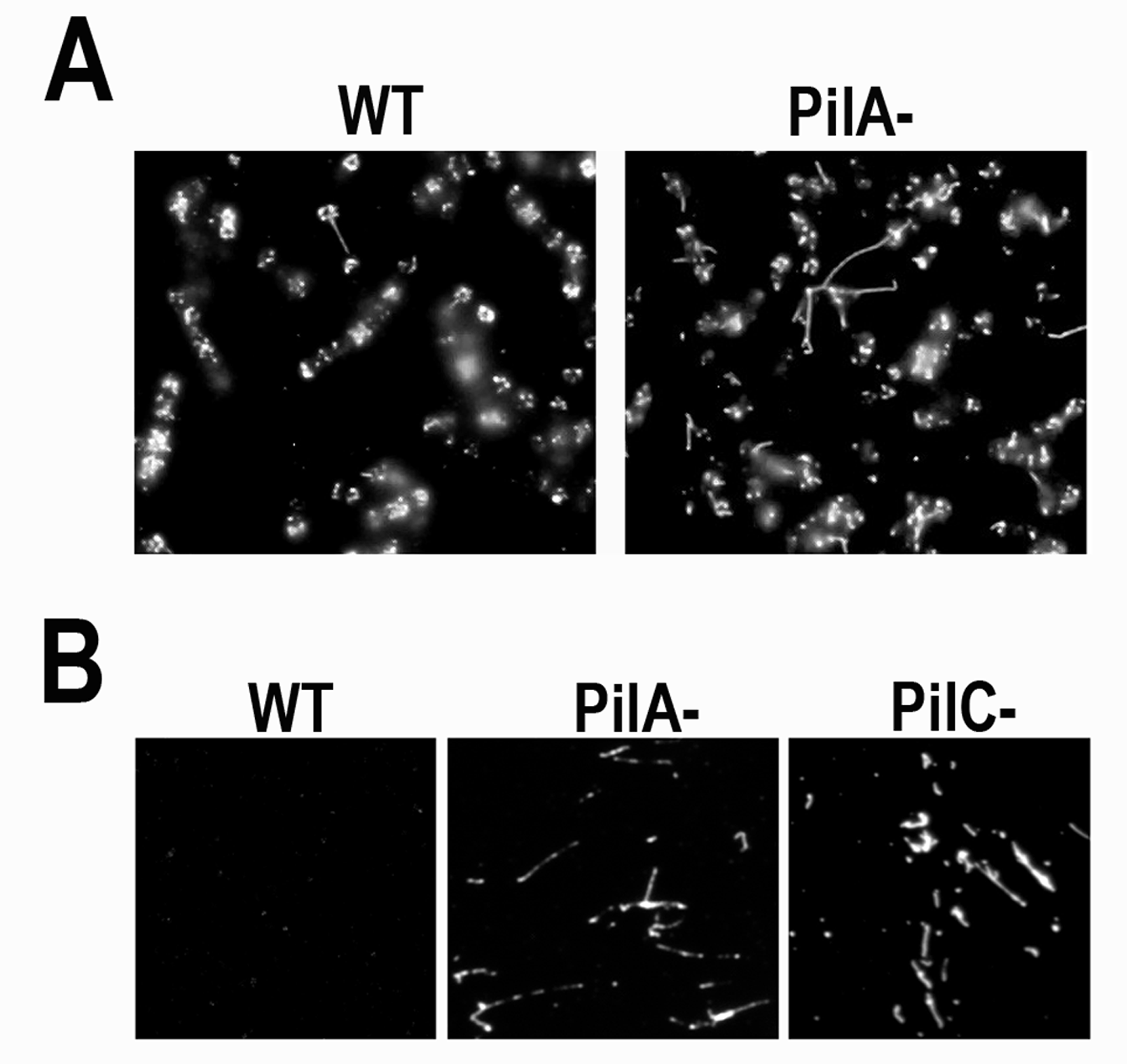

Supplement: Figure S2 — Visualization of pili by immunofluorescence. Visualization of pili by immunofluorescence using polyclonal anti-PilB antibody. (A) on whole bacteria- (B) detached pili found in the extracellular medium. (3.00 MB TIF) [file ppat.1000422.s002.tif]

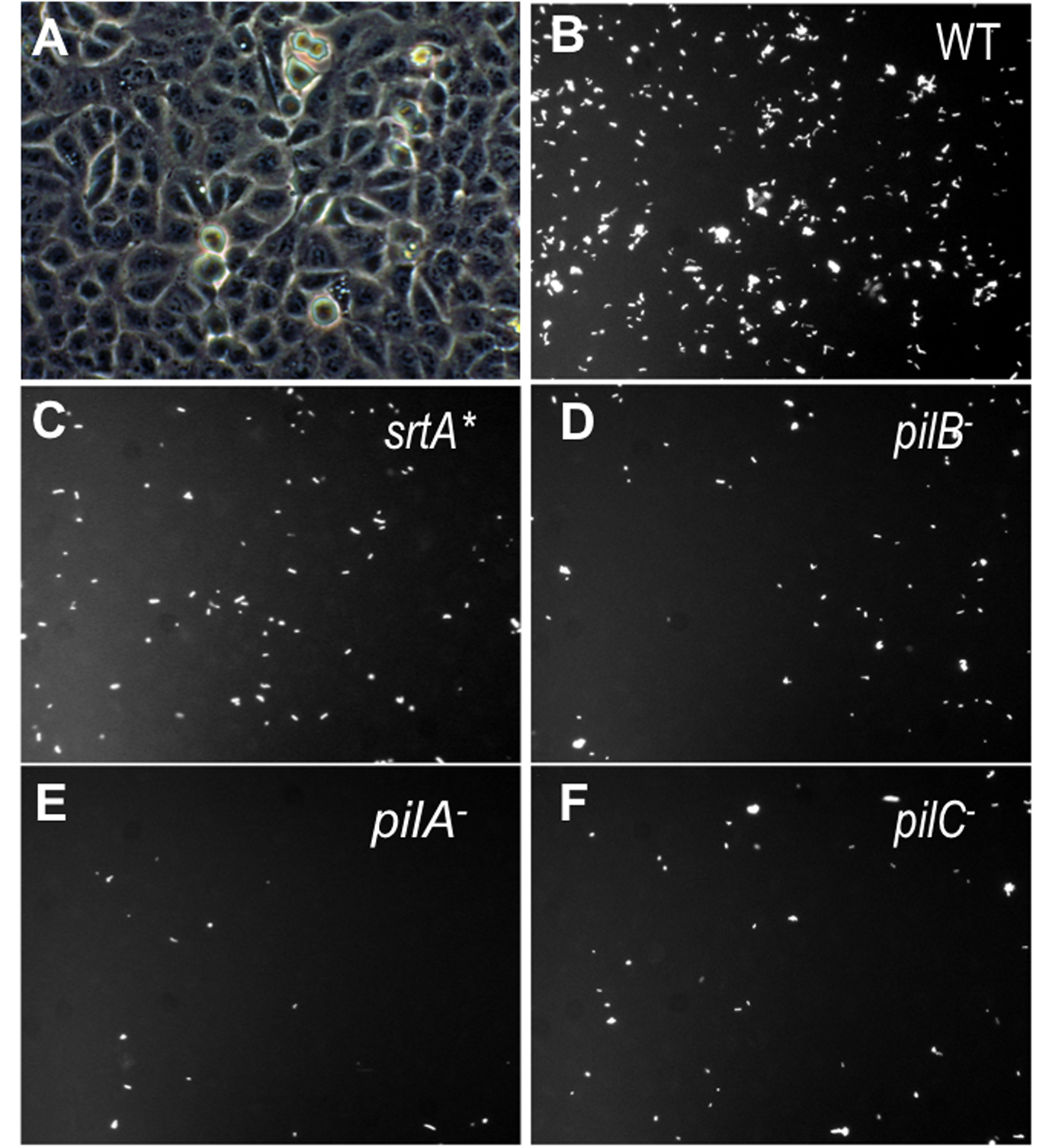

Supplement: Figure S3 — Adhesion under flow conditions. A monolayer of A549 cells was cultivated and placed in a flow chamber. The same amount of fluorescently labeled strains were introduced under flow and adherent bacteria were detected by fluorescent microscopy. Representative fields are presented: A549 cells as seen by phase contrast (A) and adherence of the wild type strain NEM316 and isogenic mutant derivatives (B-F). (8.32 MB TIF) [file ppat.1000422.s003.tif]
