# Supplementary material for: Dual Role for Pilus in Adherence to Epithelial Cells and Biofilm Formation in Streptococcus agalactiae
Source: PLoS Pathog. 2009 May 8;5(5):e1000422. doi: 10.1371/journal.ppat.1000422 (PMC2674936; doi:10.1371/journal.ppat.1000422)
Supplement: Figure S4 — Multiple sequence aligments of procaryotic and eucaryotic VWA-domains. The alignment was generated by comparing PilA (GBS1478) to the SMART database using Profile hidden Markov models (HMMER). Computations were made online at the following URL (http://smart.embl-heidelberg.de/). Note the presence of a VWA domain in the pili adhesin subunit Gbs0632 from Streptococcus agalactiae (PI-1 pili operon), RggA from Streptococcus pneumoniae, and SpaC from Corynebacterium diphteriae. Explanation of codes used in CHROMA coloured alignments is as described in http://smart.embl-heidelberg.de/help/chroma.shtml. (0.65 MB DOC) [file ppat.1000422.s004.doc]

**PilA (901 aa)**

**
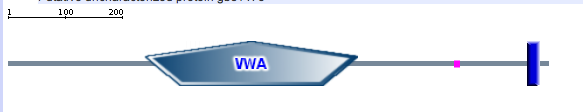
**

**Transmembrane domain**

**180-aa deletion**

PilA P**LD**.**VVFVLD**..**NS**N**SMN**NDGPNFQRH**NK**A**KKA**A**EAL**.G**T**A**VKDI**LGA.....N**SDNRVALVTYGS**(4)GR**SVDV**V**KGF**KE(21)QL**TNN**AE**EIIK**R**I**P**T**EAP(19)YLSK**VG**E**T**F**T**.M**KA**FM**EA**D**DI**....---L**S**Q**VDR**N-----.-SQ**KIIV**H**ITDG**V**PT**RS(122)EDYKK**N**Q.DG**T**F**QKLKE**E-...----**A**FE**LSDG**....EITE**L**M**KS**F**SS**K**P**EY-YTPI-.VTSSDASN**N**E**I**L**SKIQQ**QF

GBS0632 P**LD**.**VV**V**LLD**..**NS**N**SMN**NER..ANNSQ**R**AL**KA**G**EAV**.E**KLIDKIT**S......N**KDNRVALVTYAS**(7)ATV**SKG**VA**DQ**N......GKALN**DSV**SWDY**HK**T**T**F(37)TLYQF**GAT**F**T**.Q**KAL**M**KA**N**EI**....L**ET**Q**S**S**NAR**K-----.---**KLI**FH**VTDG**V**PT**MS(122)GEPTTL**Y**.**FNGN**I**R**P**K**GY**D**...**I**FTV**GIGVNGD**P(5)E**AE**K**F**M**QSISS**K**T**ENY-----.-----TNV**D**D**TNKIYDEL**N

RggA P**LD**.**VVILLD**..**NS**N**SMS**NIR..NKNAR**R**A**ERA**G**EA**T.R**SLIDKIT**S......D**S**E**NRVALVTYAS**(4)GT**E**F**TVEKG**VADK...NGK**R**LN**DSLF**WNY**DQ**T**S**F(37)LMYQF**GAT**F**T**.Q**KAL**M**KA**D**EI**....-**LT**Q**Q**ARQ**N**S-----.--Q**KVI**FH**ITDG**V**PT**MS....YPINF**NH**.A**T**F**A**P**SYQN**Q-...--**L**N**A**FFSK**SP**N..KD**GI**L**L**S**D**F**I**TQA**TS**GEHT---.--------IVRG**D**GQ**SY**QM.

SpaC G**G**S.**VAI**SF**D**..L**S**N**SLS**........A**SD**V**EKSKQA**A.L**ELVKSL**KG.......**SP**Y**R**F**GI**Y**TFAS**(5)GN**KN**F**T**PV**SL**A......ND**DGYNKV**VA**AINDIQ**-(7)KKGSP**NGGTNW**.EG**GLQ**AIA**N**D....---I**D**R**G**I**K**YDA---.-----**V**YF**ITDGQPT**WD(12)VVELE**NA**.V**T**Q**AK**L**ISD**K**G**...A**KLIPVGIG**QLS(31)T**GK**Q**MLEKI**T**S**P**G**LEPII---.LPDYST**L**P**Q**R**M**G**QQI**FT**G**C

S44454/1-179 MRH.**LY**V**VVD**..**GS**RT**M**EDQ...DLKP**NRLT**C**T**L**KLL**.EYF**VEEY**FDQ.....NPI**SQIGII**VTK**S**....K**RAE**K**L**TE**L**SG....NPR**K**H**ITSLKKAVD**MT--..-----**C**H**G**EP**SL**.Y**NSLSMAMQ**T....L**KH**MPGH**TS**R-----.---**EVLII**FSSL**TTC**--.....--DPS**NI**.**YD**LI**KTLK**AAK...**I**R**V**S**VIGLSA**-.....EVRVC**T**V**LA**RE**TG**GTYH---.---VILDE**S**H**YKELLTHH**L

O75903/1-164 ESE.**VII**C**LD**..**CS**S**SM**E........G**VTF**L**QAKEI**A.L**H**A**LS**LV**G**........**E**KQ**KV**N**IIQFGT**.....GY**K**E**LFSY**PK.....H**ITS**N**T**AAA**EFI**M**S**A**T**P..-----**T**M**GNTDF**.W**K**T**LRY**LSL**L**....-----Y**PAR**G-----.--S**R**N**ILLV**S**DGH**LQ--.....-----**D**E.SL**T**L**Q**L**VKR**SRP.HTR**LFA**C**GIGST**....A**NR**HV**LR**I**LS**QC**GA**GVF----.------EYFN**AKSKHSW**RK

Q18048_1/1-168 PS**D**.**IIFVID**..**A**TS**S**V-........R**G**I**FEQ**YI**TYI**.E**KVVE**G**LD**VQ.....P**TVDHVG**A**I**V**YSS**E...K**K**Q**RTKI**K**L**G......E**HKDRGSL**V**KAVDEL**PF..-----**FSG**I**TAT**.**GQALKFA**A**NH**....----**T**E**G**R**R**ENF---.--TLNY**VILTDG**Y**S**YD-.....--LIE**SG**.A**RV**L**REV**P**N**S-...-A**IYAVSIG**EI....FL**R**K**ELE**M**I**T**G**N**PD**N------.------**V**L**T**GSM**SYGTLV**K

Q21540/1-176 A**V**E.**VILLLD**..**AS**G**SIG**........D**DTFKK**QL**SF**A.M**HL**A**SRLN**IS.....E**DGSH**M**ALIQYA**E.....**TPKLEFSL**G......Q**FN**HP**TQLE**W**AIQRIE**Y..-----Q**SGATNT**.**GQALRLTLEK**....---GLQ**GAR**PGI---.--P**KV**A**IVITDGQS**QD-.....-----**DV**.S**EPSQ**L**LRD**A**D**...**V**M**VYAIGVTN**L....V**N**VH**QLHQ**MT**G**N**PV**RVFT---.VESFEQ**L**D**R**A**L**A**D**S**LTW**SM

O76430/1-183 ES**D**.**IVIVVD**..**AS**NA**YD**........Q**VTFEQ**I**RTFL**.L**N**F**VSSLT**VS.....PL**DSQVAL**YA**YGS**.....**SAQT**VA**SL**N......D**GSSLGSIQ**AK**INT**TLK..---YQ**GSA**ER**NL**.F**QALTKVQ**A**E**....-V**S**SMS**G**M**R**SGD---.-YK**KVLVIL**S-**GDS**WTG.....-NTVI**G**S.SVL**TQ**LK**QK**F**N**..V**I**MSV**G**F**G**AK**A**LA...NLN**SQL**P**QI**T**T**A**TT**NTF----.---FAA**T**S**D**Q**LT**YVSA**WM**A

CA36_CHICK_2/1- S**AD**.**LIFLID**..**GS**DN**IG**........S**VNFQ**AI**RDFL**.V**NLIESL**RVG.....A**Q**QI**HIGVVQYSD**.....**QPRTEFAL**N......S**YSTKADVLDAVK**A**LS**F..----R**GG**KEA**NT**.**G**A**ALEYV**V**E**N....L**FT**QAG**GSR**IEEA--.-VP**QILVLI**SG**GESS**D-.....-----**DI**.**REG**LLA**VKQ**A**S**...**I**FS**FSIGV**L**NA**.....**D**S**AELQQIAT**D**GS**FAF----.---TALDI**R**N**L**AA**L**R**ELL**L

Q22422/1-200 Q**LD**.F**A**VA**VD**..FTA**S**N**G**NP(11)GIP**NQYEIA**L**R**S**V**.L**TI**C**QHYN**........**SS**K**T**FEAYG**FGA**(4)HQ**TVSA**V**FPL**DL(7)VGING**VMN**AY**RHALQNVT**-..-----**L**Y**GPTNF**.**S**PI**I**V**EV**A**NK**....A**QK**MMK**TT**AR-----.--Y**QILLIITDG**II**S**D-.....-----M**Y**.A**T**I**NT**V**IN**AS**G**L.P**L**S**II**I**IGVGN**E.....**DF**E**R**M**HEL**D**S**D**DA**LL-----.---QQDSRIAE**RDIVQFV**T

CA16_CHICK/1-17 P**AD**.**IMLLVD**..**SS**T**S**V**G**........SK**NFD**T**TKNFV**.K**RL**A**ERF**LEASK.PAE**DSVRVSVVQYSG**R...N**Q**Q**KVE**V**PF**-.......Q**RNYT**V**I**A**KAVDN**M**E**F..-----**MN**E**ATDV**.**N**A**ALQY**I**M**G**L**....---Y**Q**R**SSR**SGA---.--K**K**K**VLV**FS**DGNS**QG-.....-ITAR**AI**.**ERTVQEVQQ**A**G**...**I**E**VYVLAVGS**Q....V**NEP**N**VR**V**L**V**T**GK**S**TNY----.-----D**V**AYGE**RHLFR**VPD

CA26_MOUSE_1/1- P**V**N.**VYFVLD**..**TS**E**S**V**A**MQ...SPTD**S**L**L**Y**H**M**QQFV**.P**Q**F**ISQL**QNE(4)QVAL**S**W**R**Y**G**G**LHFSD**.....**QVEVF**S**P**P-.......**GSDRAS**FT**K**S**LQ**G**IR**S..-----**F**RR**GT**F**T**.**DCAL**ANMT**QQ**....---I**R**QH**V**GKGV---.--V**N**FA**VVITDGHVT**GS.....--PCG**GI**.**K**MQ**AERARE**E**G**...**I**R**LFALA**P**N**R**N**....L**NE**QG**LRDIAN**S**P**HELY----.RNNYAT**M**RPDS**TEI**D**Q**D**T**I

G4056421/1-177 IRY.**LYIVID**..F**S**RAA**A**EM...DFRP**SRM**A**I**MA**KHV**.EAF**IREF**FDQ.....NPL**SQIGLVS**IK**N**....GV**AHTL**T**DL**-.......**G**G**S**PE**T**H**IKAL**MGKLE..-----**A**L**GDSSL**.Q**NALELVHEH**....---L**N**Q**VPS**YGH---.--R**EVLIL**YSAL**CTC**--.....--DPG**DI**.**MET**I**QKCKK**SK...**L**RCS**VIGLSA**-.....E**M**F**I**C**KHL**CQE**TG**G------.LYSVAVDEVH**LKDLL**L**EH**A

ITH4_PIG/1-183 PKN.**VIFVID**..**TS**G**SM**R........GR**KIQQTREAL**.I**KIL**G**DLG**........**S**R**DQ**FN**LVSFSG**.....**EA**PR**RRA**VAA.....S**AEN**VE**E**A**KSY**AA**EIH**-..-----**A**Q**GGTNI**.**NDA**ML**MA**V**QL**....L**ER**A**N**REELLPA---.RSV**T**F**IILLTDGDPT**VG.....ETNPSK**I**.**QKNVREA**I**D**GQ...HS**LFCLG**F**G**F**D**....V**PYAFLEK**M**A**LE**NG**GLARRI-.---YEDSD**S**A**LQ**L**E**DF**Y**QE

O15019/1-182 Q**Y**Q.**ICL**A**ID**..**DS**S**SMV**.......DNH**TKQLA**F**E**S**L**.AV**I**G**N**A**LT**L......L**EVGQIAV**C**SFG**E.....**SVKLLHPF**HEQ...FSDY**S**G**SQILRL**C**K**----..----FQQKK**T**K**I**.**AQ**F**LE**S**V**A**NM**....FAAA**Q**QL**SQ**N---ISSETA**QLLLVV**S**DGRG**LFL.....-EGKER**V**.**L**A**AVQ**A**ARN**A**N**...**I**F**VI**F**V**V**LDNP**SSRDSI**LDI**KVP**I**FKG**PG**E------.------**M**P**E**IR**S**Y**M**E**EF**PF

Q94677/1-177 K**VD**.**LYLLVD**..**GS**G**SIG**.......YA**N**W**ITRV**IP**ML**.TG**LIENLN**LS.....K**DS**I**NL**YMSL**FAS**.....**HTT**E**LI**R**L**GS....GPSM**DK**K**Q**A**LNVVRDLR**K..--GYEPY**GNTSM**.**SSALSEVE**M**H**....L**KD**RVNR**PN**A-----.--I**QLVIL**M**TDG**I**PN**--.....--NKYR**A**.**LE**L**SR**A**LKE**R**N**...**V**K**L**A**VIGIG**Q**G**....I**NH**Q**Y**N**K**LM**AG**CR**P**RE-----.------RS**C**K**F**Y**S**S**ADW**SE

CA36_CHICK_3/1- KK**D**.**VVFLID**..**GS**DGV-........RRG**F**P**L**L**KTFV**.E**RVVESLD**IG.....R**D**K**VRVAIVQYSN**.....AI**Q**P**EF**L**L**D......A**YEDKADL**V**SAIQ**A**LT**I..----M**GGSP**L**NT**.**G**A**ALDY**L**IK**N....V**FT**V**S**S**GSR**IAEG--.-VP**Q**F**LILLT**AD**RS**QD-.....-----**DV**.**RRPS**VV**LKT**S**G**...TVP**FGIGIGNA**.....**DLTELQTIS**FL**PD**FAIS---.VPDFSQ**L**D**S**VQ**Q**AVS**NRV**I

Q25757_1/1-185 F**YD**.**ITLVLD**..E**S**A**SIS**.......DLIW**RNEV**IP**F**S.L**EIIKRIN**IS.....Y**KNVH**M**GVL**L**FS**E.....Y**TR**D**I**VR**F**YD....NARYE**KGTLQT**K**INDLK**R..--DYRS**G**KK**T**Y**I**.I**QALRYALTY**....YS**K**L**S**NRK**E**A-----.--P**KV**TM**L**F**TDGNDP**Y-.....-ESEK**GL**.**QD**I**A**LL**YRK**E**N**...**V**K**LLVVGVSTA**.....**SENKLK**M**L**V**G**C**AP**NVVCPF-.---VIK**T**EWG**L**L**K**S**VSEV**F

S69790/1-179 G**AD**.**VM**A**LLD**..**VS**QK**MT**........KE**NFDKAKE**Q**I**.K**K**M**VTTLT**GE(6)NHN**R**R**NSV**R**L**M**TF**YR.....**KVS**DP**I**E**L**T.......**TKN**V**D**AK**LK**E**V**W**D**QAKK.----D**WD**W**G**V**DL**.QG**AIHKAREI**....-**FK**K**E**KK**SK**K-----.--R**Q**H**IVL**FSQ**GEST**FS....YDIHNK**S**D.S**K**IL**KT**RV**N**E-...----**NI**TT**SNP**.....L**FPWL**PIFNHT**N**RKA-----.-----D**M**I**D**D**VK**Y**LIKWG**E

CA1C_CHICK_3/1- K**AD**.**IVFL**T**D**..**AS**W**SIG**........D**DNFNKV**V**KFV**.F**N**T**V**GA**FD**LI....NPA**G**I**QVSLVQYSD**.....**EAQ**S**EF**K**L**N......T**FDDKAQ**A**L**G**ALQNVQ**Y..-----R**GGNT**R**T**.**GKALTF**I**KEK**....V**LT**W**E**S**G**M**R**RGV---.--P**KVLVVVTDGRS**QD-.....-----E**V**.**RKAAT**V**IQH**S**G**...FS**VFVVGVADV**.....**DYNEL**A**KIAS**K**PS**ERHVFI-.VDDFDA**F**E**K**IQ**DNLVTFV**C

Y811_METJA/1-16 K**G**P.**IIILLD**..H**S**G**SM**Y........G**DRE**I**WGK**A**V**A.L**SIIE**IAKR.......**EN**R**DI**YY**I**A**F**D**D**.....G**VRFEK**K**I**-.......NPKTI**T**FD**EIIE**IA**S**L..----Y**FGGGTNF**.IMP**LNRAMSI**....-**IK**E**H**E**T**F**K**N-----.---AD**ILLITDG**Y**A**EV-.....--NDVF**L**.**KE**F**DK**FK**NE**Y**N**...AK**LISV**F**V**E**T**F.....**P**TET**LK**A**ISD**E**V**IKV-----.---YDL**A**D**E**E**ARKIYK**S**I**S

Y103_SYNY3/1-17 P**L**N.**LCLVLD**..H**S**G**SMD**........GQP**LE**T**VKSA**A.LG**LIDRL**E........**EDDRLSVI**A**F**DH.....**RAKI**V**I**ENQ......Q**VRN**G**A**A**I**A**KAIERLK**-..-----**A**E**GGTAI**.**DEGLKLGIQE**....----AAK**GK**EDR---.--V**S**H**I**F**LLTDGEN**EHG.....--DNDR**C**.**LK**L**GT**V**ASD**YK...**L**T**VHTLG**F**GD**H....W**NQD**V**LE**A**IAA**S**A**QGS-----.-LSYIENP**S**E**A**L**H**T**FRQL**F

O88341/1-188 G**T**E.**IAIVLD**..**GS**G**SI**E........P**SDFQKAKDFI**.S**T**MM**RNF**YEK.....CFE**CN**F**ALVQYGG**.....VI**QTEFDL**L......DS**RDIN**AS**L**AK**VQSI**VQ..-----**V**KE**VT**K**T**.**ASA**M**QHVLD**N....I**F**IP**S**R**GSR**KKA---.--L**KV**M**VVLTDGD**IFR-.....--DPL**NL**.T**TV**I**SS**S**K**MQ**G**...**V**VR**FAIGVGNA**FENNN**TY**R**ELK**L**IAS**D**P**KAAHTFK-.VTNYSA**L**DGL**LSKL**Q**QRI**I

CA36_CHICK/1-18 V**AD**.**IIFLVD**..**SS**W**SIG**........KE**HFQLVREFL**.Y**DVVK**A**LD**VG.....G**ND**F**R**F**ALVQFSG**.....**NPHTEF**Q**L**N......T**Y**P**S**NQ**DVLSHI**A**N**MPY..-----**MGGGS**K**T**.**GKGLEY**L**IE**N....H**LT**KAA**GSR**ASEG--.-VP**QVIIVLTDGQS**QD-.....-----**DV**.AL**PSS**V**LKS**AH...**V**NM**IAVGV**Q**DA**.....**VEGELKEIAS**R**P**FDTHLFN-.LENFTA**L**HGI**V**G**DLV**AS**V**R

Q25757_4/1-171 V**LD**.**IA**V**VLD**..Q**S**SN**IS**.......KDQWNV**Y**I**KQFV**.I**N**T**VNQ**NYLS.....KYR**SHI**T**IVE**M**G**K.....**STK**E**KWSL**NK....KISYQ**K**K**KIIK**K**INKL**PI..----S**YS**KKK**DI**.**AKSLKYVRTK**....V**FK**K**S**E**TNR**K-----.---**KLII**M**L**VE**GKSN**S-.....--NMN**DL**.**RK**E**V**GL**LK**VN**N**...**I**DF**FA**Y**AIDN**I.....**D**G**TE**Y**K**I**LGD**CE**G**S------.--------VD**M**GL**MGN**SPP

Q05708/1-209 I**AD**.**IVILVD**..**GS**W**SIG**........RF**NFRLVRHFL**.E**NLVT**A**FD**VG.....S**E**K**TRIGV**K**N**AD**V**(8)EP**DSTH**V**YN**V-.......-AE**FD**LM**HTVVESLT**R(34)RSF**MVN**W**T**H**A**.**P**GN**VEK**Y**R**VV....-**Y**YP**T**R**GGK**PDE---.-------**VV**V**DGTVS**S-.....-----**TV**.**LKN**LM**SLTE**YQ...**I**A**VFAI**YAH**TA**S..EGL**RG**TE**TTLA**LPM**A**SD-----.--------LL**L**Y**D**V**TE**NSM

CA1E_CHICK/1-18 K**AD**.**LVFLVD**..**GS**W**SIG**........D**DNFNK**II**SFL**.Y**S**T**V**GA**LD**KI....GP**DGTQVAIIQFSD**.....**DPRTEF**K**L**N......A**YKTK**E**TLLEAIQQI**AY..-----K**GGNT**K**T**.**GKAIKHARE**V....L**FT**G**E**A**G**M**R**KGI---.--P**KVLVVITDGRS**QD-.....-----**DV**.N**KVSREMQ**LD**G**...FSF**FAIGVADA**.....**DYSEL**V**NIGS**K**PS**ERHVFF-.VDDFDA**F**T**K**IE**DELITFV**C

O86590/1-184 K**VD**.**LV**--**LD**..**VS**G**SM**RTRD..IDGG**TRM**AA**AKQA**F.N**EVLD**AT**P**........**E**E**VRLGI**R**T**L**GA**(11)K**DT**AQ**LYP**VG.......PL**DRTE**A**KTAV**A**TLS**P..------**TG**W**TPI**.**G**P**AL**L**KA**A**D**D....-----L**DG**GDGS---.---**K**R**IVLI**S**DGEDT**C-.....--APL**D**P.C**EVAREI**AAK**G**I.G**L**T**I**D**TLGLVPN**T...KM**R**Q**QLSCIA**EA**TG**GTYT---.------SV**E**H**TDELTDKV**N

O45443/1-188 W**LD**.**VVFVID**NCKIG**SM**-........NLV**YQ**TI**SSL**F.S**K**Q**LQ**IG**T**GYD...DP**RSTRVG**F**ITY**NW.....**NAT**DVA**DF**Y......K**LQSWADL**N**S**Q**IQRLQ**YT.--PQSS**SPAS**R**M**.**DTGLN**A**AI**G**M**....-**ID**A**T**A**G**F**R**D-----.NYK**KIVIV**F**T**SV**HGS**Y-.....--KSNQP.**RDVSK**I**LKS**R**G**...**I**P**V**V**TV**NT**GSS**S...D**TQAYLKQIAS**D**N**MSFA----.IADGNV**T**Q**E**I**LK**A**MTD**TNC

CA16_MOUSE_1/1- P**AD**.**ITILLD**..**SS**A**S**V**G**........SH**NFE**T**TK**V**F**A.K**RL**A**ERF**LSAGRADPS**QDVRVAVVQYSG**Q..GQ**Q**QP**GR**A**AL**Q......F**LQNYT**V**L**A**S**S**VDS**M**D**F..-----**INDATDV**.**NDALSYV**T**RF**....Y**RE**A**S**S**GAT**K-----.---**K**R**VLL**FS**DGNS**QGA.....--TAE**AI**.**EKAVQEAQR**A**G**...**I**E**IFVV**V**VGP**Q....V**NEPHIR**V**L**V**T**GK**T**AEY----.-----D**V**AFGE**RHLFR**VPN

G4009460/1-174 QRI.**VCLVLD**..K**S**G**SMA**.......TG**NRLNR**L**NQA**G.QLF**L**L**Q**T**V**E.......L**GS**W**VG**M**VTF**D**S**.....A**AHVQ**SE**L**IQI...NS**GSDRDTL**A**K**R**L**PAA--..-----**ASGGTSI**.**CSGLR**S**AFT**V....---I**R**KKYPTDG---.---**S**E**IVLLTDGEDN**--.....-----**TI**.SG**C**F**NEVKQ**S**G**...AI**IHTVALGPS**.....**A**AQ**ELEELS**KM**TG**GLQTY--.ASDQVQNNGL**ID**A**FG**A**L**SS

G2801415/1-174 S**V**N.**IAFLID**..**GS**S**S**V**G**........D**SNFRL**ML**EFV**.S**NI**A**KTF**EIS.....DI**GAKIA**A**VQF**TY.....**D**Q**RTEFSF**T......D**YNTK**E**NVL**A**VL**A**NIR**Y..-----**MSGGTAT**.**GDAI**A**FT**V**R**N....V**F**GPIR**DS**PN-----.--K**N**F**LVIVTDGQS**YD-.....-----**DV**.**R**G**PA**AA**AHD**A**G**...**I**T**IFSVGVA**W**A**.....**PLD**D**LRD**M**AS**K**P**KESHAF--.---FTREF**T**G**LE**P**IVS**D**V**I

ITA1_RAT/1-191 Q**LD**.**IVIVLD**..**GS**N**SI**-........-YP**WE**S**V**IA**FL**.N**DLLKRMD**IG.....P**K**Q**TQVGIVQYG**E.....**NVTHEFNL**N......K**YSS**TE**EVL**V**A**A**NKI**GR..----Q**GG**LQ**T**M**T**.**A**L**GID**T**ARKE**....A**FT**EAR**GAR**RGV---.--K**KV**M**VIVTDGES**H--.....--DNYR**L**.**KQV**I**QDCED**E**N**...**I**QR**FSIAI**L**G**HY(8)KFVE**EIKSIAS**E**PT**EKHFFN-.VSDELA**L**V**T**I**VK**A**LGERI**F

YNX3_CAEEL/1-18 K**TD**.**LVFLID**..**GS**G**SIG**.......SY**V**F**KNEV**L**RFV**.R**E**F**VE**L**F**EIG.....R**S**K**TRVGLIQYSD**.....**Q**I**RHEFDL**D......Q**Y**G**DRDSLLKGISE**T**Q**Y..-----**LTG**L**T**R**T**.**G**A**AIQH**MV**QE**....G**FS**E**R**R**GAR**PQQ--S.DIA**RV**A**IILTDGRS**QD-.....-----**NV**.TG**PADSARK**L**S**...**I**NT**FAIGVTD**H....VLA**SELESIAG**S**PN**RWFY---.VDKFKD**L**D**T**R**LRSMIQKA**A

Q23409/1-188 M**LD**.**VII**AF**D**..**SS**G**SIS**........DEM**Y**YA**T**VG**AV**.N**TI**G**N**A**IS**IG(4)RILL**GT**YD**AI**S**HFSG**.....**D**L**NTL**D**TF**-.......----EAY**QN**K**L**A**DL**--..----FSL**G**Y**TGI**N**GN**N**IQ**S**VMDY**....-**I**VL**Q**N**NS**APFRPAP.-VR**K**F**L**M**LL**SSQGW**D**KG(4)GKENGFS**D**P.AP**AARNLHK**L**G**...**L**ET**FAIGLGTS**....A**NMTQLN**A**IA**KC**ST**Q------.---VNDQN**E**L**TSTI**S**QII**S

O51194/1-178 K**L**R.**I**S**FI**F**D**..I**S**R**SM**LSVD.EGKII**NRLE**S**AKNMI**.SL**ILSNF**E.........**NAE**Y**SL**TI**F**K**G**.....**KSKL**V**LPF**-.......S**KDKNSL**N**KMLN**Y**IE**P..--DLIS**SPGS**F**L**.**GDAV**FS**VIS**N....----VP**DDS**Y-----.--Y**N**F**LVILTDGDD**W--.....--GEN**NY**.**YR**F**SK**F**VNN**LK...**L**ES**FVVGIGGS**....N**P**VL**F**N**QNLS**IK**D**KNGN----.LVKTGINE**E**N**L**LL**LAS**S**L**K

O73756/1-201 K**LD**.**IYI**A**VD**..**VS**D**SI**-........-**NDLKKAKQII**.K**TLLEKIS**YY.....EV**SPS**YE**IL**M**FAT**.....**DV**YQ**I**VK**M**RDFKTEEV**A**G**SLSKVFE**D**LDK**F**D**F..-DKKLDQK**GSNI**.**AK**LY**Q**TI**LD**S....MS**N**E**Q**IR**NK**EDFLQT.--K**HVVIV**F**TDGQAN**MG(4)PKVHLIR**NL**.VLK**ND**AN**RE**NK...**L**D**LYV**F**GVG**K**D**....VRTED**LN**G**L**V**S**EKENERHFF-.---KLQDL**D**E**VQK**T**FDIM**L

Q26020/1-182 E**VD**.**LYLL**M**D**..**CS**G**SI**R.......RH**N**WV**NHA**VP**L**A.M**KLIQQLN**LN.....D**NA**I**HL**YASV**FSN**.....**NAR**E**II**R**L**HS....DAS**KNK**E**K**A**L**I**IIKSL**LS..--TNLPY**G**K**TNL**.**TDAL**L**QVRKH**....LN**D**RINRE**N**A-----.--N**QLVVILTDG**I**PD**--.....--SIQ**D**S.**LK**E**SRKLSD**R**G**...**V**K**I**A**V**F**GIG**Q**G**....I**N**V**AF**N**R**F**L**V**G**CH**P**SDG----.--KCNL**Y**A**D**S**A**W**E**N**VK**N**V**I

O31849/1-192 D**A**V.F**TLLVD**..**CS**A**SM**-........F**DKMDETKRGI**.VLFH**E**A**L**KS.......V**AV**PHQ**IV**G**F**WE(11)NYF**NT**V**IPF**Q.......S**S**L**R**Q**D**SGP**AI**M**QLE**P..------EE**D**NRD.**G**Y**AIRQ**MT**KK**....-----MLH**R**SEA---.--Q**K**F**LIV**FS**DGEPA**AFG..YEQNGIV**DT**.S**EAV**I**EARK**R**G**...**I**E**VINV**F**LSNS**E...IE**ESQ**M**KTI**Q**D**MY**G**KFSIF--.VPDVDQ**L**P**D**V**L**YP**LLKKL**L

O26551/1-166 R**A**L.Y**IIVLD**..**TS**S**SM**R.......LER**KIKFAKTV**S.WL**LLRD**SYE.......**K**R**NRIALI**A**F**R**G**....Y**EANL**VVEP-.......**TSNL**E**TVEEALE**G**LR**-..-----S**GG**R**TPL**.**T**P**ALRLA**A**E**V....-A**S**S**S**S**D**EAC-----.----TA**VVI**S**DGRCN**VF.....INSNLEE.DM**N**ML**ETE**LR**N**...**L**N**LL**F**V**NAE**P**-.....E**K**RS**L**GI**L**E**D**M**AS**R------.------**F**G**S**E**I**FY**L**D**DIL**I

P90573/1-181 Q**ID**.**LHILLD**..**GS**G**SIG**.......HS**N**W**ISHV**IP**ML**.T**TLVDNLN**IS.....R**D**EI**NIS**MTL**FST**.....Y**AR**E**L**VR**L**KR....YGS**TSKASLR**F**II**A**QLQ**N..--NYSPH**GTTNL**.**TSAL**LN**V**D**NL**....I**QK**KMNR**PN**A-----.--I**QLVIILTDG**I**PN**--.....--NLKKS.T**TVVNQLKK**K**D**...**V**N**V**AI**IGVGAG**....V**N**NM**F**N**R**I**L**V**G**C**G**KLGPCPY-.------**Y**SYGSW**DQAQ**T**M**I

YEHP_ECOLI/1-16 Q**W**Q.**LVLLVD**..Q**S**G**SM**-........-**VD**SV**I**H**S**A**V**M.AAC**L**W**QLP**.........**G**I**R**TH**LV**A**F**D**T**.....**SV**VD**L**T**A**--.......--**D**V**AD**PV**ELL**M**KVQ**-..-----**LGGGTNI**.**ASAVEYGRQL**....-----IEQPA-----.--K**SVIILV**S**D**FYE**G**G-.....--SSSL**L**.T**H**Q**VKKC**V**Q**S**G**...**I**K**VLGLA**AL**DS**T...A**T**P**CY**D**RD**T**A**QAL**V**NVGAQI-.------**A**AMTPG**ELASWL**A

Y077_METJA/1-16 C**GD**.F**V**VC**LD**..L**S**G**SM**R........G**NKE**I**WAK**A**I**A.L**CL**M**D**ISLK.......**RN**K**R**YIS**I**L**F**D**D**.....G**VR**D**IK**I**Y**-.......-**E**K**KVS**FD**EILE**FA**S**V..----F**YGGGTNF**.E**K**P**LREALKF**....-------.-------.--NGD**IV**F**ITDGEC**E--.....--VSLE**F**.**LE**KI**KE**E**KQ**RR..K**I**K**IYSI**C**INT**K.....**P**T**V**S**LRQISD**V**SV**TI-----.---YEL**T**S**K**T**AEK**V**FDML**I

E1372284/1-186 PK**D**.**VVILVD**..**VS**G**SM**K........GL**RLTIAKQTV**.S**SILDTLG**........**DDD**FFN**IITY**NE.....**E**L**HY**V**EP**CLN(4)QADR**TNK**E**H**F**REHLDKL**--..----F**A**K**G**IGM**L**.**D**I**ALNEAFNI**....LS**D**F**N**H**TGQ**GSI---.-CS**Q**A**I**M**LITDG**A**VD**T-.....--YDTI**F**.A**K**Y**N**WPD**RK**VR...**I**FT**Y**L**IG**RE**AA**.....FA**D**N**LK**WM**AC**A**N**KGFFTQ--.ISTLAD**V**Q**E**N**V**M**EYLH**V**L**S

O08746_1/1-183 R**AD**.**LVFIID**..**SS**R**S**V**N**........TY**DY**A**KVKEFI**.L**DILQ**F**LD**IG.....P**DVTRVGLLQYGS**.....**TVK**N**EFSL**K......T**FK**R**KSEVERAVKR**M**R**-..----H**LSTGT**M**T**.**G**L**AIQYALNI**....A**FS**EAE**GAR**PLRE--.NVP**RII**M**IVTDGRP**QD-.....-----**SV**.A**EVA**A**KARN**T**G**...**I**L**IFAIGVG**Q**V**.....**DLN**T**LK**A**IGS**E**P**HKDHVFL-.VANFSQ**I**E**S**L**TS**V**F**Q**NKL**C

CA1C_CHICK/1-18 K**AD**.**VVFLVD**..**GS**Y**SIG**........I**ANF**V**KVR**A**FL**.EV**LVKSF**EIS.....P**R**K**VQISLVQYS**R.....**DPHMEFSL**N......R**YN**RVK**DIIQAINT**FPY..-----R**GGSTNT**.**GKA**M**TYVREK**....V**F**VT**S**K**GSR**P-----.NVP**RV**M**ILITDGKSS**D-.....-----**AF**.**KEPA**I**KLRD**A**D**...**V**E**IFAVGV**K**DA**.....**VRTELE**A**IAS**P**PA**ETHVYT-.VEDFDA**F**Q**R**ISF**ELTQ**S**V**C

O17530/1-177 W**LD**.**VYLLID**..**NS**AK**MG**SVG.LLEVA**SN**V**N**S**V**FG**F**T.QIR**V**G**S**NYPD.....K**RGARVSVLTYSD**.....**SPTVH**A**NL**S......D**FKS**T**DEL**T**SMI**YA**LK**P..----S**TS**Y**DSNL**.Q**S**DY**DEP**TIA....---QFG**D**QLKADG--.---VK**II**T**V**A**D**I**SNT**D-.....-HQHV**S**K.**LK**WL**KEL**A**S**E-...--GN**G**FN**INDD**Y...V**SE**EVQ**K**A**L**C**S**A**NC**F------.------**C**P**R**K**YHHFIT**SNN

BCHD_RHOSH/1-17 DRV.**LIF**A**VD**..**AS**G**S**S**A**........M**ARLSEAK**G**AV**.EL**LL**G**Q**AYA.......**R**R**DHVSLL**A**F**R**G**....R**DAELILP**P-.......**TRSLVQ**T**KR**R**L**AG**L**P-..-----**GGGGTPL**.**AHGLRLAL**AV....---GLQ**A**RARGM---.--TPT**V**A**LLTDGRGN**IA(5)NRAQAEE**DA**.**LK**L**A**A**SLR**GS**G**...**L**PAV**VI**DT**AN**R.....**PQP**S**L**AA**LA**RAL**D**AP-----.---YIA**L**P**R**ADA**HKLS**N**V**L

CIC2_RABIT/1-17 PK**D**.M**LILVD**..**VS**G**S**V**S**........GL**TLKL**I**RT**S**V**.S**E**M**LETLS**........**DDD**F**V**N**V**A**SF**N**S**.....**NAQ**DVS**CF**QHLV.QAN**VRNK**KV**LKDAVNNIT**-..-----**A**K**G**I**TDY**.K**KG**F**SFAFEQ**....L**LN**Y**N**V**S**RAN-----.-CN**KII**M**L**F**TDG**GEE--.....-----R**A**.**QE**IFA**KYNK**DK..K**V**R**VFT**F**SVG**QHN...Y**DRG**P**IQ**WM**AC**E**N**KGY-----.---YYE**I**P**S**I**G**AI**RIN**TQE

E1347646/1-165 CK**D**.**ILFLLD**..**SS**GNV-........VQ**QYEK**Q**KKYI**.E**EIVRKL**E........**HP**R**R**M**ALITFAG**R...T**R**Q**KI**V**IPL**P......EEP**N**GK**K**F**IE**K**LRK**A**R**F..-----**L**R**GVTAA**.**G**A**AIS**V**T**T**QY**....----VLQK**S**R-----.--HVQ**VVVVTDG**F**S**FD-.....-----**DV**.**EK**Q**SE**A**LR**AV-...**V**GM**ET**FVT**G**RYF..PV**VKN**V**LHSIGG**A**DD**HVF----.------**F**D**K**KE**QKLID**A**L**Q

Q63870_1/1-183 P**VD**.**VVFLL**H..**A**TRDN-........AH**N**A**E**A**VRRVL**.E**RLVS**A**LG**PL....GP**QAAQVGLLTYS**H.....**RPS**P**LFPL**N......SS**HDLG**I**ILR**K**IRDI**PY..----VD**PSG**N**NL**.**GTAVT**T**AHRY**....L**L**AS**N**A**PGR**RQQ---.-VPG**V**M**VLL**V**D**EPLRG-.....-----**DI**.**LSP**I**REAQT**S**G**...**L**K**VMALSLVGA**.....**D**PE**QLRRLAP**G**TD**PIQNFFA.VDNGPG**L**D**R**A**VSDLA**VA**L**C

G4104232/1-176 KK**D**.**VVFLID**..**GS**RNA-........G**PEFQY**I**RTLI**.E**RIVE**Y**LD**IG.....F**DTTRVAVIQFS**E.....**DSKMEFPL**N......A**H**F**SKDEVQNAVRRLR**P..----K**GGS**QVY**I**.**GNALEYVLK**N....I**FQ**RPL**GSR**IEEG--.-VP**Q**F**LVLI**SS**GKSD**D-.....-----E**V**.D**DSA**V**ELKQ**F**G**...**V**AP**LTIA**RH**T**-.....**DQ**E**EL**V**KIS**LS**P**EY------.VYSVST**F**R**E**LP**R**L**E**Q**KLL**K

O84941/1-227 G**AD**.**VM**A**LLD**..**VS**KK**MS**........E**DDFN**N**AKN**K**I**.K**KLVKTLT**SK(11)YN**S**R**NSV**R**L**M**TF**YR.....**E**I**S**NP**IDI**S.......**GKTE**E**QL**D**KLLDDLR**K..KAKAN**YD**W**G**V**DL**.QG**AIHKAREI**....FN**K**E**K**EKKFG-----.-KR**R**H**IVL**FSQ**GEST**FS(35)HTHKNI**DM**.**LDDVKNL**V**K**L**G**...QT**L**GIA**GLDN**-.....L**QS**T**LS**L**IST**G**SS**LAGAF--.--------LG**G**G**SLTEYL**T

CGHU2A_1/1-184 P**VD**.**IVFLLD**..**GS**ER**LG**........EQ**NFHKARRFV**.E**QV**A**RRLT**LARRDDDPL**NARVALLQFGG**P...G**E**Q**QV**A**FPL**-.......S**HNLT**A**IHEALET**T**Q**Y..-----**LNS**F**S**H**V**.**G**A**GV**V**HAIN**A...IV**RS**P**R**G**GAR**RHA---.--ELSF**V**F**LTDG**V**TG**--.....---ND**SL**.**HESAHSMRN**E**N**...**V**VPT**VLALGSD**....V**DMD**V**LTTLS**LG**D**RAAVFH--.---EKD**Y**D**S**L**AQ**PG**F**FDRF

O31191/1-264 KPN.**IMLLVD**..**TS**G**SMT**YP(27)VCP**TRWT**TL**QSVV**.P**Q**F**LEN**S**G**........**R**F**VR**F**AL**T**TY**PE.....**T**RG**GE**SIPDL....CR**AST**A**G**A**LLKTL**P**E**Q**E**D(22)EGRPL**GGTPT**.**S**G**SLNFV**GG**L**....----**E**GLQ**D**LDR---.--E**N**F**VILLTDG**L**PN**CN(28)RGCLDT**DA**SV**TAVRELHE**K**G**...**I**QT**IVIG**F**GA**ETAVGD**G**P**A**V**LE**AM**A**RA**GG**FKRT---.------**C**SAERP**C**GEGD**T**C

O45052/1-174 E**LD**.**LVLVLD**..F**S**TTT-........D**P**V**YN**SY**KDL**S.K**RLVSQL**KIG.....P**H**Y**TQVA**A**VTFAT**V...G**RTRVRFNL**K......K**YQTQ**E**EVLRGIDNLK**S..-----R**GGTTAI**.**G**A**GIEKALTQ**....-**LD**E**S**E**GAR**PGI---.-AT**KV**M**VV**F**TDG**W**SN**KG.....----P**D**P.**EK**R**ARDA**V**S**S**G**...FEM**YTVA**Y**T**R**V**E...TR**QTY**C**TN**F**S**LCF**P**S------.------**L**P**R**A**TTSL**S**SLT**I

P79816/1-207 T**L**N.**IYI**A**LD**..I**S**E**S**VE........EE**HFKRAK**L**AI**.I**TLIKKIA**AF.....TV**SPN**YE**IL**F**FSA**.....**DV**YEVV**SI**VE.....F**YEGK**I**TLESAIKNLE**D..----FQI**GD**K**ST**.**GT**D**VN**A**ALKK**....----FEE**G**MAWIE(8)EHR**HV**F**LL**F**TDG**AY**N**MG(5)TLARIKNR**V**.**Y**M**SPT**GDPGSRLDY**L**ES**YV**F**GIGAN**I...F**D**D**DLL**PLT**AG**TE**G**ELHYFR-.LKKETN**L**AAT**FDDIIDE**NE

3844596/1-191 T**T**N.**LIF**G**ID**..YTA**S**NKYQ(15)HVT**N**P**YQQV**I**SIL**.G**R**T**L**AP**FA**........GQ**GRLGV**YG**FGD**....A**KT**GD**W**S**VF**NLKGEGGD**CRSLDEVLNV**Y**NTVT**P..--TVA**LSGPTNF**.**A**PL**I**Y**QAMEI**....----**C**QK**SR**D-----.--Y**HILVII**A**DGQVT**--.....--NER**AT**.**RRA**IV**QACQ**H**P**...**L**S**IIVVGVGDG**.....**PWDM**M**R**IFDESL**P**K------.------RPWDNF**HFVEFH**E

O01703/1-173 KQ**D**.T**LFLID**..**SS**LKD**S**........N**VTFRI**L**QQF**A.V**Q**A**VQ**P**FN**YV.....SGL**GQVA**A**LR**V**AD**.....**KA**Y**G**G**FSY**NA.....GE**NSFDRV**S**ELI**Y**N**M**Q**Y..----I**G**I**DG**Q**NV**.**T**A**GLQYALDY**....YDLP**S**Q**G**Y**R**TGS---.DVR**HLLV**Y**VT**QT**NPT**DA.....-----**D**P.S**E**LL**RTIKR**S**G**..LYE**V**V**VVAL**---.....**DM**QPS**DKL**T**N**M**VN**PR-----.------**C**FYL**AQDFHDLM**N

G3925399/1-184 D**L**A.**VTLLVD**..**VS**L**S**T**D**SW...FNDRRV**LD**VE**KEAL**.MV**L**A**E**G**LS**A.......**CGDN**H**SILTF**T**S**.....**R**R**R**D**W**VRVETIK.AFDEPMSHA**VRR**R**I**A**SLK**P..------**G**YY**T**R**I**.**G**A**AIRHA**AA**K**....-----L**S**E**Q**PNR---.--K**HL**M**LVLTDGKPN**DVDHYEGRFALE**DT**.**RRSV**I**EARR**K**G**...**V**Q**VFGV**T**VD**Q**D**.....**AKSYV**PAMF**G**QH**G**FAV----.------**V**P**D**IR**K**LPSA**L**PA

O05809/1-178 GNL.**VIFVVD**..**AS**G**SMA**.......AR**DRM**AA**VS**G**A**T.L**SLLRD**AYQ.......**R**R**DKVAVITF**RQ....H**EATLLLS**P-.......**TSS**AHIAG**R**R**L**A**R**F**S**-..-----**TGG**K**TPL**.**AEGL**LA**AR**A**L**....I**IR**E**K**VR**DR**ARR---.---P**LVVVLTDGRAT**AG.....PDPLGRS.**RTAA**AG**L**VAE**G**...AAAV**VV**DCE**T**-.....**SYVRL**GLA**A**QL**A**RQLGAPV-.---VRLEQLH**AD**Y**LVH**A**V**R

CA36_CHICK_6/1- KK**D**.**ILFLID**..**GS**AN**L**-........L**GSF**PA**VRDFI**.H**KVISDLN**VG.....P**DATRVAV**A**QFSD**.....**N**I**QIEFDF**A......E**L**P**SK**Q**D**M**L**LK**VKR**M**R**L..----K**TG**KQL**NI**.**G**V**ALDEVMRR**....L**F**VK**E**A**GSR**IEEG--.-IP**Q**F**LVLL**AA**GRST**D-.....-----E**V**.**ERPS**GA**LKE**A**G**...**V**VT**FAI**KAK**NA**.....**DLSELERIA**YA**P**QF------.ILNVES**L**P**R**IS**E**L**QANIV**N

CA36_CHICK_5/1- EK**D**.**VVFLID**..**SS**D**S**VR........S**D**G**L**A**H**I**RDFI**.S**RIVQQLD**VG.....P**N**K**VRIGVVQFSN**.....**NV**FP**EF**Y**L**R......T**HKSKN**A**VLQAIRRLR**L..----R**GG**Y**P**V**NA**.**GKALDYV**V**K**N....Y**F**IK**S**A**GSR**IEDG--.-VP**Q**H**LVVI**LGD**QS**QD-.....-----**DV**.N**RPAN**V**ISS**T**S**...**I**QP**LGVG**AR**NV**.....**DRNQLQ**V**I**T**N**D**PG**RVLV---.VQDFTG**L**P**T**LE**RK**VQ**NIL**E

G3928084/1-180 P**VD**.**LV**T**VLD**..I**S**G**SMA**........G**TKL**A**L**L**KRA**M.GF**VIQNLG**........**SNDRLSVI**A**FSS**.....**TAR**R**LFPL**TK....MSDA**GR**Q**R**A**LQAVNSV**V-..-----**ANGGTNI**.**AEGLRKG**V**K**V....-**ME**D**R**R**D**K**N**P-----.--VAS**IILL**S**DGRDT**YT(5)PNYKLLL**PL**.SMH**GCE**S**KR**FQ...**I**P**VHS**F**G**F**GSD**....H**D**A**SL**M**HSVS**ET**SG**GTF----.------SFIES**ES**V**IQ**D**A**L

D1035690/1-206 R**L**N.**VFILLD**..**TS**A**SIS**........PE**SFHLAKNA**T.I**QLVQKLD**SY.....EV**T**M**R**F**GIISYAS**.....**EAK**E**I**V**SI**TN....DLS**QD**VHY**VMR**K**LHE**F**S**D..-KSHGNKR**GTNL**.H**DALNKVYEE**....LALL**R**E**N**K**R**SHFNET.--Q**NVIII**A**TDG**Y**SN**MG(7)PKIRNLF**GY**.**KSSVDHTKE**EL...**L**D**VYV**F**AVG**QQ....V**NK**Q**ELQSIAS**IKKDERHVFV.LKDYRQ**L**GLV**FNQMIS**DSA

CA36_CHICK_9/1- P**T**E.**LAF**A**ID**..**TS**SGV**G**........R**D**V**FNR**M**KQTV**.L**RVVSNLT**IAES.NCP**RGARVALVTY**N**N**.....**EVTTEI**R**F**A......D**AR**K**KSSLLQ**Q**IQN**F**Q**AT.----L**TT**K**P**R**SL**.E**TA**M**SFV**A**R**N....T**FK**RAR**SG**FLMR---.---**KV**A**V**FFSN**GET**R--.....--ASPQ**L**.N**DAV**L**KL**Y**D**A**G**...**V**TPVF**L**TSRQ-.....**D**A**VL**E**R**A**L**EIN**NT**AVGHAIV.---LPTSG**S**Q**LND**T**IRRL**L

Q90583/1-188 P**I**S.**VYFVID**..**TS**E**SIA**LQT..VPIQ**S**LV**DQ**I**KQFI**.P**R**F**IEKL**ENE(4)QVSI**T**WMF**G**G**LHYSD**.....V**VEIY**S**PL**-.......**TRSKDT**Y**LT**K**LR**A**IR**Y..-----**LG**R**GT**F**T**.**DCAIS**NMT**QQ**....F**QS**Q**T**AR**D**VK-----.----FA**VVITDGHVT**GS.....--PCG**GM**.**K**MQ**AERARD**M**G**...**I**K**LFAVA**P**S**E**D**....VY**E**QG**LREIAS**P**P**HDLY----.RSNYTI**T**P**K**D**A**L**HI**D**E**N**T**I

Y0D7_MYCTU/1-18 G**V**A.A**TI**M**LD**..Q**S**MPN**D**E.....GGN**SRLS**N**V**VA**AL**.E**N**R**IK**A**MP**........P**SS**V**VGL**W**TF**D**G**R..EG**RTEV**PA**G**PLADP.VNGQPRP**A**A**L**TA**AL**G----..------KQY**SSG**.**G**G**AVSFT**TL**R**....L**I**YQ**E**ML**AN**YRVGQA.---**N**S**VLVIT**A**G**PH**T**DQT..LDGPGLQ**DF**.**IR**K**S**A**D**PA**K**P-...**I**A**V**NI**I**DF**GAD**....P**DRA**TW**E**A**VA**QL**SG**GSY----.------QNLE**TS**ASP**DLA**T

O76836/1-179 V**LD**.M**II**AF**D**..**TS**E**SLS**........SLIVP**Q**YV**DF**A.K**KLV**A**QY**KYG.....N**DNTRVGIITFSS**.....**DV**VEV**R**K**L**T......D**GNTLD**A**V**NA**AIDTVH**Y..-----**TGG**L**TNV**.**TKA**QLT**AKNL**....-**FD**T**E**S**NAN**R-----.--N**KVL**F**ILTDG**V**PT**V-.....-DTYT**D**E.VA**AGDKLKS**I**S**...**V**IS**F**F**VG**Y**SS**YS..DE**VKTEL**G**KVS**EPKYIFGN---.---MTF**L**P**E**I**T**A**QILT**T**Y**P

ITAM_MOUSE/1-18 ES**D**.**IVFLID**..**GS**G**SIN**........NI**DFQK**M**KEFV**.S**TV**M**EQF**KK.......**S**K**T**LF**SL**M**QYSD**.....**E**F**RIHFTF**N......D**FK**RN**PS**P**RSHVS**P**IK**Q..-----**LNG**R**T**K**T**.**ASGIRKV**V**RE**....L**FH**K**T**N**GAR**ENA---.--A**KILVVITDGE**KFG-.....--DPL**DY**.**KDV**IP**EADR**A**G**...**V**IR**YVIGVGNA**FNKPQ**SR**R**ELDTIAS**K**PA**GEHVFQ-.VDNFEA**L**N**T**IQ**NQL**Q**EKI**F

Q25757_3/1-187 T**YD**.**LTLIID**..E**S**A**SIG**.......YS**N**W**EKEV**VP**F**T.IG**L**A**SNL**EIS.....E**K**K**VN**M**GIL**L**FSD**.....**K**I**R**E**FI**K**Y**GQ....KESY**DKNNL**V**R**R**IHDLK**KY.---YKS**GG**F**S**Y**I**.**VEALKYGL**YS....YA**K**S**T**S**S**RLN-----.-VP**KV**N**ILLTDGNNT**D-.....-TSDFI**L**.T**EVSS**L**YKK**E**N**...**V**K**LL**L**IGIGGP**.....**TI**H**KLR**L**LGG**C**D**KSDGD---.CPYVVK**A**EWNNL**KYTS**N**L**I

CHLD_PEA/1-196 G**A**L.**VIFVVD**..**AS**G**SMA**........L**NRMQ**N**AK**G**A**A.L**KLL**A**E**SYT.......**S**R**DQVSII**P**F**R**G**....D**SAEVLLP**P-.......S**RSIA**MA**RK**R**LERL**P-..-----**CGGGSPL**.**AHGLT**T**A**V**R**V...GLNAE**K**S**GD**VG-----.--RIM**IV**A**ITDGRAN**IS(19)SQELKDE**I**.**IEVA**A**KI**Y**K**T**G**...MS**LLVI**DTE**N**KF...V**S**T**GF**A**KEIA**RV**A**QGKYYY--.------**L**P**N**AS**D**AV**VSLA**T

O00339/1-179 P**ID**.**LVFVID**..**GS**K**SLG**........EE**NFE**V**VKQFV**.TG**IIDSLT**IS.....P**KAARVGLLQYST**.....**QVHTEFTL**R......N**FNS**AK**D**M**KKAV**A**H**M**K**Y..-----**MG**K**GS**M**T**.**G**L**ALKH**M**FER**....S**FT**QGE**GAR**P---FSTRVP**R**AA**IV**F**TDGRA**QD-.....-----**DV**.S**E**W**ASKAK**AN**G**...**I**TM**YAVGVG**K**A**.....I**E**E**ELQEIAS**E**PT**NKHLF--.---YAEDF**S**T**MDEI**S**EKL**K

YP72_CAEEL/1-19 YSN.**LIF**G**ID**..YTK**S**NFYQ(15)AEM**N**P**YQQV**I**QIV**.G**K**T**LSSFD**........A**DGQI**PAYG**FGD**(7)IF**N**IAE**RYDL**EK.....D**CNGF**E**EVLRV**Y**NEVT**P..--TIE**MSGPTNF**.**V**PL**IDRAIEI**....---C**K**EKH**S**Y-----.---**HILVIV**A**DGQVT**--.....--NEKIN.**QKA**IAA**ASH**Y**P**...**L**S**II**M**VGVGDG**.....**PWNM**MG**R**FD**D**NI**P**K------.------RLFDNF**HFVDFH**K

YNOB_PSEAE/1-18 D**L**A.C**LLL**A**D**..L**S**M**S**T-........E**A**Y**LD**DQ**RRVI**.D**SIVDSL**LLFGEALQAL**GD**PF**AL**YG**FSS**V...R**R**Q**QVRW**QVLKD...FDEGYG**GEVR**GR**V**LA**LS**P..------**G**YY**T**R**M**.**G**A**AIRRA**S**Q**V....-----L**GGQ**PQK---.--R**RLLLLL**S**DGKPN**DLDRYEGRYGIE**DT**.**RQAV**I**EARS**Q**G**...**L**VP**FCI**T**ID**KE.....**A**A**DYL**PY**L**F**G**A**DG**FA-----.------**L**V**E**R**A**G**QL**P**ERL**L

WORM|CE19980|K0 P**TD**.**LVFLID**..**GS**G**SIG**.......SY**V**F**QTEV**L**RFL**.A**E**FT**E**L**FD**IA.....P**Q**K**TRVSVVQYSD**.....**Q**I**RHEFGL**D......N**YSDR**K**SLQNAIRNIE**Y..-----**LTG**L**T**R**T**.**G**A**AIEHV**A**NE**....A**FS**E**R**R**GAR**PVG---.QVS**RV**A**IVITDGRS**QD-.....-----**NV**.T**RPSDNARR**Q**D**...**I**Q**LFAVGVTN**H....VLD**AELEEISG**SK**D**RTFH---.VSGFED**L**N**T**R**LRS**A**IQRV**A

CA1C_MOUSE/1-18 W**TD**.**LVFLVD**..**GS**W**S**V**G**........R**NNFKY**IL**DFI**.VA**LVS**A**FD**IG.....E**E**K**TRVGVVQYSS**.....**DTRTEFNL**N......Q**Y**YR**R**E**DLL**A**AVKKI**PY..-----K**GGNT**M**T**.**GDAIDY**LV**K**N....T**FT**E**S**A**GSR**AGF---.--P**KV**G**IIITDGKS**QD-.....-----E**V**.**E**I**PARELRN**I**G**...**V**E**VFSLGI**K**AA**.....**D**AK**ELKQIAS**T**PS**LNHVFN-.VANFDA**I**V**D**IQ**NEIISQV**C

2828245/1-180 G**T**N.**LVLLID**..R**S**SQ**H**WGD(8)GGDE**SK**AAA**ARRIL**.L**D**F**I**A**RRP**.........**GGRIGV**AA**FST**.....A**P**M**L**V**LPM**-.......**TESRT**A**I**AA**AV**AA**L**AE..----P**G**L**S**Q**TDV**.**GRGLTLAM**G**M**....-A**H**EAS**ASD**S-----.---**R**A**VVLV**S**DG**A**AV**I-.....-RARG**P**D.**R**LR**N**LA**ARR**Q-...**V**N**IY**W**L**Y**L**R**T**-.....-**KG**AKGIFE**V**PE**P**GQ-----.-ADTPH**L**RPER**H**L**HI**F**L**QS

G3790754/1-170 R**TD**.**ILFLLD**..**SS**DN**FT**........EQ**KFQRA**I**KLI**.G**E**T**V**G**QFS**NF....GA**DGVQVSLVQY**N**D**.....**EP**Y**LEFSL**R......K**HNCK**K**HLL**AD**I**A**D**T**E**F..-----**MTGGS**Q**L**.**NKALEKV**S**QF**....A**FT**K**K**R**GDR**PDA---.--E**NILIIVTDGQSD**A-.....-----R**I**.**QEPTR**L**VKD**H**N**...**V**T**VLVIS**TLE**A**.....**DKNFI**I**NLSA**E**N**LY------.------DS**D**N**F**G**K**D**L**A**W**KL

Q19198/1-226 ---.F**FFILD**..**TS**G**SMS**TRA..HPQF**S**F**FDLAKNFI**.E**N**F**IKQRT**KG(5)VGR**ETDK**YF**L**M**T**TQ**A**.....**R**YPKNVKVA......CE**K**LG**A**V**VIE**E**IKKLH**L..-----PY**GS**CQ**L**.H**HAI**L**EAFK**V(4)RV**QT**GID**GV**GIGRLISNTEPVTM**ILLTDGSGV**AG(28)FYTVVFR**I**.P**STP**Y**R**P**T**IS-...-Q**L**T**AI**D**I**---.....**DMP**V**IERL**C**A**R**TG**GRSFSIV.------SP**R**Q**IQT**T**IDYL**L

O04042/1-198 E**L**N.F**M**VA**VD**..FTA**S**N**G**DP(8)IDPS**GRLN**SY**QQAI**.M**EV**G**E**V**I**QFY.....D**SD**K**R**FPAWG**FGG**RTSDG**SVSH**A**FNL**NG(7)VGVEGI**MV**AYA**SALRNVS**-..-----**LAGPT**L**F**.**SN**V**VDKA**A**H**T...AS**QS**L**S**Q**NS**PKYF---.----**VLLIITDG**VL**T**--.....--DMA**GT**.V**DA**LV**RASD**L**P**...**L**S**VL**I**VGVGNT**.....**DF**K**Q**M**E**M**L**D**A**D**NG**RR-----.------**L**E**S**S**T**G**RIATR**DI

34628/1-206 H**L**N.**LYLLLD**..**CS**Q**S**V**S**........E**NDF**L**I**F**KE**SA.SLM**VDRI**FSF.....EI**NVSVAIITFAS**.....**EPKVLMS**VLN....DNS**RDMTEVIS**S**LEN**A**N**Y..-KDHEN**GTGTNT**.YA**ALN**S**VY**L**M**....-**MN**N**Q**MRLLGMET(4)EIR**H**A**IILLTDGKSN**MG(4)TAVDHIRE**I**.**LN**I**NQK**R**ND**Y-...**L**D**IYAIGVG**KLD...V**DW**R**ELNELGS**KK**D**GERHAFI.LQDTKA**L**H**Q**V**FEHMLD**VSK

CA36_CHICK_7/1- KR**D**.**VVFLVD**..**GS**RYA-........AQ**EF**Y**L**I**RDLI**.E**RIVNNLD**VG.....F**DTTRISVVQFS**E.....**HPHVEF**L**L**N......A**HSTKDEVQ**G**AVRRLR**P..----R**GG**QQV**NV**.**GEALEFV**A**K**T....I**FT**RPS**GSR**IEEG--.-VP**Q**F**LVIL**SSR**KSD**D-.....-----**DL**.**E**F**PS**V**QVKQ**V**G**...**V**AP**MVIA**K**N**M-.....**D**PE**E**MV**QIS**LS**PD**YVFQ---.VSSFQE**L**P**S**LE**QKLL**AP**I**E

Q20732/1-180 T**AD**.**VFFLVD**..**VS**QGT-........G**DK**S**QQ**YL**DI**A.A**S**A**ISSLP**IS.....Q**DTVRVGLISYSG**P...G**RTHVR**VF**L**D......K**HN**E**K**E**KLIE**EMFLM**E**R..-----**HGGTT**R**T**.**ADAIRYA**T**KI**....-**FE**G**K**AH**P**ARKN---.-VK**KVLVV**F**TDG**Y**S**QD-.....-----**N**P.**KEASR**M**AR**AK**G**...**I**Q**LIAVAV**K**D**RLA.PP**D**TE**QLTEIGG**N**G**RSIFI---.---SPS**G**R**D**LR**EKII**GTQC

O42401/1-180 P**LD**.**LVFIID**..**SS**R**S**VR........PE**EFEKVK**I**FL**.S**K**M**IDTLD**VG.....E**RTTRVAV**M**NYAS**.....**TVKVEFPL**R......T**Y**F**DKAS**M**KEAVSRIQ**P..-----**LSAGT**M**T**.**G**L**AIQ**A**AMDE**....V**FT**E**E**M**GTR**PANF--.NIP**KVVIIVTDGRP**QD-.....-----Q**V**.**ENVA**A**NART**A**G**...**I**E**IYAVGVG**R**A**.....**DM**QS**LR**IM**AS**E**P**LDEHVFY-.---VET**Y**GVIE**K**LTS**KF**RE

YS02_MYCTU/1-17 R**A**V.**VMLVID**..**VS**Q**SM**RAT...DVEP**SRM**VA**AQEA**A.K**Q**FA**DELT**........P**G**I**NLGLI**A**YAG**.....**TATVL**V**S**P-.......**TTNR**EAT**KNALDKLQ**F..------**AD**R**TAT**.**GEAI**FT**ALQ**A....-**I**ATVG**AV**IGGGDTPP--PAR**IVL**FS**DGK**E**T**MPT...NPDNPK**GA**.**YTAARTAKD**Q**G**...**V**P**I**S**TIS**F**GT**-.....-PYGFV**EI**N**D**QRQPVP----.------**V**D**D**E**T**M**KKV**A**QL**S

Q18048/1-173 I**YD**.**IGII**F**D**..**SS**G**SL**-........EK**NFQK**QLA**F**A.K**QLVEQMP**IS.....D**NATRVGIVQFAG**K...T**KVRVL**A**NF**-.......S**QNKSQLKTIIDR**SPF..-----**YSGTT**F**T**.**NQALKK**MAA**L**....-**YE**E**S**KR**PN**A-----.--KLK**L**M**L**F**TDG**Y**SA**E-.....-----**DT**.S**EG**E**E**A**LKS**Q**G**...**V**V**VYTVGISTD**KSAGL**NM**K**ELR**GM**AT**S**S**EH------.---YYDSS**D**F**AD**L**L**K**HF**PS

P73233/1-170 EQT.**VIILLD**..RGRL**MT**AW...VQGLQ**RFDWG**L**NT**S.LA**L**AVAALH.......**RGDRLGI**GV**F**DR.....**EV**VS**WLP**PER.....G**LQ**Y**LPKLLKTLS**P**LQ**P..-----**V**LREP**DY**.**S**A**AI**G**K**LV**QQ**....-----Q**T**R**R**A-----.----**LVV**C**ITD**LI**DP**--.....--TASQE.**L**L**TA**L**T**QLAPR...YLP**FCV**T**L**---.....RD**PQVDQLA**HA**PA**TT-----.VSGAYQQAVA**IN**L**L**Q**QR**QL

Q94674/1-183 K**VD**.**LYLL**M**D**..**GS**G**SIG**.......YY**N**WV**TYA**VP**LV**.E**EIVQNLN**IS.....K**QG**I**HL**Y**L**SV**F**TH.....IL**K**E**YIPL**NS....IFS**TNRD**FA**LNVIRSLR**T..--KYSQ**NGSTNL**.**T**L**ALSRVLK**N....-**Y**FL**T**K**GSR**EDA---.--V**QLVII**F**TDGSPD**--.....--NKE**SA**.**MK**E**VNKLKK**MK...AKFA**VIGVG**M**G**....I**NK**E**F**N**KSL**V**G**C**P**LKE-----.-KKCDL**Y**S**E**ASW**NEVQ**N**V**I

G4104398/1-192 R**T**G.**IVLVVD**..**TS**V**SM**-........Q**P**Y**IDRVREVI**.DG**L**Q**RQIG**ERG...DL**D**K**VS**F**GLV**G**F**R**N**(8)EYV**SKTL**V**PL**QPG...ND**AQ**R**FAEL**S**S**Q**VR**AT**D**V..---SS**HS**F**N**E**DA**.FA**GI**M**QA**V**DE**....-----M**D**W**S**PYAG--.---**RVVLLVTD**AG**A**LRKND.PLGRTQM**N**E.A**EV**R**Q**A**A**L**R**K**G**...**V**K**IYAL**H**L**R**TP**....A**GKN**NHGYAEQQYRSL-----.------**T**A**D**ANP**KIADLY**I

E1350321/1-186 W**LD**.**IV**A**VVD**..**NS**KE**MT**........D**D**GVLSIAGE**L**.S**SL**F**SH**AEQL(4)NQP**RTTRIGLVTY**NE.....**EATV**VA**DL**N......N**ITT**A**DEL**S**NTL**FAALTW.---TSS**V**EH**S**Y**L**.Q**TGLK**A**A**D**DL**....LA**K**Q**S**F**NTS**RGH---.-YQ**KLVIV**YASEYR**V**S-.....-----**GT**.**QDP**LPL**ATR**MKT.Y**L**T**I**A**TVA**YRQ**D**....I**V**V**GF**N**D**A**L**TKI**AS**PGYNF--.------**T**NFN**GNN**V**VSEL**K

P70961/1-194 D**T**N.**VA**V**L**F**D**..**GS**G**SMV**QK...TGGER**KIDIAKK**S**V**.K**S**FA**E**L**LP**........**KDTNL**M**L**RV**FG**H(13)S**TTETIYGL**H.......PYEG**S**LFD**N**S**LSELK**P..------**TG**W**TPI**.**AKAL**AD**TRKE**....-**FE**AFD**AD**GK-----.---**NVV**Y**LITDGE**E**T**CGG...DPAAEIEK.**LRASN**VD**T**IV**N**...**I**IG**FN**FD**V**K**G**-.....-NE**E**M**KQ**A**AV**A**GG**GEYIS--.-ANSADEF**E**Q**A**W**EK**EA**Q**KF

3873808/1-197 PK**D**.**IVFLLD**..Y**S**G**S**VK........G**PTMHL**I**K**I**T**M.MY**ILSTLS**........P**ND**YFFG**V**Y**F**N**N**.....**H**F**N**P**IIS**CAN(4)PAT**TSNK**KVF**FE**E**L**GM**LE**E..--KDQ**A**HF**ATPL**.KF**SLD**VL**R**GN....LD**S**N**Q**SLFADYRSEG.--H**KLLII**F**TDG**V**D**EWP....HQILDEE**F**.**QT**R**NSEL**I**R**I-...-FG**FS**M**G**Y**GTS**.....L**LPL**Q**Q**YM**AC**K**S**HGGYSEIDSIMDVKPQS**R**T**IQN**V**LSQV**R

Q23561/1-187 W**LD**.**VV**V**VVD**..R**S**MQ**MT**........D**AQL**W**QVRQTL**.T**QV**FG**S**DIRI(4)SDK**RSTCVG**F**VTY**N**S**.....**NATV**TAE**L**D......I**AKSFPDIYNIIQ**GSLV..--DVAS**TNASSL**.**GEGL**LA**AQR**V....LN**S**G**R**QR**TN**RYN---.-VK**QVIL**AFAADFQ**D**EF.....--SNL**NA**.**IE**L**SRDLQS**N**G**...**I**S**IITVA**C**T**K-.....**D**SYA**L**P**RI**NMC**AT**PGYAFV-.------DEMN**TSKLVKQL**T

O16003/1-176 ---.M**LFLLD**..**SS**G**S**V**G**........DE**NFDK**M**KEFV**.K**SIV**L**NFD**VD.....N**Q**L**TRIGIIRF**D**S**.....**DAEIII**Q**L**S......D**HKTL**K**DLLN**D**IDSIR**YN.-----E**G**IQ**T**R**I**.**DKALERAME**A....-**FS**E**K**N**GGR**ADA---.--T**K**A**LVLL**A**DGQNS**F-.....IEGSQ**DL**.N**E**EL**K**P**L**I**D**AK...**V**FR**YVIGIG**RE....L**DL**K**ELEDIAT**N**N**IAI-----.---YADSF**D**E**LK**F**KVDE**QI

E1349852/1-200 Q**LD**.F**A**VA**VD**..FTA**S**N**G**PP(11)DRP**NQYELA**L**R**S**V**.L**SI**C**QHYN**........**SS**K**T**FEAFG**FGA**(4)QS**SVSAIFPL**DL(7)VGI**T**G**VMT**AY**RHALSNVQ**-..-----**L**Y**GPTNF**.**A**PI**IE**N**V**A**RK**....A**QN**MIH**DS**AR-----.--Y**QILLIITDG**II**S**--.....--DMH**AT**.**IRS**II**SAS**GL**P**...**L**S**II**I**IGVGN**E.....**DF**E**K**M**HEL**D**S**D**DA**LL-----.---QQDSRIAQ**RDIVQFV**T

P70960/1-189 P**A**N.**VA**V**LLD**..**AS**G**SMA**KR...IDGV**SKFN**S**AKK**E**I**.S**K**FA**SSLP**........**EGTQV**KMSV**FGS**....EG**NN**KNS**GK**-.......**VQS**CEA**IRNV**YGFQ**S**F(10)TIGP**TG**W**TPI**.**AKALNEAKS**S....-**FD**QLD**A**KGE-----.---**KVV**Y**LLTDGE**E**T**CG.....----G**N**P.**IKTAKELQK**D**N**...**I**T**V**N**VIG**F**D**YKE...GY**KGQLN**A**IA**KV**GG**GEYFPAY.------**T**Q**K**D**VEKIFTQ**QS

O43981/1-173 L**LD**.**VMLVVD**..E**S**G**SIG**........T**SNFRKVRQFI**.E**D**F**VNSMP**IS.....P**EDVRVGLITFAT**.....**RSKVRWNL**SD....PK**ATN**P**S**LA**ISA**A**RSLS**Y..-----S**TGVT**Y**T**.HY**GLQ**D**AKKL**....L**YD**T**N**A**GAR**N-----.NVP**KLVLV**M**TDG**A**SN**--.....--LPSQ**T**.**RSSA**AA**LRD**A**G**...AI**V**V**VLGVGSG**....V**N**S**SE**C**RSIAG**C**ST**SN-----.--------**C**PRYL**Q**S**NW**SN

Q51665/1-180 N**L**A.**V**S**ILLD**..**VS**R**S**TESA...VTGR**A**V**IEI**E**REAL**.AA**L**AWG**LD**A.......**CGDR**F**AI**NA**FSS**L..KR**D**RV**FL**S**A**CKD....FDEPMG**A**A**IER**R**I**AG**LR**P..------RFY**T**R**L**.**G**A**GIRHA**SA-....G**LS**A**Q**A**SSR**R-----.----**LLLVITDGKPN**DLDHYEGRHGIE**D**S.AM**AVREARR**A**G**...HA**VHGI**T**VD**R**D**.....**AKSW**FP**RI**F**G**Q**GG**F------.--------**S**L**I**P**H**PD**RLL**A

O88493_4/1-177 KR**D**.**ILFL**F**D**..**GS**VNV-........L**GQF**PA**VRDFL**.Y**R**F**IEELD**VK.....P**DGTRVAI**A**QFSD**.....**DVRLE**SR**F**S......E**HQTKAEILNLVKK**M**K**L..----K**TG**K**A**L**NL**.**G**Y**ALDYALR**N....I**F**VR**S**A**GSR**IED---.NVQ**Q**F**LVLL**VA**GRSS**D-.....-----**AV**.AG**PASSLKQ**R**G**...**V**VP**F**IFQAK**NA**.....**N**P**SELEQI**VLS**PA**F------.ILAAES**L**P**K**I**GD**L**Q**S**QIV**S

O75578/1-191 Y**MD**.**VVIVLD**..**GS**N**SI**-........-YP**WSEVQTFL**.R**RLV**G**KL**FID.....P**E**QI**QVGLVQYG**E.....**SP**V**HEWSL**G......D**FRTK**E**EV**V**RA**A**KNLS**R..----RE**G**RE**T**K**T**.**AQAI**MV**A**C**TE**....G**FS**Q**S**H**GGR**PEA---.--A**RLLVVVTDGES**HDG.....-----EE.**L**P**AA**L**KACE**A**G**..R**V**TR**YGIAV**L**G**HY(8)SF**L**R**EIRTIAS**D**PD**ERFFFN-.VTDEAA**L**T**D**I**VD**A**LGDRI**F

G4104232_3/1-17 S**AD**.**IIFLID**..**GS**QNT**G**........N**ANFD**VI**RDFL**.V**NVLERLS**VG.....N**Q**Q**VQVGVVQYS**E.....**EP**I**TMFSL**N......S**Y**P**SKA**A**VLDAVK**G**LS**L..----V**GG**E**S**A**NI**.**GQALDFV**V**E**N....H**FT**RAG**GSR**VEEG--.-VP**QVLVLI**SA**G**P**SS**D-.....-----E**I**.**RDSV**VA**LKQ**A**S**...**V**FS**FGLG**AQ**AA**.....**SRAELQHIAT**D**DS**LVF----.------**T**VPE**FRSFGDL**QE

O26655/1-176 ---.---M**VD**..I**S**G**SM**F.......SDR**K**AA**RVK**G**LI**.E**R**F**IED**AQR.......**H**R**DRISVV**G**F**R**G**....R**DARVIIP**S-.......**T**AHA**SS**F**RDAVESIR**V..------**GGTTPM**.**AQGIQRGLEI**....L**RE**E**K**RH**GE**Y-----.--VPFM**VIL**S**DG**M**PN**VGT...GRDPKRE**A**.V**EAASRLRE**EE...**I**PST**VI**NFER**G**S..RG**GRDL**NM**EIA**LA**SG**GSY----.-YDLHD**L**R**D**PSGAV**AKIM**E

O62171/1-185 W**LD**.**VV**A**VVD**..**DS**KGV**T**........Q**G**G**L**YNIIG**TL**.N**SVLQ**LSKIGTRKDNP**RTTRLGLVSY**NY.....**N**E**TIE**A**TL**N.......DYQ**F**L**D**TN**KM**M**DQLK**I..----SS**N**I**GS**Y**L**.**VTGLS**G**AEQL**....-**FK**Q**D**KEH**S**REH---.-YR**KVVIV**FASTYK**D**D-.....--GAN**D**P.**K**P**VA**A**RF**L**S**S**G**...GI**IITVA**Y**S**Q**S**....H**D**TQ**LLEKLGG**I**AT**KEFNF--.---TNDDPGL**IEKI**Q**D**S**L**L

O83731/1-179 P**VD**.**IFL**M**ID**..K**S**R**SM**QE......PGKFS**SL**H**R**W**V**R.D**E**F**VSSMT**I.......**QGD**W**I**T**V**Y**QF**YE.....**KPE**E**LITL**TL....RSE**QDRDKIISVVDSI**VP..-----N**G**RY**TDI**.**GRALD**T**VWEI**....-**QE**K**R**K**DNN**R-----.--H**KVLLLVTD**L**E**H**D**APLT.SKYRGKQRS.**FQSP**YL**VR**ARR...**V**KHD**N**WYEI**T**L.....**DMA**VH**DRVA**HT**A**REL-----.------**Y**R**S**I**A**AA**H**S**KR**PT

CAMA_CHICK_1/1- P**TD**.**LVFIID**..**SS**R**S**VR........PQ**EFEKVK**V**FL**.S**RVIE**G**LD**VG.....P**NSTRVGVINYAS**.....A**VK**N**EFSL**K......T**HQTKAELLQAVQRIE**P..-----**LSTGT**M**T**.**G**L**AIQFAISR**....A**FS**D**T**E**GAR**L---RSPNIN**KV**A**IVVTDGRP**QD-.....-----**GV**.**QDVS**A**RARQ**A**G**...**I**E**IFAIGVG**R**V**.....**DM**HT**LRQIAS**E**P**LDDH----.VDYVES**Y**SVIE**K**LT**HKF**QE

Q06556/1-186 G**A**E.**VI**V**LLD**..**TS**QK**MT**........E**TDFN**T**AKE**N**I**.K**KLVTTLT**GT(10)YN**N**R**NSV**R**LIDF**YRK..VG**EST**D**L**S**GW**-.......--**D**AK**KI**D**E**K**LNEV**WKK.AKDDYN**G**W**G**V**DL**.QG**AIHKAREI**....-**FN**L**D**KEK**R**SGK---.--R**Q**H**IVL**FSQ**GEST**FS.....-YDIK**D**K.S**K**M**DK**V**A**V**E**E-...----**PV**TY**SN**-.....**PL**FPWPFYF**D**T**TT**RTHN---.------**V**V**N**D**AKKLIDFL**N

O76057/1-188 EKN.**VVFVID**..**VS**S**SM**F........G**TKMEQTKTA**M.NV**ILSDL**Q........A**ND**YFN**IISFSD**.....**TVNVWKA**GGSI..QAT**IQN**VH**S**A**KDYLHC**M**E**-..-----**ADG**W**TDV**.**NSAL**LA**A**A**S**V....LN**H**S**N**QE**P**GRGPSVGR--IP**LII**F**LTDGEPT**AG.....VTTPS**VI**.**LSNVRQA**LGHR...**V**S**LFSLA**F**GDD**....A**DFTLLRRLS**LE**N**RGIARRI-.---YED**T**DAA**LQ**L**KG**L**Y**EE

Q21916/1-195 NR**D**.**IFLIV**N..**G**LANV**G**.......SQ**ANYQK**EI**NFI**AN**QL**TP**TWN**VG.....L**D**K**VRV**M**L**NLQT**D**I...DY**A**I**VW**S**A**DD......VP**SN**A**N**VT**QEVLN**M**LE**Y..----VP**DVTTD**N.**N**MD**LE**CL**FRY**....A**FD**GLD**DSK**EFDERYG-IE**KVVII**FVAA**NAN**--.....--NDQ**DY**.N**ES**F**E**F**AHK**IR(4)AK**VIVVG**M**GSG**....L**DQTRLSKLA**YA**SG**YAFFS--.-TSYDN**L**S**S**L**I**P**KI**N**N**A**I**C

Q44926/1-165 G**M**N.**LHLLVD**..**NS**L**SMS**L.....GDK**VNKK**D**VQDLL**.V**SI**FA**HMA**F......F**NNDKIGV**TF**FSS**.....G**TD**K**FIP**--.......S**S**KGH**S**H**L**G**LI**L**S**E**T**I..------**N**R**N**LKP.**GSSL**A**Y**I**FK**N....----**T**AEYYK-----.-KR**SLVIII**S**D**F**KAN**--.....-----**AY**.**FKS**L**N**V**LSK**R-...HN**VIAI**R**ISD**FF..DE**NFPKI**G**TL**I**C**E**D**IETG----.------ENFL**VS**G**F**S**K**S**T**L

Q91900/1-206 L**M**N.**IFIVLD**..**TS**K**S**V**G**........Q**NRFDEAKSA**S.ILF**IEKMS**NY.....DIK**PR**YC**IISYAS**.....**KA**ISVV**SL**RD....PDS**NN**A**D**A**VMEHLEE**F**Q**Y..-DRHEDKQ**GTNT**.RA**ALH**AI**YEH**....L**IE**Q**E**L**A**Y**E**REGK(5)KIH**NVILL**M**TDGK**F**N**MG(4)EEMKLIKR**F**.**LDVG**I**R**K**DN**PREEY**L**D**VYV**F**GLGSD**....I**DQPEINDLAS**KKEKE-----.VHTFHLQNVEKM**KEF**F**EL**M

YFBK_ECOLI/1-17 ASN.**LVFLID**..**TS**G**SM**I.......SDE**RL**P**L**I**QS**S**L**.KL**LVKEL**R........**E**Q**DNIAIVTYAG**.....**DSRI**A**LP**SI......S**GS**H**KAEI**NA**AIDSLD**-..-----**A**E**GSTNG**.**G**A**GLELAYQQ**....---A**T**K**G**FIKGG---.--I**N**R**ILL**A**TDGD**F**N**VG.....IDDPK**SI**.**ES**M**VKK**Q**RE**S**G**...**V**T**L**S**T**F**GVGNS**N...Y**NEAM**MV**RIAD**V**GN**GN-----.-YSYID**T**L**S**E**AQK**V**LN**SEM

CGHU1A/1-178 P**ID**.**LLFVLD**..**SS**E**SIG**........LQ**NFEIAKDFV**.V**KVIDRLS**RD(4)FEPGQ**S**YA**GVVQYS**H....S**Q**M**Q**E**H**V**SL**RS....PS**IRN**VQ**ELKEAIKSLQ**W..-----**MAGGT**F**T**.**GEALQYTRDQ**....----LL**PPS**PNN---.---**RI**A**LVITDGRSD**TQ.....-----RD.T**TP**L**N**V**LCS**P**G**...**I**Q**V**V**SVGI**K**DV**FDFIP**G**S**DQLN**V**ISC**Q**G**LAP-----.--------**S**Q**GR**PG**LSLV**K

CFAB_MOUSE/1-20 S**M**N.**IYLVLD**..**GS**D**SIG**........S**SNFT**G**AKRCL**.T**NLIEK**V**A**SY.....GVR**PR**Y**GLLTYAT**.....V**PKVL**VRVSD....ERS**SD**A**D**W**V**T**E**K**LNQIS**YE.--DHK**L**K**SGTNT**.K**RALQ**A**VYSM**....-**MS**WAG**DA**PPEG--WNRTR**HVIII**M**TDG**LH**N**MG(4)TVIQDIR**AL**.**LD**I**GRD**P**KN**PREDY**L**D**VYV**F**GVGP**L....V**D**S**V**N**IN**A**LAS**KK**D**NEHHVFK.VKDMED**L**E**N**V**F**Y**QMIDET**K

O81340/1-169 ---.-**MI**C**ID**..**NS**EW**M**RNG...DYTA**NRFQ**AQA**DAV**.NL**I**CGA**KT**QS.....NPE**NTVGILT**M**AG**....KG**VRVL**V**T**P-.......**TSDLGKIL**A**C**M**H**G**LD**-..-----**MGG**EM**NL**.**A**A**GIQIAQ**LA....L**KH**R**Q**NKK**Q**------.--Q**Q**R**IIV**FA-**GSPV**K-.....-YDKK**VL**.**ET**I**GRKLKK**N**S**...**V**A**L**D**VV**DF**G**E**D**.....ED**GK**S**EKL**E**A**L**VA**AV-----.------NN**N**D**TSHIVH**VPA

E1361269/1-180 ---.-**MILID**..**NS**EW**M**ING...DYIP**TRFE**AQ**KDTV**.HM**I**F**NQK**IND.....NPE**N**MC**GL**M**T**I**GD**....N**SPQVL**S**TL**-.......**TRDYGK**F**LSA**M**HDL**PV..------R**GN**AK**F**.**GDGIQIAQ**LA....L**KH**R**E**NKI**Q**R-----.---**Q**R**IV**AFV-**GSP**IV-.....-EDEK**NL**.**IR**L**AKRMKK**N**N**...**V**A**I**DI**I**H**IG**ELQ...NES**AL**Q**H**F**I**D**A**A**NS**SDSCH--.LVSIPPSP**Q**L**LSDLVNQ**SP

ITH2_MOUSE/1-18 PKN.**ILFVID**..**VS**G**SM**W........GI**KMKQT**V**EA**M.K**TILDDL**R........**TDDQ**F**SVVDF**NH.....**NVRTWRN**DLVS...ATK**T**Q**IAD**A**KRYIEKIQ**P..------**SGGTNI**.**NEAL**L**RAI**F**I**....LN**E**A**S**NM**G**LLNP---.DSV**SLIILV**S**DGDPT**VG.....ELKLSK**I**.**QKNVKQ**SI**Q**D**N**...**I**S**LFSLGIG**F**D**....V**DYDFLKRLSN**E**N**RGIAQR--.IYGNQD**T**S**S**Q**LKKFYNQV**S

CA36_CHICK_4/1- KK**D**.**IVFLID**..**GS**TA**LG**........T**G**P**FN**SI**RDFV**.A**KIVQRL**EVG.....P**D**LI**QVAV**A**QYAD**.....**TVR**P**EF**Y**F**N......T**HQNR**K**DVM**AN**VKK**M**K**L..----M**GGTA**L**NT**.**GSALDFVRN**N....F**FT**SAA**GCR**MEEG--.-VLPM**LVLIT**G**GKS**MD-.....-----**AV**.**EQAA**A**EMKR**NR...**I**V**ILAVG**SR**NA**.....**D**V**AELQEIA**HER**D**FVF----.------QP**N**D**FR**L**QF**M**QA**I

O60310/1-190 PKR.**L**R**LVVD**..**VS**G**SM**YRF...NRMD**GRLERT**M**EAV**.CM**V**M**E**A**F**ENY.....E**E**KF**Q**YD**IV**G**HSG**D...GY**N**I**GL**V**PM**NK.....IP**KD**NK**Q**R**LEILKT**M**H**A..-HSQF**C**M**SG**DH**T**.L**EG**T**EHAIKE**....-**I**VK**E**E**ADE**Y-----.----F**VIVL**S**D**A**N**L**S**R-.....-YGIH**PA**.**K**F**A**QIL**TRD**PQ...**V**NA**FAI**F**IGS**-.....LG**DQ**A**TRL**QRTL**P**AGRSFV-.AMDTKD**I**P**Q**I**LQQIFT**S**T**M

ITAX_HUMAN/1-18 EQ**D**.**IVFLID**..**GS**G**SIS**........SR**NF**ATMM**NFV**.RA**VISQF**QR.......P**STQ**F**SL**M**QFSN**.....**K**F**QTHFTF**E......E**FR**RT**SN**P**LSLL**A**SVH**Q..-----**L**Q**G**F**T**Y**T**.**ATAIQ**N**V**V**HR**....L**FH**A**S**Y**GAR**RDA---.--T**KILIVITDGK**KEG-.....--DSL**DY**.**KDV**IPM**AD**AA**G**...**I**IR**YAIGVG**L**A**FQNRN**SW**K**ELNDIAS**K**PS**QEHIFK-.----VEDF**D**A**LKDI**Q**NQL**K

P74074/1-191 QR**D**.Y**TLIID**..K**S**G**SMS**IIE.PKFQK**SRWELVQE**ST.LA**L**A**RK**C**D**Q......L**DAN**G**I**T**V**Y**TFSG**.....**K**F**R**R**Y**D**N**V-.......--**N**A**SKVEQI**F**QE**N**E**P..------**VGGTNL**.**T**AV**LQ**D**ALN**N...FL**QR**K**K**S**N**QAPTG---.---**E**T**ILVITDGEPN**DRR...SVFEIIIQ.A**S**K**C**L**DADE**E-...**L**A**I**SFMQ**IGND**P...S**A**TK**FLQ**A**L**D**D**QLMEVGAKFD.IVDTVT**F**D**E**ME**D**LT**LTEV**L

Q27591/1-213 P**LD**.**LY**V**L**M**D**..LTWT**M**RD......DKK**TLEE**LGAQ**L**.S**Q**T**LKNLT**........G**N**Y**RLG**FG**SFAD**.....**KPTL**P**M**I**L**PQ(23)SL**TDDIP**AFT**SAV**A**N**S**K**I..-----**TGN**LD**NL**.EG**GLD**AL**MQ**V...IVC**T**K**E**I**G**W**K**EQA---.--R**KVVILVTDG**FMH--.....--LAG**DG**.**L**L**AG**IIQ**RN**DKQCH**L**NKA**G**EYT**GS**LN...Y**DYP**S**LEEI**YRELLRRKINVI.FAVTEE**V**V**S**S**Y**W**EL**SA**LM**K

P76396/1-175 R**C**P.C**ILLLD**..**VS**G**SMN**........GRP**INE**L**N**A**GL**.V**T**FR**DEL**LADP..LAL**K**R**VELGIVTFG**-.....P**VHVEQPF**-.......-**TS**A**AN**F**F**PP**I**L----..----F**A**Q**GDTPM**.**G**A**AITKALDM**....V**EE**R**K**REY**R**ANG-IS.YYRPW**I**F**LITDG**A**PT**D-.....-EWQA**AA**.N**KV**F**R**GE**ED**KR...FAF**FSIGV**Q**GA**.....**DM**KT**L**A**QISV**RQ**P**L------.----PLQGLQ**FRELFSWL**S

VWF_HUMAN/1-175 P**LD**.**VILLLD**..**GS**S**SFP**........A**S**Y**FDE**M**KSF**A.KAF**ISK**A**N**IG.....P**R**L**TQVSVLQYGS**.....I**TTI**DV**PW**N......V**V**PE**KAHLLSLVD**VM**Q**R..-----E**GGPS**Q**I**.**GDAL**G**FA**V**RY**....LT**S**EMH**GAR**PGA---.-SKA**VVILVTD**V**SVD**--.....-----**SV**.DA**AAD**A**ARS**NR...**V**T**VFPIGIGD**R....Y**D**A**AQLR**I**LAG**P**AG**D------.-SNVVK**L**Q**R**IE**D**LP**T**MV**T**L

CA36_HUMAN_4/1- A**AD**.**IVFLID**..**SS**EGVR........P**D**G**F**A**H**I**RDFV**.S**RIVRRLN**IG.....P**S**K**VRVGVVQFSN**.....**DV**FP**EF**Y**L**K......T**YRSQA**P**VLDAIRRLR**L..----R**GGSP**L**NT**.**GKALEFV**A**R**N....L**F**VK**S**A**GSR**IEDG--.-VP**Q**H**LVLV**LG**GKS**QD-.....-----**DV**.S**R**F**AQ**V**IRS**S**G**...--**I**V**SLGVGD**RN...I**DRTELQTI**T**N**D**P**RLVF----.------**T**V**R**E**FREL**P**NI**EE

O88493_7/1-180 P**T**E.**LAF**A**LD**..**TS**EGV**T**........Q**DTFSR**M**REVL**.LG**IV**G**DLT**IAES.NCP**RGARVAVVTY**N**N**.....**EVTTEI**R**F**A......SS**K**K**KS**A**LLD**S**IQNLQ**V..---AL**TS**KQQ**SL**.E**TA**M**SFV**A**R**N....T**FK**RVR**SG**FPMR---.---**KV**A**V**FFS-N**KPT**R-.....--ASPQ**L**.**REAV**L**KLSD**A**G**...**I**TP**L**F**L**TSQE-.....**DR**Q**LIN**A**L**QIN**NT**AVGHAL-.------**V**LPAR**RDLTDFL**K

G3982897/1-206 K**L**N.**IYI**AM**D**..I**S**D**SIA**........EE**DFN**S**ARNAV**.K**KLITK**V**S**SF.....SV**SPN**YE**II**F**FAS**.....**DV**LEVV**NI**IDFS.GDKR**KPLVDVL**AE**LNN**F**K**Y..--DARD**NVGTNL**.**N**L**A**F**K**TI**LER**....-**M**AI**Q**KKR**N**EML-FM.EIH**HVLI**FF**TDG**AY**N**MG(5)TMAKIRE**SV**.**Y**M**NNKT**K**RE**KY...**L**D**VYV**F**GVGSD**I...F**DEDI**MPL**V**TKR**NG**ERHYFK-.LKNVID**L**E**R**T**FDDIIDE**SE

CAMA_HUMAN/1-18 A**TD**.**LVFLID**..**GS**K**S**VR........PE**NFELVKKFI**.S**QIVDTLD**VS.....D**K**L**AQVGLVQYSS**.....**SVR**Q**EFPL**G......R**FHTK**K**DIK**A**AVRN**M**S**Y..-----**M**EK**GT**M**T**.**G**A**ALKY**L**ID**N....S**FT**V**S**S**GAR**PGA---.--Q**KV**G**IV**F**TDGRS**QD-.....-----Y**I**.N**DAAKKAKD**L**G**...FKM**FAVGVGNA**.....**VEDELREIAS**E**PV**AEHYFY-.TADFKT**I**N**Q**I**GKKL**Q**KKI**C

YT15_MYCTU/1-18 Q**A**A.**VALLVD**..**TS**F**SMV**.......ME**NRW**LPM**KRT**A.LA**L**H**H**LV**C**TR.....F**RSD**A**L**Q**II**A**FG**R.....Y**ART**VT**A**--.......-----AE**LTGL**AG**V**--..-----**Y**EQ**GTNL**.H**HAL**A**LA**G**RH**....-----LRR**H**AGA---.--QP**VVLVVTDGEPT**AH(17)HPRTIAH**T**.V**RG**F**DDM**A**R**L**G**...AQ**V**TIFR**LGSD**P...GLAR**FIDQVA**RR**V**QGRVV---.---VPD**L**DGL**G**AAV**V**GD**Y**L

VWF_HUMAN_2/1-1 L**LD**.**LVFLLD**..**GS**SR**LS**........E**AEFE**VL**K**A**FV**.V**D**MM**ERL**RIS.....Q**K**W**VRVAVVEY**H**D**.....G**SHAYIGL**K......DR**K**RP**SELRRI**A**SQVK**Y..----A**GS**Q**V**A**ST**.**SE**V**LKYTL**F**Q**....I**FS**KIDR**PE**A-----.-SRIA**LLL**MASQ**EP**QR-.....--MSR**NF**.V**R**Y**VQ**G**LKK**KK...**V**I**VIPVGIGP**H....A**NL**K**QIR**L**I**EKQ**AP**ENKAFV-.LSSVDE**L**E**Q**QR**DEIVSYL**C

E1390784/1-162 I**LD**.**VVFVL**E..**GS**DK**IG**........E**GNFNK**I**KEF**M.K**QVIQRMD**VS.....Q**ES**I**HISIIQYS**Y.....**TVTVEFSF**N......E**TQSK**RY**ILD**R**IEQIH**-..---HR**GGN**R**TNT**.**GKALEY**LS**E**N....T**FS**S**S**Q**GNR**KTA---.--P**HLV**YM**V**V---**SN**--.....-----**PA**.T**D**EI**KRL**P**Q**D-...**I**Q**VIPIGVGPN**....A**DI**H**ELE**M**LS**RP**NA**PI-----.------------L**I**N**DF**DR

Q31430/1-197 D**T**H.**IYLVID**..**AS**Y**S**V**G**........KE**DFD**T**G**L**NFV**.K**DLINRIG**MY.....V**RN**I**R**Y**SIV**M**YAT**.....**NPSLKLS**VRD....SWS**ND**P**N**A**VIKILDDLD**Y..-YEFDD**TPGTNT**.**A**M**ALKMVLD**TM.ALY**K**VA**N**Q**NT**FKDI---.--R**Q**A**IILLTDGRSN**VG.....-PPPGK**F**.**L**M**DN**I**DLD**IPKE.HMD**VYV**F**G**M**GDV**.....Y**KDEIETIAS**QK**P**NEQHSFI.LRDYDD**L**N**E**V**FEKMLH**ADE

G3928787/1-188 G**MD**.**IYF**AF**D**..**AS**N**S**V**G**........LK**NFEIGKTF**A.K**QLV**G**KL**QVNT...SPG**GTRVG**A**VSYSS**.....**EAR**R**LFN**VN......D**FTS**T**VDV**V**KAIE**ANVN..----Y**TN**K**GTNL**.**P**A**ALE**TIGV**M**....-**IT**E**T**A**D**E**S**GYSSR-.--K**RIL**F**IITDG**F**SN**VG.....----G**A**P.S**KSAQ**P**LKE**D**A**..A**L**K**IHCIGIS**R**N**....T**DKT**A**L**A**EIAS**P**PV**SEHVFY-.LSDYNE**L**E**R**A**VE**A**ITS**TNR

CA1C_MOUSE_1/1- E**AD**.**IVLLVD**..**GS**W**SIG**........R**ANFR**T**VRSFI**.S**RIVE**V**F**EIG.....P**K**R**VQIAL**A**QYSG**.....**DPRTEW**Q**L**N......A**HRDK**K**SLLQAV**A**NL**PN..-----K**GGNT**L**T**.**G**M**ALNF**I**RQQ**....S**FK**T**Q**A**G**M**R**PRA---.--R**KI**G**VLITDGKS**QD-.....-----**DV**.**E**A**PSKKLKD**E**G**...**V**E**LFAIGI**K**NA**.....**DEVELK**M**IAT**D**PD**DTHAYN-.VADFES**L**S**K**I**VDDLT**IN**L**C

Q23563/1-190 W**LD**.**VV**A**VVD**..**NS**AG**MT**........K**G**G**LT**S**V**AAN**I**.A**SI**F**SK**N**T**QI(4)TSP**KTTRLALVTY**N**V**.....**DATT**AA**DL**N......K**FQSIDDIYSGINS**ALA..--TISS**S**EE**S**Y**L**.**ARGLSQAEK**V....F**Q**AG**K**H**G**F**N**RAH---.-YQ**KVVIV**YASTYK**G**S-.....--GDL**N**P.VP**VAQRLKT**S**G**...**V**T**IITVA**Y**D**Q**N**KD.GDI**LV**D**LEKIAT**PYHNL-----.---SNENL**N**V**I**G**EI**QG**FL**L

ITA2_HUMAN/1-19 L**ID**.**VV**V**V**C**D**..E**S**N**SI**-........-YP**WD**A**VKNFL**.E**K**F**VQ**G**LD**IG.....P**T**K**TQVGLIQYAN**.....**NPRV**V**FNL**N......T**YKTK**E**E**M**I**V**A**T**SQ**T**S**Q..----Y**GGD**L**TNT**.FG**AIQYARKY**....A**YS**AAS**GGR**RSA---.--T**KV**M**VVVTDGES**HDG.....-----**SM**.**LKAV**I**D**Q**CN**H**D**..N**I**LR**FGIAV**L**G**YL(8)NL**I**K**EIK**A**IAS**I**PT**ERYFFN-.VSDEAA**L**L**E**K**A**G**TLGEQI**F

JC5576/1-185 PKN.**IVFVID**..I**S**G**SMA**........GR**KIQQTR**V**AL**.L**KILDDM**K........**QDD**Y**L**NF**I**L**FST**.....G**VTTWKD**SLVQ...ATPA**NL**E**E**A**RTFVRSIS**D..------Q**G**M**TNI**.**NDGL**L**RGIRM**....LT**D**A**R**EQH**T**VPE---.RST**SIII**M**LTDGDAN**TG.....ESRPEK**I**.**QENVRKA**I**E**GR...FP**LYNLG**F**GNN**....L**NYNFLET**M**A**LE**N**HGVARRI-.---YEDSDAN**LQ**L**QG**F**Y**EE

G4104232_2/1-17 A**AD**.**IVFLVD**..**SS**W**S**A**G**........K**DRF**L**LVQEFL**.S**DVVESLA**VG.....D**ND**F**H**F**ALVR**LN**G**.....**NPHTEF**L**L**N......T**YHSK**Q**EVLSHI**A**N**M**S**Y..-----**IGGS**NQ**T**.**GKGLEYVIH**S....H**LT**EAS**GSR**AADG--.-VP**QVIVVLTDGQS**ED-.....-----**GF**.AL**PS**A**ELKS**A**D**...**V**N**VFAVGV**E**GA**.....**DE**RA**L**G**EVAS**E**P**LSMHVFN-.---LEN**V**T**S**L**H**GLV**GNLV**S

Q53065/1-169 R**V**R.**LLLI**A**D**..**VS**L**S**V-........----**R**PI**T**A**F**T.L**RL**A**Q**A**M**HR.......**RADR**CE**VL**A**F**V**D**.....**RP**VDVT**D**TL......L**ASS**G**D**GA**L**A**AV**LAHP-..--GLD**L**E**ASSDY**.**GR**V**LTE**L**LDE**....-----H**GNS**L-----.NSR**T**S**VIIV**G**DGRCN**--.....--GLP**P**Q.V**D**KL**EELRR**K**V**...HR**L**AW**I**TPE**P**-.....-**Q**R**Y**W**NQ**A**SC**AM**P**E-YSEI-.CDEVVV**A**R**D**A**AQ**L**MAK**A**A**E

BCHD_CHLVI/1-18 G**T**L.F**IF**M**VD**..**AS**G**SMA**........L**NRMRQAK**G**AV**.A**SLLQN**AYV.......**H**R**DQVSLISF**R**G**....K**QAQVLLP**P-.......S**QS**V**DR**A**KR**E**LD**V**L**P-..-----**TGGGTPL**.**ASAL**LT**GWE**T....---A**K**Q**A**R**T**KGI---.-TQIMF**V**M**ITDGRGN**IP(13)KEELEKE**V**.**E**AL**A**L**SIQS**D**G**...**I**AS**IVV**DTQM**N**Y...L**SRGE**AP**KLA**QKL**G**GRYFY--.------**L**P**N**AKA**EQI**V**EA**A

Q19346/1-192 PKR.**L**KVC**LD**..**VS**G**SM**YRF...NGYDQ**RL**V**KS**L**EA**A.LMTM**T**A**LD**GK.....T**D**K**VQ**YD**II**G**HSG**.....**DS**P**C**VSFVKT(4)KNNK**E**R**LDTLKRMI**A**H**T**Q**Y..-----**CVSG**D**NT**.**VESLQFAIKE**....LAAK**K**D**D**F**D**E-----.---**TVVILV**S**D**A**N**LER-.....-YGIQ**P**K.**E**LK**D**AM**AKE**P**N**...**I**NS**FVI**F**IGS**-.....LS**DE**A**DQL**QREL**P**VGKAFV-.LKDTSE**L**P**K**I**METIFS**S**T**I

ITH1_PIG/1-190 NKN.**VVFVID**..I**S**S**SM**E........GQ**K**V**KQTKEAL**.L**KILSDL**K........P**GD**YFD**LV**L**FGS**.....A**VQ**S**WRG**SLVQ...AS**T**A**NLD**AA**RSYVRQ**F**S**-..-----**LAGSTNL**.**N**G**GL**L**RGIEI**....LN**K**A**Q**G**S**LPEFSNRA.---**SILI**M**LTDGEPT**EG.....VTDRSQ**I**.**LKNVRDA**I**R**GR...FP**LYNLG**F**G**H**D**V..EW**NF**L**EVR**A**L**E**N**N**G**RAQRIYED.-HDSAQQL**Q**G**F**Y**DQV**ANPL

O52840/1-167 P**A**P.**IV**A**LLD**..I**S**G**SM**-........---N**EYTR**L**FL**.HF**L**HAIG**D**........ARK**RVSV**FL**FGT**.....**R**L**T**NVTRAL......RQ**RD**P**DE**A**L**ASC**S**ASVE..----D**WAGGT**R**I**.**S**A**SLH**NFN**KL**....---WARR**V**LSQG---.---A**IVLLI**S**DG**LERE-.....--ADSR**L**.AFEM**DRLHR**S**C**...RR**LI**W**L**NPLLRF..GGF**EAK**A**Q**G**I**KMML**P**HV-----.-----DEF**R**P**VHNL**S**SI**QE

CA36_CHICK_11/1 D**LD**.**VIL**GF**D**..**VS**DVG**A**GQN.IFNSQRG**LE**SRV**EAV**.L**N**R**ITQM**QKI(4)SRAP**SVRVAI**MAQ**S**R....GG**P**VEG**LDF**-.......S**E**Y**QPELFE**RF**Q**GM**R**T..-----R**GP**YFL**T**.**AE**T**LK**SY**QNK**....-----FR**S**APSG---.-ST**KVVI**HF**TDGTDD**--.....--YLDQ**M**.**KTAS**A**DLRR**Q**G**..VHA**LL**F**VGLD**R**V**....K**NF**E**EV**M**QL**EFGR**G**FTYNRPL.RVNLLD**L**DFE**L**A**EQLD**N**I**A

O45444/1-187 W**LD**.**VV**V**VVD**..**NS**KG**MT**........NEG**ITE**IAAN**I**.V**TV**FG**N**G**T**RI(4)SDP**RSTRLGLVTY**N**G**.....**RSTI**VA**DL**N......L**LQSIDDLYQ**S**V**F**S**TLN..--QVSN**SDDS**F**L**.**AKGI**GA**AEN**V....L**QS**G**R**T**NG**VRSN---.-YK**RLVVV**YASAYK**G**E-.....--GEL**D**P.**I**P**VADRLKS**S**G**...**V**V**V**S**TVA**F**D**Q**D**....G**DEALL**AG**L**T**N**I**AS**PNYAF--.------**T**S**K**D**LN**LV**GEL**QG

E1390196/1-200 P**VD**.**LV**T**VID**..**VS**GGN-........---**IEMVKRA**M.R**QVISSL**R........**ETDRLS**M**VSFSS**.....**SSK**R**L**T**PL**RR....MT**ANGR**RLA**RRIVDDIS**G..-----D**GDG**M**SV**.**NDAVKKA**A**K**V....-**IE**D**R**RQK**N**L-----.--F**T**T**I**F**VLTD**R**N**R**N**SA(28)GACNHALP.**EDV**FA**K**RI**K**S-...--**LLSLSV**Q**D**-.....LTLN**L**GL**VSG**S**G**QGKVTSV-.---YSLSG**R**P**V**WLGSG**LI**R

CA36_HUMAN_5/1- EK**D**.**VVFLLD**..**GS**EGV-........R**S**G**F**P**L**L**KEFV**.Q**RVVESLD**VG.....Q**D**R**VRVAVVQYSD**.....**RTR**P**EF**Y**L**N......S**Y**M**NK**Q**DV**V**NAVRQLT**L..----L**GGPT**P**NT**.**G**A**ALEFVLR**N....I**L**VS**S**A**GSR**ITEG--.-VP**QLLIVLT**AD**RSG**D-.....-----**DV**.**RNPS**VV**VKR**G**G**...AVP**IGIGIGNA**.....**DITE**M**QTIS**FI**PD**FAV----.---AIP**T**F**R**Q**L**G**T**VQ**Q**V**I**S

Q60863/1-174 V**LD**.**VVFVL**E..**GS**DEV**G**........E**ANFNKSKEFV**.E**EVIQRMD**VS.....P**DATRISVLQYS**Y.....**TVTMEYAF**N......G**AQSK**E**EVLRHVREIR**Y..----Q**GGN**R**TNT**.**GQALQY**LS**EH**....S**FS**P**S**Q**GDR**VEA---.--P**NLV**YM**VT**-**GNPA**S-.....-------.-**D**EI**KRL**PGD-...**I**Q**V**V**PIGVGP**H....A**NM**Q**ELERIS**RPI**A**PIF----.IRDFET**L**P**R**E**A**P**DLV**L**QT**C

G3925281/1-173 RRV.**VCLVLD**..K**S**G**SMD**.......KE**DRL**I**R**M**NQA**A.ELY**LTQIV**E.......**K**E**S**M**VGLVTF**D**S**.....A**AHIQ**NY**L**I......K**ITS**S**SD**Y**QKI**TA**NL**PQ..----Q**ASGGTSI**.**CHGLQ**A**GFQ**A....-**IT**S**S**DQ**ST**SGS---.----E**IVLLTDGEDN**--.....-----**GI**.**RSC**F**E**A**VSR**S**G**...AI**IHTIALGPS**.....**A**AR**ELETLSD**M**TG**GLRFYA-.---NKD**L**N**S**L**ID**A**F**S**RI**SS

O45445/1-188 W**LD**.**IVFVVD**..**NS**KN**MN**.......LY**N**V**YN**TI**SNL**F.NPF**VQ**IG**T**GYD...DP**RSTRVG**F**ITY**NW.....**NAT**DVA**DF**Y......K**LQSYSDL**S**S**Q**IQ**A**LS**VT.--PLSR**AD**E**T**Y**I**.**DTGLQ**A**AINM**....-**IN**A**T**G**G**L**R**D-----.NYK**KVVVL**F**T**SKY**N**Y--.....--YHTYP.**ED**L**TD**Y**LKS**T**G**...**V**T**IITV**NT**GGD**S..YT**TQSL**R**DKIAS**K**G**MAFA----.MSDGNT**T**A**E**LQ**K**AV**L**A**I**NC

Q52359/1-176 E**A**A.**VAFVLD**..**AS**G**SMS**GQ....FSK**GN**V**Q**S**V**L**D**R**I**.AV**L**AA**QFD**........**DDGE**MD**V**WG**FG**E.....**K**H**K**K**Y**P**N**V-.......--**TLDNL**D**TYIQSIR**G(8)NLPG**LGGT**N**N**E.**P**PVM**EE**IV**DY**....----FK**DSK**I-----.--PVY**VV**F**ITDG**GI**S**--.....--KTR**AI**.**KDA**I**RR**SA**N**Y**P**...**I**FW**K**F**VGLGGS**.....**SYGILKNL**D**D**F**TD**RRV----.------DN**T**H**F**FA**M**D**DFG**S

O06847/1-190 D**L**S.**VAIL**M**D**..**CS**R**S**TEAV...VGER**P**V**IE**T**ARE**R**L**.AA**L**AGG**ID**V.......A**GDRLAI**WG**FSS**.....VR**R**D**R**VF**L**HRCK.GFDEPMGEA**V**TGR**I**GG**LR**P..------**G**HY**T**R**L**.**G**A**AVRHA**SA**M**....---LAAE**GS**S-----.--R**KLLLILTDGKPN**DLDHYEGVHGIE**D**S.**R**M**AVREARS**L**A**...QS**VHAV**V**IDAD**....GQDW**F**A**R**IF**G**RA**G**FTL-----.LPDPAR**L**P**R**A**L**P**DLYR**S**L**T

Q49139/1-178 G**A**Q.**I**S**MLID**..R**S**G**SMN**ETF.AGRQP**S**GA**EESK**A**A**A.S**R**R**I**L**R**DFVG....ERAH**DQ**F**AV**TA**FST**.....A**P**M**L**VV**PM**-.......**TD**R**HD**A**VR**A**AI**AA**ID**R..----P**G**L**D**Y**TNV**.**ARGL**G**MALSQ**....----FG**AG**APGV---.--S**R**A**LLLV**S**DG**A**AV**I-.....-DPRIQ**A**.**Q**LR**AE**F**TK**VQ-...PN**LY**W**L**F**L**---.....RTKGSP**SI**T**D**K**PA**GE-----.-----D**T**P**Q**A**A**P**ERH**LD**L**F

O76430_1/1-172 T**YD**.**VYLIVD**..**VS**NKA**S**........A**ADF**AAM**KTAI**.H**N**F**V**AP**FA**IG.....DL**GTS**F**ALVT**T**G**I....D**S**QL**FF**T**NF**K......N**GQSRGDVLTAVD**G**L**LQ..----DD**VPG**Q**TL**.**N**L**ALS**AI**Q**G**Y**....-----L**A**QPTSA---.-SK**KI**MAYF**T**ST**TA**W--.....--DVS**PI**.S**T**M**NS**LK**SK**Y**S**...**L**SPV**AV**QW**GAS**....A**S**T**S**D**LTNL**V**G**G**AN**CVN----.------**V**V**S**NKAASP**QWL**Q

CA26_CHICK_2/1- P**VD**.**IVFLLD**..**GS**ER**IG**........EQ**NFHRAHHFV**.E**QV**A**QQLT**LARRNDDNM**NARIALLQYGS**E...R**E**Q**NV**V**FPL**-.......**T**Y**NLTEI**S**NAL**A**QIK**Y..-----**LDSSSNI**.**GSAI**I**HAIN**N...IV**LS**PGN**G**Q**R**VARRNA.--ELSF**V**F**ITDG**I**TG**SK.....-----**NL**.**EEA**I**NSMKK**Q**D**...**V**MPT**VVALGSD**....V**DMD**V**L**L**KLG**LG**D**RAAIFR--.---EKD**Y**E**S**LS**Q**PS**F**FDRF

O75131/1-207 Q**L**N.F**T**VG**VD**..FTG**S**N**G**DP(11)NGV**NEY**LT**A**LWS**V**.GL**VIQDYD**........A**D**KMFPAFG**FGA**(4)QW**QVSHEFPM**NFNPSNPY**CNGI**QG**I**V**EA**Y**RS**CLP..--QIK**L**Y**GPTNF**.**S**PI**INHV**A**RF**....AAAA**T**QQQ**T**ASQ---.--YF**VLLIITDG**VI**T**--.....--DLDE**T**.**RQA**IV**NASR**L**P**...MS**II**I**VGVGGA**.....**DFS**AM**E**F**L**D**G**D**GG**SLRSPL-.------**G**EVA**IRDIVQFV**P

Q23565/1-187 W**LD**.**IV**A**VVD**..**NS**KG**MT**.......DK**G**VV**T**V**A**G**QIV**.SLF**VD**G**Q**QLGIDPNQP**RTTRIGIVTY**NR.....**DATV**VA**DL**N......K**ITSIDQL**A**DIV**FGAL-..-HKASSI**ADS**Y**L**.HA**GLE**A**A**D**DL**....L**QR**Q**S**F**ATS**RGH---.-YK**KLVIV**YASEY**SG**T-.....-----**GT**.**QDP**LPL**ATR**MKV.D**V**A**I**A**TVA**Y**G**Q**D**....N**V**N**GFLRQLS**QI**AT**PGYNFT-.------NQ**N**G**IQ**LVP**EL**RA

307094/1-243 K**A**A.**VVL**CM**D**..**V**GFT**MS**NSI..PGIE**S**P**FEQAKKVI**.TMF**VQRQV**FA.....E**N**K**DEIALV**L**FGT**(8)GG**D**QYQN**IT**VHRH...LM**L**P**DFD**L**LEDI**E**SKIQ**P..-----**GS**QQA**DF**.L**DAL**IV**SMD**V....I**QH**E**T**I**G**.**K**KFE---.--K**R**H**I**E**I**F**TD**L**SS**RFS(42)GGHGPSFP.**LKG**I**TE**Q**QK**E**G**LEI**V**KMVM**ISL**E**G**E....D**GLDEI**Y**S**F**S**ESLRKLCVFK-.--------**K**IE**RH**S**IHW**PC

Q55727/1-192 NR**D**.Y**TL**M**ID**..K**S**S**SMA**TAD.DPNGP**TRWEIAQ**AST.IA**L**A**KK**CEE......I**DSD**G**I**T**V**YL**FSG**.....**R**F**R**R**Y**D**N**V-.......--**T**AE**KV**AY**I**YA**N**N**E**P..------M**G**R**TDL**.**A**G**ALK**D**GLD**N....F**FQ**R**R**Q**AGQ**TKPN--.--G**E**TF**LIITDGEPT**DR.....KAVIRL**I**.**LEASQKIDR**DE..E**L**A**I**SL**I**Q**VG**R**D**K...K**A**T**AF**F**Q**A**L**D**D**QLQAAGAKFD.IVDTVT**M**E**D**MQGMS**LS**D**V**L

E1355127_1/1-17 P**LD**.**LVFVID**..**SS**R**S**VR........PF**EFE**TM**RQFL**.MG**LLR**G**LN**VG.....P**NATRVGVIQYSS**.....**QVQ**SV**FPL**R......A**FS**R**R**E**D**M**ERAIRDL**VP..-----**LA**Q**GT**M**T**.**G**L**AIQYAMN**V....A**FS**VAE**GAR**PPEE--.RVP**RV**A**VIVTDGRP**QD-.....-----R**V**.A**EVA**A**QAR**AS**G**...**I**E**IYAVGV**QR**A**.....**D**V**G**S**LR**AM**AS**P**P**LDEHVF--.------**L**V**E**S**FD**L**I**Q**EFG**L

O45441/1-191 W**LD**.**VV**V**VVD**..**NS**AP**MT**........QEG**LTEV**AAQ**I**.V**TV**FGAG**T**RI(4)IDK**RTTRVGLVTY**N**T**.....**EATIQ**A**DL**N......R**FQS**P**DDLFSTV**F**QI**LP..-NDLS**TS**E**D**VF**L**.**AKGI**GA**AEQL**....LAAG**R**K**NNT**RKN---.-YK**Q**M**VIV**YASAY**ND**E-.....--GEE**D**P.**R**PI**AERLK**AS**G**...**V**S**I**A**TVA**F**D**Q**T**....G**DE**E**MIK**L**IG**EI**AT**PGFNF--.---TNEDE**N**L**VKEI**Q**T**A**M**I

O88493_6/1-200 D**ID**.**LAFILD**..**SS**EAT**T**........LF**QFNE**M**KKYI**.GY**VIRQLD**LS(4)ASQ**H**F**ARVAVVQ**Q**ST**(8)VPP**VKVEFSL**-.......-**TDYG**AK**EKLLD**F**LS**RR.--MTQ**L**Q**GT**M**GL**.**GNAIEYTIE**N....I**FE**SAP**NPR**D-----.--L**KI**M**VL**MLT**GD**MQR-.....-QQLEE**A**.**QRA**IL**QAKC**K**G**...YF**L**V**VLGIG**RK....V**NI**K**EV**Y**S**F**AS**E**PN**DVFFKF-.VDKSTE**L**N**E**EPLM**RF**G**RL**L

O74995/1-181 IRH.M**VLVLD**..L**S**N**SM**EER...DFHHK**RFDL**QI**KY**A.S**E**F**V**L**EF**FEQ.....NPI**SQLSII**GVM**D**....GI**AH**R**I**T**DL**-.......**H**G**N**PQ**S**H**IQ**K**LKSLR**D..-----**CSGN**F**SL**.Q**NALEMAR**AS....LS**H**IASH**GT**R-----.---**EVLII**FGSIL**SS**--.....--DPG**DI**.**FKT**I**D**A**L**V**H**D**S**...**I**R**VR**I**VGLAA**-.....EV**AI**C**KEI**C**N**K**TN**S-STKN-.AYGVVISE**Q**H**FRELL**L**E**ST

O43746/1-176 N**VD**.**LVFL**F**D**..**GS**M**SL**Q........P**DEFQK**IL**DF**M.K**DV**M**KKLS**N.......**TS**Y**Q**F**A**A**VQFST**.....**S**Y**KTEFDF**SDY...VK**WKD**P**D**A**LLKHVK**----..----H**M**LLL**TNT**.FG**AINYV**A**TE**....V**FR**E**E**L**GAR**PDA---.--T**KVLIIITDGEAT**D-.....-------.-**SGN**I**DA**A**K**D-...**I**IR**Y**I**IGIG**KHFQTKE**SQ**ET**LHK**F**AS**K**PA**SEFVKI-.---LDT**F**E**K**LK**D**L**FTEL**QK

Q26228/1-206 A**VD**G**ILFLID**..**A**TEG**M**FEEV..DGDT**A**F**MQ**CI**K**A**A**K.S**T**M**LNKIT**S......SPK**D**L**VGII**L**FGT**(8)FK**NV**Y**VLQDL**ES....PG**AES**VL**KLEKLI**A**D**GPKK.FKQEY**G**H**GN**V**NM**.**AD**V**L**WT**C**AL**M**....-**FS**K**S**R**AGQ**R-----.----R**VLVLT**NQ**DDP**HKG...SGDLDDK**A**.VVK**AKDL**L**Q**S**G**...**I**E**L**DL**V**H**L**K**PP**G(4)R**PQ**I**L**Y**KNL**V**T**DKENY-----.---EDG**F**P**E**AS**DKM**E**ELL**L

YIEM_ECOLI/1-16 R**G**P.F**I**VC**VD**..**TS**G**SMG**........GF**NEQ**C**AK**A**F**C.LA**L**M**R**IALA.......**EN**R**R**CY**I**ML**FST**.....**E**IVR**YE**LS-.......--**G**PQG**IEQAIR**F**LS**Q..----Q**F**R**GGTDL**.**AS**CF**R**AI**MER**....-----LQ**SR**EWF---.--DADA**VVI**S**D**FI**A**QR-.....--LPD**DV**.T**S**K**VKELQR**VH..QHRF**HAVA**M**SA**H....GKP**GI**M**R**IFDHIWRFD-----.------**T**GMRS**R**L**L**R**RW**RR

YLS8_CAEEL_1/1- TR**D**.**IIIVVD**..**GS**S**SM**-........-Q**T**S**TYVSQ**E**I**.NM**I**T**K**LTYSW....TL**DDAKV**R**L**ALV**GA**.....YLGN**EFN**GLD.....Y**FTD**S**S**L**IEK**R**LQS**F**R**L..AAMQY**G**LF**S**G**DF**.**NT**T**VRF**LD**ER**....YVGP**R**A**T**FGPRA---.NVQ**K**R**III**FSAH**KGA**S-.....--DIA**ST**.**KS**KL**QEF**A**Q**L**G**Y.A**V**T**I**V**GIGVS**E**D**V..YK**G**TF**Y**H**K**F**VSV**QWFELGVV--.------**A**Q**S**I**IDTITE**D**G**I

3881770/1-188 W**LD**.**VV**V**VVD**..K**S**QL**MT**........N**AQL**W**QVRNTI**.T**QL**FG**S**SRIG(5)ADP**RSTCVGVVTY**D**S**.....**DATT**NAQ**L**D......GP**HSFTDLYNVVQ**GSLN..--NVDS**TNSS**Y**L**.**SKGL**LA**AEQ**A....L**KN**G**R**SR**T**YRFN---.-FL**KVVIV**FAADYQ**G**SG.....--TSN**DA**.**F**PI**ARRMK**YD**N**...TI**IITVA**C**TTN**Q...A**ALD**G**L**A**QISS**PQFSLV----.------DEMN**TSKLVKQL**T

CA36_CHICK_10/1 D**TD**.**IAFI**M**D**..**SS**A**S**T**T**........PL**QFNE**M**KKYI**.S**HLVSNM**EIS(4)ISQ**H**H**ARVAVLQ**Q**AP**(8)FPP**VKTEFSL**T......D**Y**G**SK**E**KIINYLHN**QMT..----Q**L**Y**GT**M**AM**.**GSAVEHT**VA**H**....V**FE**SAP**NPR**D-----.-LKV**IVL**M**IT**-**GK**MEK-.....-QELEY**L**.**REAV**I**DAKC**K**G**...YLFVI**LGIG**K**N**....V**D**VKN**I**Y**SLAS**E**PN**DV-----.------**F**F**K**L**VSK**P**GELH**E

O66676/1-169 E**L**I.FE**LL**M**D**..I**S**S**SM**-........-K**KEEK**IL**NAL**.K**SLI**LVSEVL....DKLKM**E**F**SI**KV**F**NE.....**NV**Y**TLKDF**-.......S**EDY**KVA**K**AR**I**M**DL**LN..----D**LGGSTDL**.**SKAIT**V**G**V**E**S....L**E**VVMKKE**H**K-----.--KG**VLIL**F**TDGQPT**KG.....--LRGEE.**LK**YFI**S**QM**K**MK...**L**P**I**V**AIGVG**E**A**.....**TH**MVK**E**YFDKT**G**LS------.------**V**E**D**IS**K**LPSA**F**SF

SSL1_YEAST/1-17 IRS.**LIL**T**LD**..**CS**EA**M**LEK...DLRP**NRH**A**M**II**QY**A.I**D**F**VHEF**FDQ.....NPI**SQ**M**GII**IMR**N**....GL**AQL**VSQV-.......SG**N**PQ**D**H**IDALKSIR**K..---QEPK**GN**P**SL**.Q**NALEMAR**G**L**....L**L**PVPAH**CT**R-----.---**EVLIV**FGSL**STT**--.....--DPG**DI**.**HQT**I**DSL**V**S**EK...**I**R**VKVLGLSA**-.....QV**AI**C**KEL**CKA**TN**Y------.------**G**D**E**S**F**Y**KIL**LDET

CA1E_CHICK_1/1- I**AD**.**IVILVD**..**GS**W**SIG**........RF**NFRLVR**L**FL**.E**NLVS**A**FN**VG.....S**E**K**TRVGL**A**QYSG**.....**DPRIEW**H**L**N......A**Y**G**TKD**A**VLDAVRNL**PY..-----K**GGNT**L**T**.**G**L**ALTY**I**LE**N....S**FK**P**E**A**GAR**PGV---.--S**KI**G**ILITDGKS**QD-.....-----**DV**.**I**P**PAKNLRD**A**G**...**I**E**LFAIGV**K**NA**.....**DINELKEIAS**E**PD**STHVYN-.VADFNF**M**N**S**I**VE**G**LTR**T**V**C

2828247/1-177 LR**D**.**VLIVVD**..ITR**SMN**VRD..MDGA**SRLD**A**ARDVL**.T**R**W**I**A**SQP**........**CGARVGL**GF**F**TER...R**S**L**TLIEP**VE......I**CTDYATL**AG**TL**AG**LD**W..--RMA**W**E**GDS**M**I**.**SKGLNHALNR**....-----AR**G**LD-----.---AA**LI**F**VTDGQ**E**A**PP.....-----LP.**YSGPD**G**WRE**D**S**...PGGV**VLG**A**GGD**.....QP**A**P**I**P**K**FD**D**L**G**RETGFY--.---GPTDV**Q**H**A**PA**RI**GAPP

YNV4_CAEEL/1-17 MRH.**VMIVID**..**CS**RF**MT**SK...AMPP**SRF**VV**V**M**KAL**.Q**T**F**LDRF**FEQ.....NPI**AQIGLIT**CK**D**....R**KAD**R**L**TM**M**-.......**T**G**NI**RV**LKE**S**LNTLT**-..--EAF**CGGD**F**SL**.Q**NALQLA**CAN....-**LK**GMP**G**HVS-----.--R**EVVLVI**S-AL**ST**I-.....--DPG**NI**.**YST**I**ETMKR**M**N**...**I**RCS**AIGLSA**-.....E**M**FVC**KE**M**A**KA**T**KGE-----.-YSVALDP**D**H**LQ**L**LFSKH**T

D1035689/1-204 K**LD**.**IYI**A**VD**..**AS**D**S**VE........EK**DFDYAKTTI**.KL**LLEKIS**YY.....PV**SPN**YE**IL**M**FAT**.....**DVT**P**II**K**M**NQF..KMQKP**TLTDIFK**EM**DE**F**T**Y..-----DKK**G**DK**T**.**GT**N**I**A**KVYT**V....---I**E**E**S**M**K**IEEL(7)ETQ**HIIIL**FS**DGHAN**MG(4)PKVEQIKR**L**.V**T**K**ND**PK**RE**KK...**L**D**LYV**F**GVGDV**.....**NQD**D**VN**G**L**V**S**QR**D**QEKHFFK.LQDLQE**V**Q**E**M**FDNMIDE**ST

YLS8_CAEEL/1-18 Y**ID**.**IIFVID**..**VS**EG**MG**........Q**G**G**L**M**MVK**AE**I**.N**TLV**G**QMS**LDP..NIQ**K**H**VQVGLIKYSD**.....**KAEV**V**F**KPS......D**YTNEDE**FT**E**D**L**W**S**DPRL.EDVDEK**SD**EV**NL**.HL**GLQQA**A**KM**....----**T**A**S**M**R**NGV---.--R**KVIVV**YAASY**ND**E-.....--GND**DA**.**RQ**I**A**A**NIRE**T**G**...YA**IITVA**F**V**E**P**ES.SNLVM**KI**G**EIAS**PRMNF-----.------**T**SFRD**D**L**LVEQM**E

O88321/1-182 ---.-**M**VC**VD**..**NS**EY**M**RNG...DFLP**TRLQ**AQ**QDAV**.NI**V**C**HSKT**RS.....NPE**NNVGLIT**L**AN**.....**DCEVL**T**TL**-.......**T**P**D**T**GRILS**K**LHTVQ**P..------K**G**KI**TF**.**CTGIR**V**AH**LA....L**KH**R**Q**GK**NH**KMR---.-----**II**AFVGSP**V**ED-.....--NEK**DL**.V**K**L**AKRLKK**EK...**V**N**V**DI**I**NF**G**EEE(4)KLT**AFVNTL**N**G**K**DG**TGSH---.LVTVPP**G**P**S**L**AD**A**LIS**SPI

Q90422/1-207 K**LD**.**IYI**A**VD**..**AS**D**SID**........PK**DFDKAKKII**.K**TLIEKIS**YY.....EV**SPN**YE**IL**M**FAT**.....**DVD**Q**I**VK**M**RDFKTNEK**AR**K**I**L**KIFE**D**LDN**F**N**Y..-----DKK**G**DR**T**.**GT**N**I**A**K**L**Y**L**K**....I**LD**SMSLE**Q**VQNK(6)--Q**HVIIV**F**TDGQAN**MG(4)PKVDLIK**NL**.VIK**NN**AS**RE**NK...**L**D**LYV**F**GVG**K**D**....VK**K**EDM**N**G**L**V**S**EKKDERHFFK.LPDLDE**V**Q**N**T**FD**L**MLD**DST

O45442/1-186 W**LD**.**IVIVVD**..**NS**KG**MT**........NEG**ITEV**AAN**I**.A**T**TFA**S**G**P**RI(4)SDP**RSTRLSILTY**N**S**.....**EATV**VA**DL**N......Q**FQS**A**DDVYQTL**F**S**FLN..--EVSD**SDDS**F**L**.**AKGL**G**MAES**V....L**YN**G**R**M**NG**VREN---.-YN**RLVVV**YASAYREDG.....----E**D**P.**LN**I**AKRLRS**S**G**...**V**A**I**A**TVA**F**DPD**GD.GLL**LSKLSKIAS**P**N**MSF-----.------KS**R**DP**N**LV**GKI**QA

CGHU1A_1/1-199 P**VD**.**LFFVLD**..**TS**E**S**V**A**LRL..KPYG**A**LV**DKVKSF**T.K**R**F**IDNL**RDR(4)DRNL**V**W**N**A**G**A**LHYSD**.....**EVEIIQGL**TR.....MPG**GRD**A**LKS**S**VD**A**VK**Y..-----**FG**K**GT**Y**T**.**DCAIKKGLEQ**....L**L**VGGSHL**K**E-----.--N**K**Y**LIVVTDGHP**LEG....YKEPCG**GL**.**EDAVNEAKH**L**G**...**V**K**VFSVAITPD**....HL**EPRLS**I**IAT**DH**T**YRRNFT-.AADWGQSR**D**AE**E**A**I**S**Q**T**I**D

G3820559/1-191 G**A**L.**IIFVVD**..**AS**G**SMA**........F**NRMS**S**AK**G**AV**.SV**LLNE**AYV.......**N**R**DKVALI**I**F**R**G**....Q**QAETL**V**P**P-.......**TRS**VELA**KK**RF**DQV**PV..------**GGGSPL**.**A**G**AI**A**QAIE**V....---GVN**S**IGSDVG--.--QV**II**T**LITDGRGN**VP(8)NREQLNEE**I**.**L**AL**SR**L**V**P**E**N**G**...FSM**LVI**DT**AN**KF...T**S**T**GF**A**KKIAD**A**A**FAQYYY--.LPKMTA**A**SLAE**T**V**K**SGV**H**A

O00816/1-178 Q**LD**.**ICFLID**..**SS**G**SIG**........IQ**NFRLVKQFL**.H**T**F**L**MV**LP**IG.....P**E**E**VN**N**AVVTYST**.....**DVHLQWDL**QS....PN**A**V**DK**QLAA**HAV**L**E**MPY..-----KK**GSTNT**.**SDGLK**A**CKQI**....L**FT**G**S**R**PGR**EHV---.--P**KLVI**GM**TDGESD**--.....--SDFR**T**.V**RAAKEIRE**L**G**...GI**V**T**VLAVG**HY....VK**HSE**C**RS**MC**G**C**SG**TSD----.----DDSP**C**P**L**YL**RADWG**Q

Q13349/1-186 E**MD**.**IVFLID**..**GS**G**SID**........Q**NDFNQ**M**K**G**FV**.QA**V**MG**QF**EG.......**TDT**LF**AL**M**QYSN**.....LL**KIHFTF**T......Q**FRT**S**PS**Q**QSLVD**P**I**VQ..-----**L**K**G**L**T**F**T**.**ATGI**LT**V**V**TQ**....L**FH**H**K**N**GAR**KSA---.--K**KILIVITDGQ**KYK-.....--DPLE**Y**.S**DV**IP**QAEK**A**G**...**I**IR**YAIGVG**H**A**FQGPT**AR**Q**ELNTISS**A**PP**QDHVFK-.VDNFAA**L**G**S**IQ**KQL**Q**EKI**Y

O49548/1-173 S**ID**.**LV**T**VLD**..L**S**NGG-........-**ANLQ**T**VKHA**M.R**SVIS**L**L**R........**E**M**DRLSIV**V**FST**.....G**SK**R**LMPL**RRMT.AKGR**RS**AR**R**MV**DAL**GGM**E**TT.-----**GGVG**M**SV**.**NDALKKA**V**K**V....V**ED**R**R**EK**N**PS-----.---**T**S**I**F**VL**S**DGQD**Q--.....---PE**AV**.**LKA**KL**NATR**I-...--P**FVVS**T**T**RFSR.PEIP**VH**SVY**IAS**P**GA**LLHAP--.------**L**R**D**A**FTERI**AS**L**L

Q25757_2/1-186 Y**YD**.**LTLILD**..E**S**R**SIT**.......LNKW**KK**D**V**VP**F**A.E**KVLNNLN**ID.....K**D**KI**HVGI**M**RFA**K.....**S**M**KT**D**IGY**EQ....ETRYM**KNDLIKLVRELK**D..--KYG**YGGAT**H**L**.**VDALQYSLK**T....-**FT**R**H**P**NNR**VDA---.--P**KV**T**IL**F**TDGN**E**T**S-.....-KKEK**DI**.**RDVG**LL**YRK**E**N**...**V**K**LIVVGVN**L**A**.....**TE**KS**LK**L**LAG**C**T**ENEE----.CLRVIK**C**EWNDL**T**N**ITKI**L

UN36_CAEEL/1-18 SKN.**VLI**M**LD**..M**S**G**SM**L........GQ**RYE**V**AKQT**T.EA**ILETLS**........**HND**YFN**I**M**TFS**K.....**NT**F**LL**D**G**CNG(5)QAT**MRNK**KA**LRR**KM**DT**Y**Q**S..--EGK**A**EYEK**AL**.**P**L**A**F**S**VL**LDI**....-N**N**GGG**DNN**RGA---.-CE**NVI**M**LITDG**A**PN**--.....-----**AY**.**KK**IF**D**M**YN**ADK..K**V**R**VFT**FL**VGD**EA...I**DFNEVRE**M**AC**N**N**RGYMVH--.VANMAD**V**D**E**K**IHHYIRRM**S

ITB5_HUMAN/1-23 P**VD**.**LYYL**M**D**..L**S**L**SM**-........K**DDLD**NI**RSL**G.T**KL**A**EEM**RKL(25)YQ**TNPCIG**YKL**F**P**N**CVPSFGF**RHLLPL**-.......**TD**RV**DS**FN**E**E**VRK**Q**R**V..-----SR**N**RD**A**P.EG**G**F**D**A**VLQ**A...AVC**K**E**K**I**G**W**R**KDA---.--L**HLLV**FT**TD**DV**P**HIA(27)NQMDYP**SL**.ALL**GEKL**A**E**N**N**...**I**N**LI**FAVTK**N**H.....Y**M**L**Y**K**N**FT**A**LI**PG**TT-----.---VEI**L**DGDS**KNIIQLI**I

Q23564/1-192 W**LD**.**VV**A**VVD**..**NS**IG**MT**........N**G**G**LT**SIAAN**I**.A**SVVSS**G**T**RI(4)SEP**RTTRLGLVTY**NK.....A**A**A**IQ**A**DL**N......Q**YQSLDDVYD**N**V**F**R**ALS..--SVS**TS**EE**S**Y**L**.**ANGL**A**RAED**V....L**E**AG**K**Q**G**Y**N**RTH---.-YQ**RVVIV**YASAYK**G**S-.....--GAL**D**P.VP**VAERLKT**S**G**...**V**T**VITVA**Y**D**Q**D**GD.GAL**LA**D**L**A**KIAS**P**P**YN------.FTNTEDNG**Q**V**I**G**EI**Q**D**A**L**L

E1355658/1-190 T**A**R.**VVLVLD**..**AS**G**SM**-........--**SF**L**YSK**G**VV**.A**DVVERMA**AVAA.QLD**DDGE**MQAW**TFAS**.....**NP**AR**L**P**DL**PL....GD**L**PK**W**L**RLH**VR**V**G**EIS**L(6)KGLHPEQ**V**DMR.**A**V**GIQ**NE**EQK**....V**I**AEVR**A**FVRANPAP.-VP**TLVL**FFS**DG**G**V**Y--.....--RNAE**I**.**ER**EL**REA**V**E**E**P**...**V**FW**Q**F**VGLG**R**S**.....**NYG**V**LER**FD**T**L**PG**RRV----.------DNVG**F**FAVD**DIG**T

Q22783/1-174 N**TD**.**VTIL**M**D**..**N**NNDV**G**.......SAM**EFQ**NQ**CRTI**.A**KLIKTWP**IS.....P**K**LM**E**GEA**I**V**YST**T..DGGQI**VE**N**PF**S.......**YQS**A**S**AFA**N**E**V**MAF**D**DY.---YF**AASP**P**SL**.**T**A**SLQYV**S**Q**N....-----L**G**R**R**RQSR--.--Q**Q**AT**IV**F**T**YS**SS**YS-.....-----**DV**.**QTA**I**E**Y**VTQ**I**G**...GN**LI**I**VAVGGA**.....**DQT**V**LKQL**T**G**N**VV**Y------.---TKT**L**T**T**D**I**F**DQIN**S**L**L

**Consensus/60%** .h**D**.lhbll**D**..s**S**.**S**bs.........spbpbspphl..pllppbs........psspltllpats.....psphbbsb........hpsbsplbphlpplp........hsss*sh.sptlpbsbpb.....bp.p.ssp..........plllll**TDG**pss............sh.bpsspphpp.s...l.lbsltlsss.....sbsblpplts.ss..............h.p.hppbhpbh.

.shtm
